# Supplementary material for: Processing of positive newborn screening results: a qualitative exploration of current practice in England
Source: BMJ Open. 2020 Dec 12;10(12):e044755. doi: 10.1136/bmjopen-2020-044755 (PMC7735110; doi:10.1136/bmjopen-2020-044755)
Supplement: Supplementary data [file bmjopen-2020-044755supp001.pdf]

NBSL Interview Guide

1. In which laboratory do you work

2. What is your job title?

3. When did your lab start processing NBS results?

4. How long have you been involved in processing NBS results?

5. Please can you describe the process you follow when you receive a NBS card for processing in the lab?

   Prompts

   a. Does this differ depending on the condition being screened for?
   b. Is there a time frame this needs to be completed within?

6. For each of the screened conditions, can you clarify what would be considered a positive NBS result?
   a. Sickle cell disease (SCD)
   b. Cystic Fibrosis (CF)
   c. Congenital Hypothyroidism (CHT)
   d. phenylketonuria (PKU)
   e. medium-chain acyl-CoA dehydrogenase deficiency (MCADD)
   f. maple syrup urine disease (MSUD)
   g. isovaleric acidaemia (IVA)
   h. glutaric aciduria type 1 (GA1)
   i. homocystinuria (pyridoxine unresponsive) (HCU)

7. When you have a positive result, what is the next step?

8. Who and how are the clinical team contacted to inform them of a positive NBS result?

   Prompts

   a. Telephone/email/letter?
   b. Does this differ for the different conditions?

9. For each of the screened conditions, what information do you convey to the clinical team?
   a. Sickle cell disease (SCD)
   b. Cystic Fibrosis (CF)
   c. Congenital Hypothyroidism (CHT)
   d. phenylketonuria (PKU)
   e. medium-chain acyl-CoA dehydrogenase deficiency (MCADD)
   f. maple syrup urine disease (MSUD)
   g. isovaleric acidaemia (IVA)
   h. glutaric aciduria type 1 (GA1)
   i. homocystinuria (pyridoxine unresponsive) (HCU)

10. What happens if you are unable to get hold of the relevant clinical team?

11. Do you advise anyone else of the positive NBS result?

Clinical Team Interview Guide

1. In which hospital do you work?

2. What is your job title?

3. For which of the nine screened conditions are you responsible for communicating positive NBS results?

    a. Sickle cell disease (SCD)
    b. Cystic Fibrosis (CF)
    c. Congenital Hypothyroidism (CHT)
    d. phenylketonuria (PKU)
    e. medium-chain acyl-CoA dehydrogenase deficiency (MCADD)
    f. maple syrup urine disease (MSUD)
    g. isovaleric acidaemia (IVA)
    h. glutaric aciduria type 1 (GA1)
    i. homocystinuria (pyridoxine unresponsive) (HCU)

4. How long have you been involved in communicating positive NBS results to families?

5. Who initially communicates the positive NBS result for X condition to the family?

6. How is this information initially communicated to the family?

    Prompts

    a. Letter, from
    b. Email, from
    c. Phone call, from
    d. Other…..

7. When the positive NBS result is communicated to the family in this way, what information is provided?

    Prompts
    a. Where to go
    b. Contact details for
    c. Information about the condition (life expectancy, genetic information, treatment, severity)
    d. Use of information sources (written, verbal, internet)

8. Is any further contact made with the family prior to them being seen in clinic/hospital
   Prompts
   e.g. home visit, follow-up phone call?
   How does this happen?

9. Do you inform anyone else of the result either before or after you speak with the child's family?
   Prompts
   e.g. HV or GP?
   If yes, how, when and why? If no, why not?

10. What happens after the family is told that their child has a positive NBS result for X condition?
    Prompts
    a. Is the child admitted?
    b. How quickly are they seen by the specialist team?
    c. Are any other tests carried out to confirm the diagnosis? When and how?
    d. How often are they followed up?

11. What other members of the MDT do you involve at this stage?
    Prompts
    e.g. refer to dietician, genetic counselling, physiotherapy etc

12. What do you think works well in terms of the way you currently communicate positive NBS results for x condition to the family?

13. If possible, is there anything you would like to change about the way you communicate positive NBS results for x condition to the family?
