# Supplementary figures and images for: Processing of positive newborn screening results: a qualitative exploration of current practice in England
Source: BMJ Open. 2020 Dec 12;10(12):e044755. doi: 10.1136/bmjopen-2020-044755 (PMC7735110; doi:10.1136/bmjopen-2020-044755)

Site 1

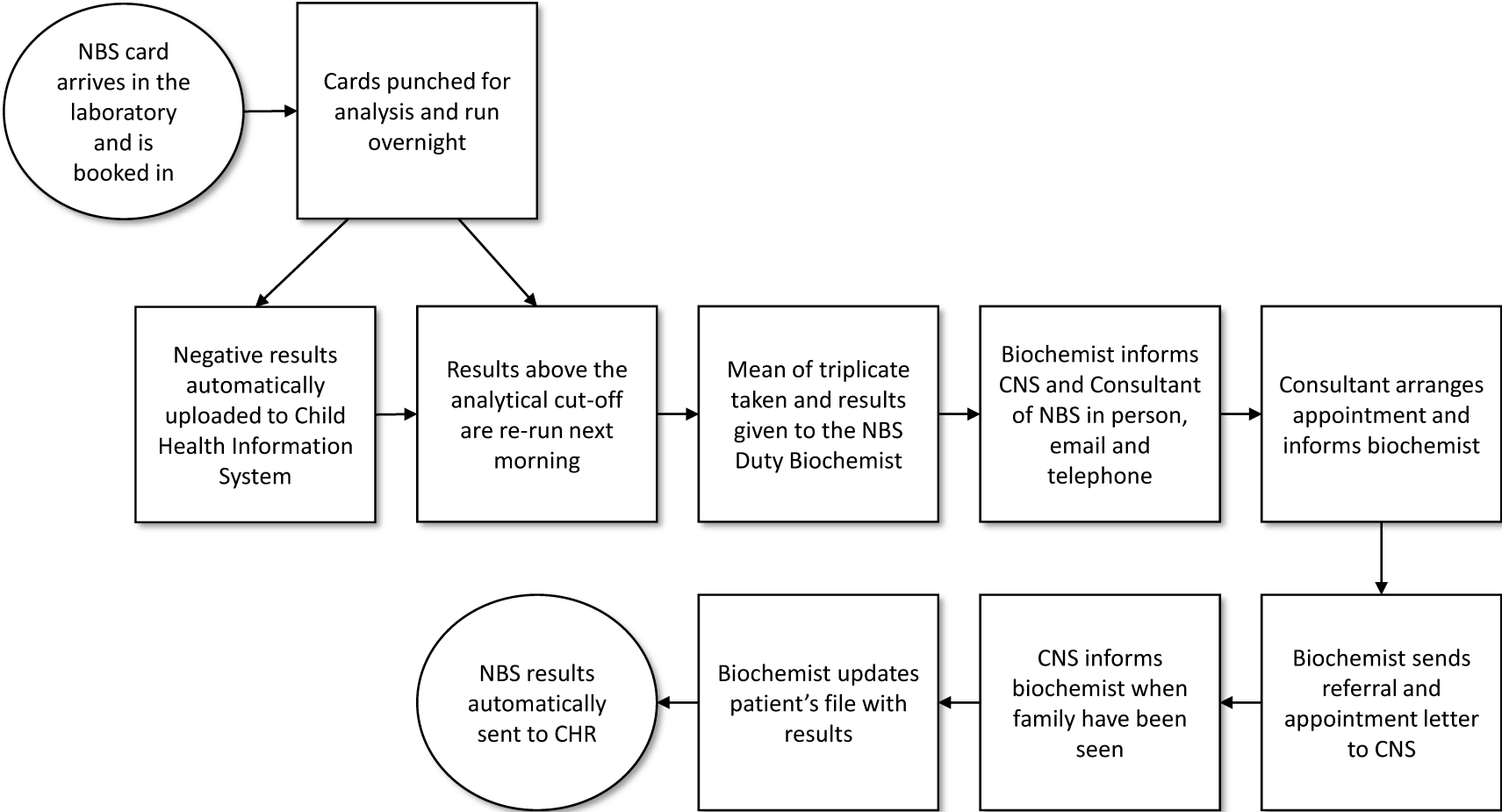

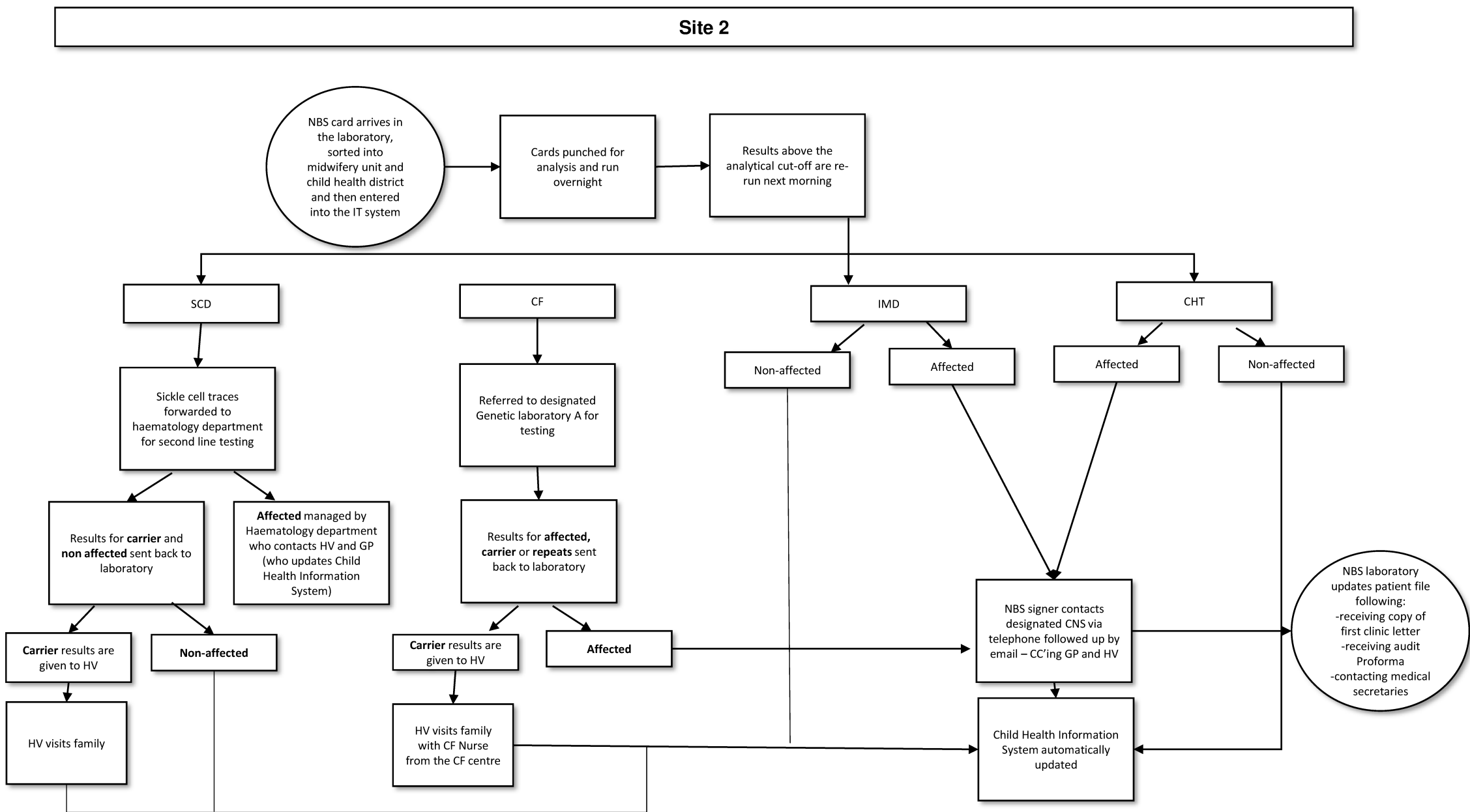

Site 3

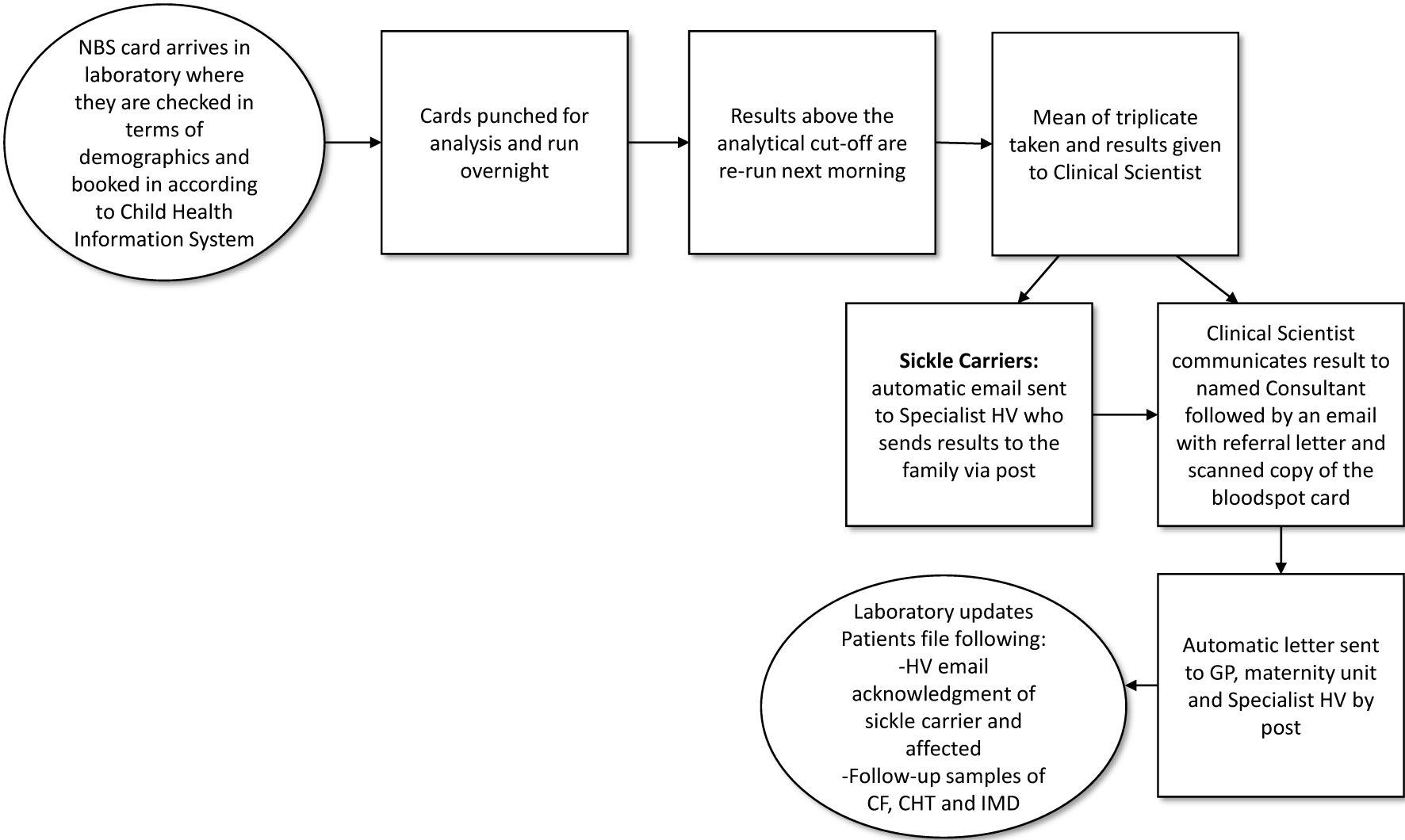

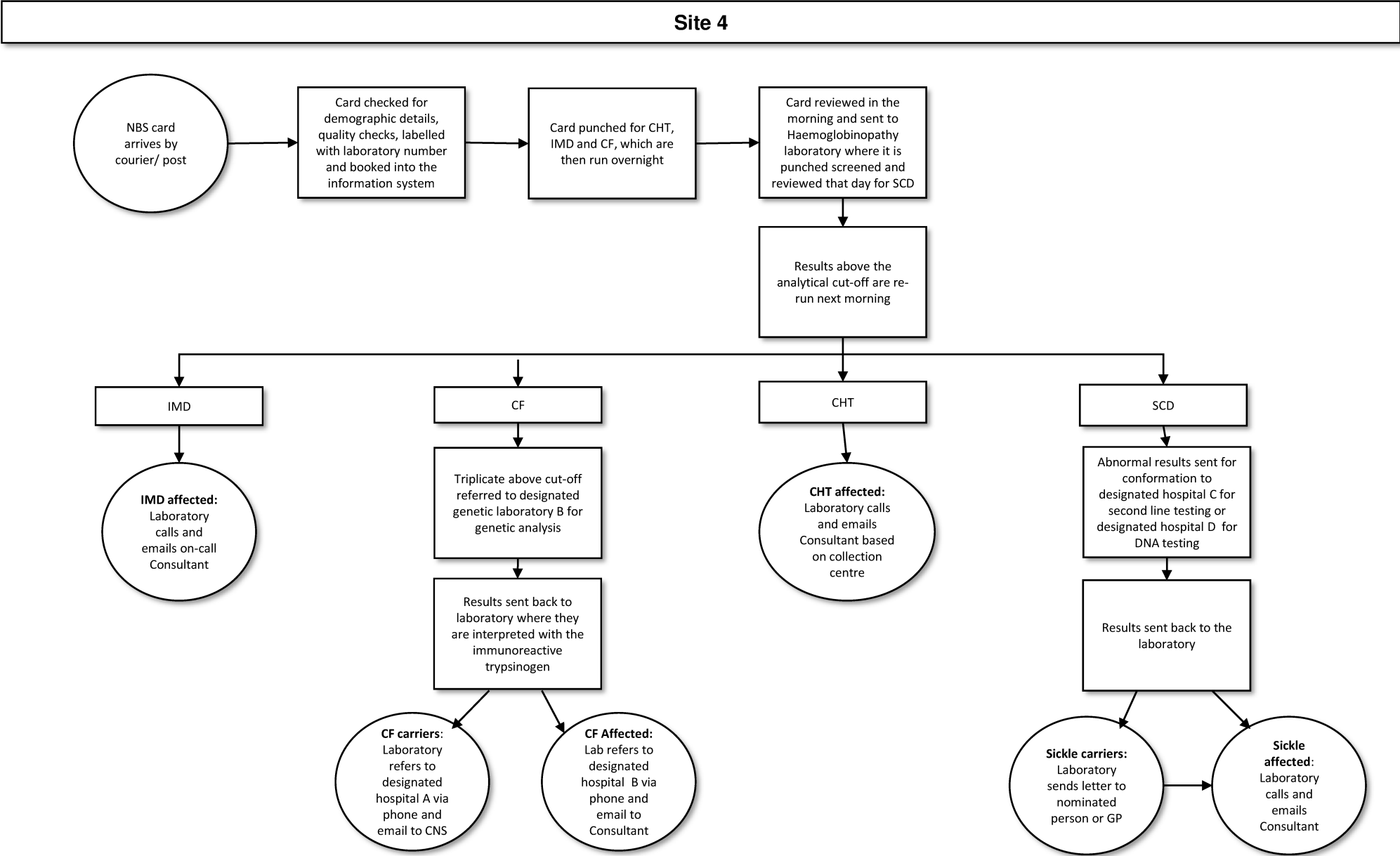

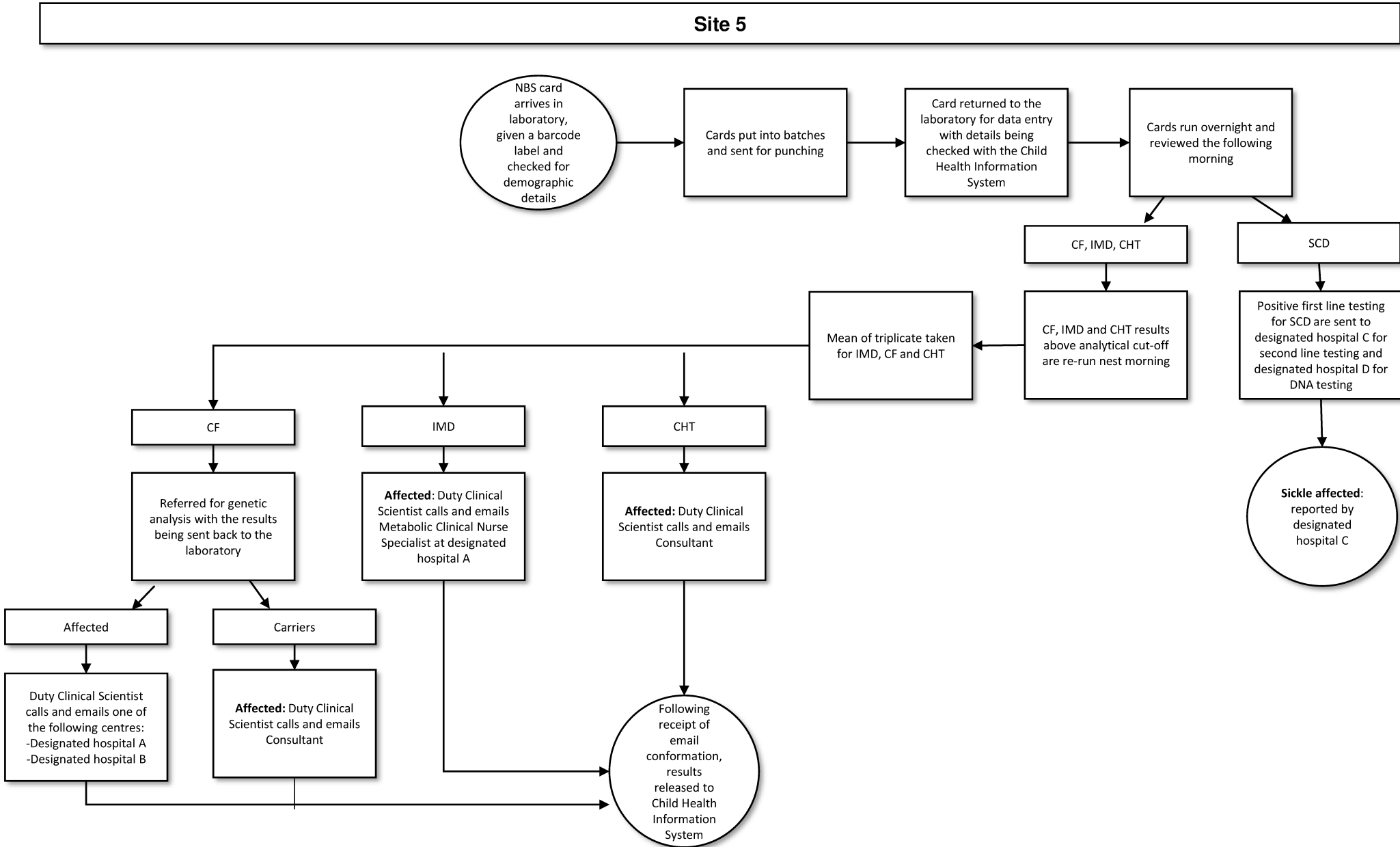

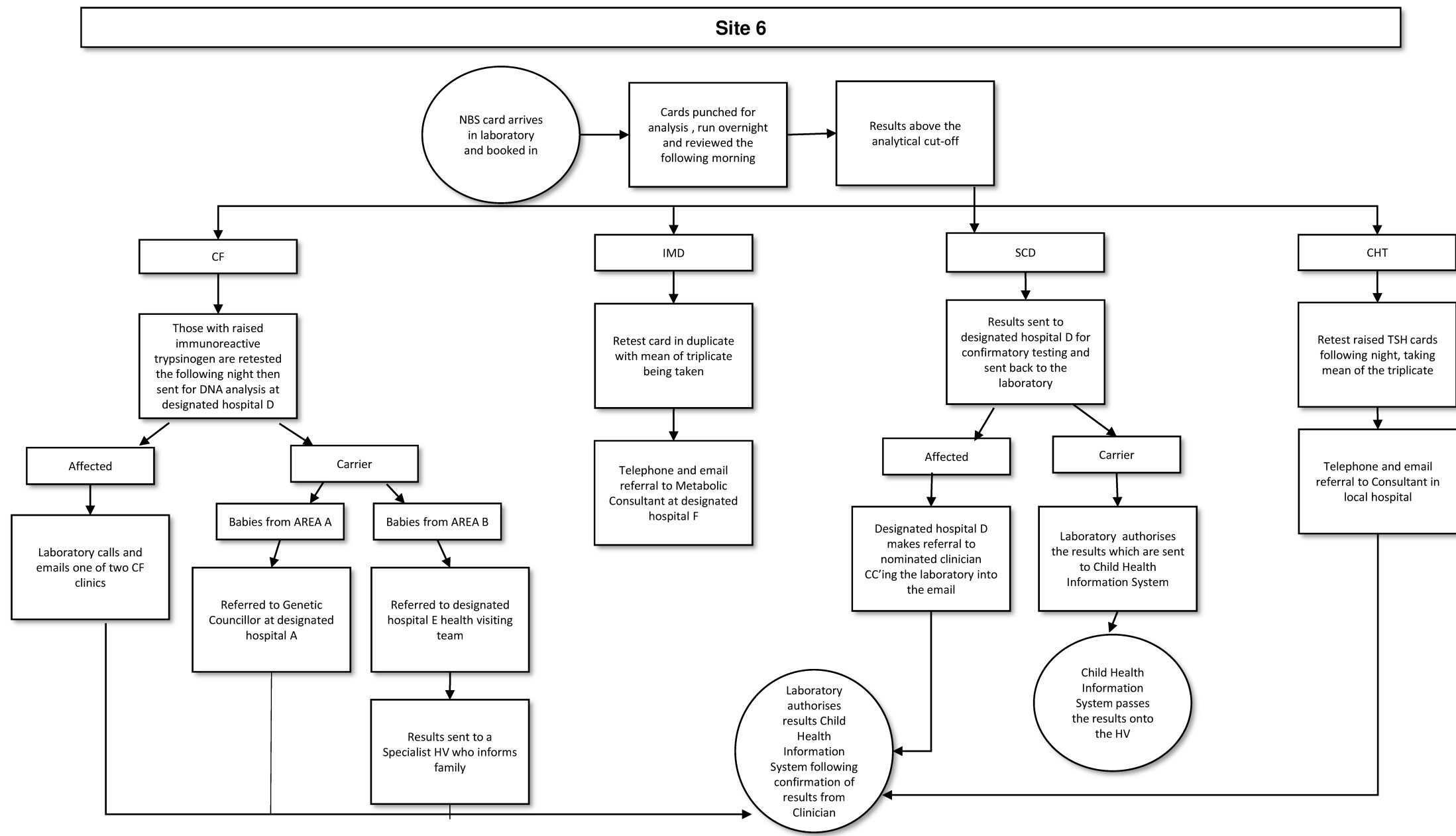

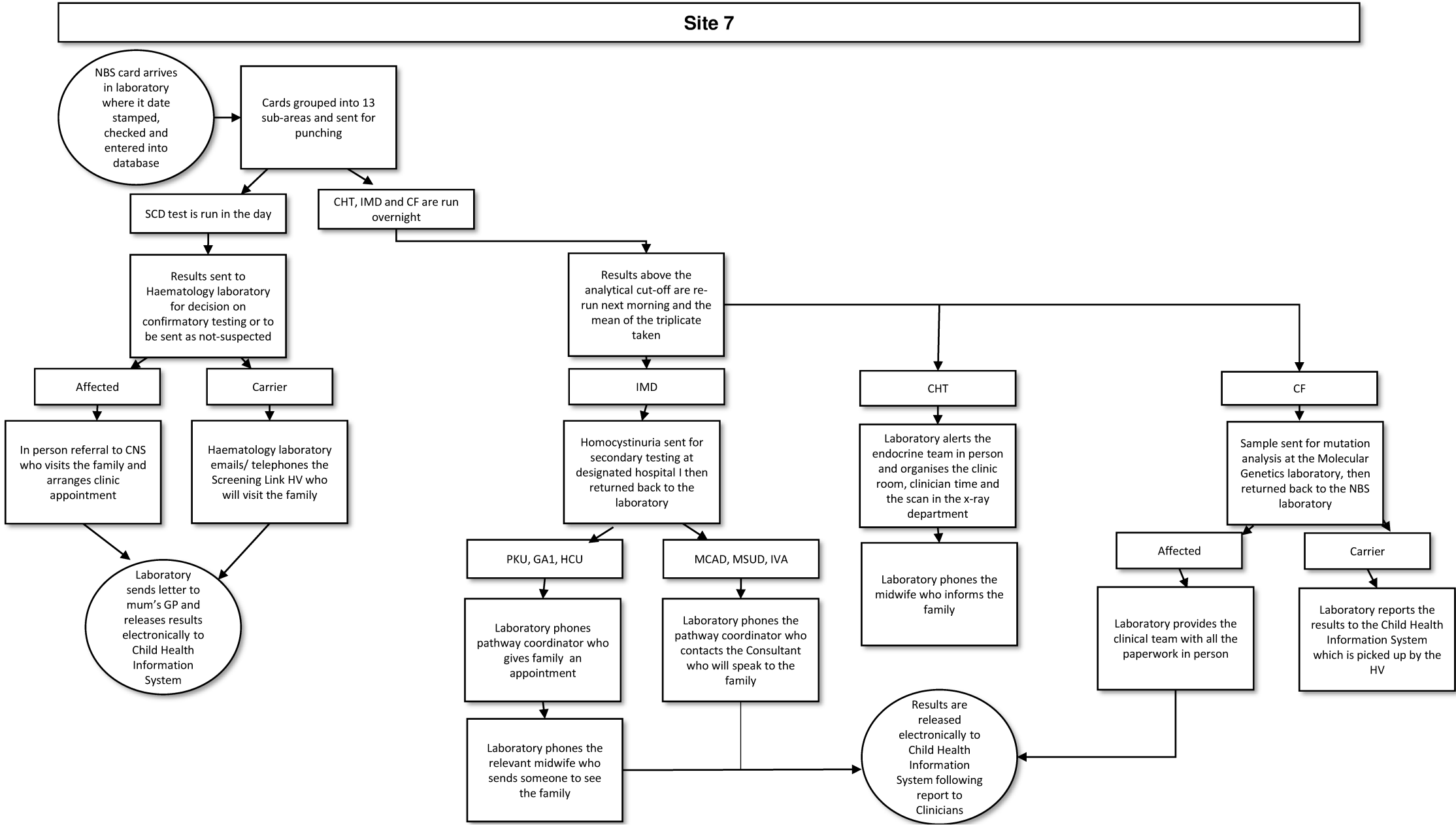

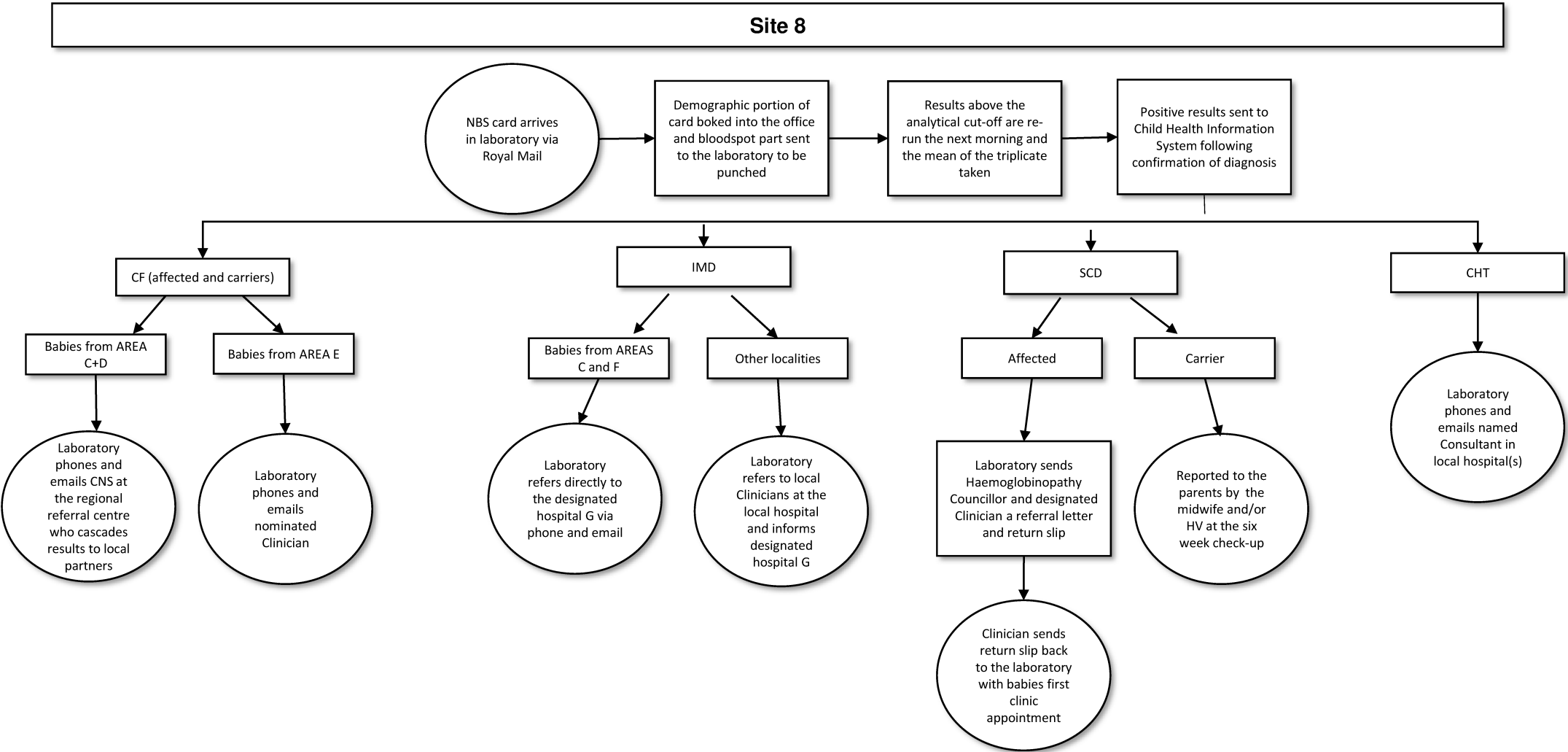

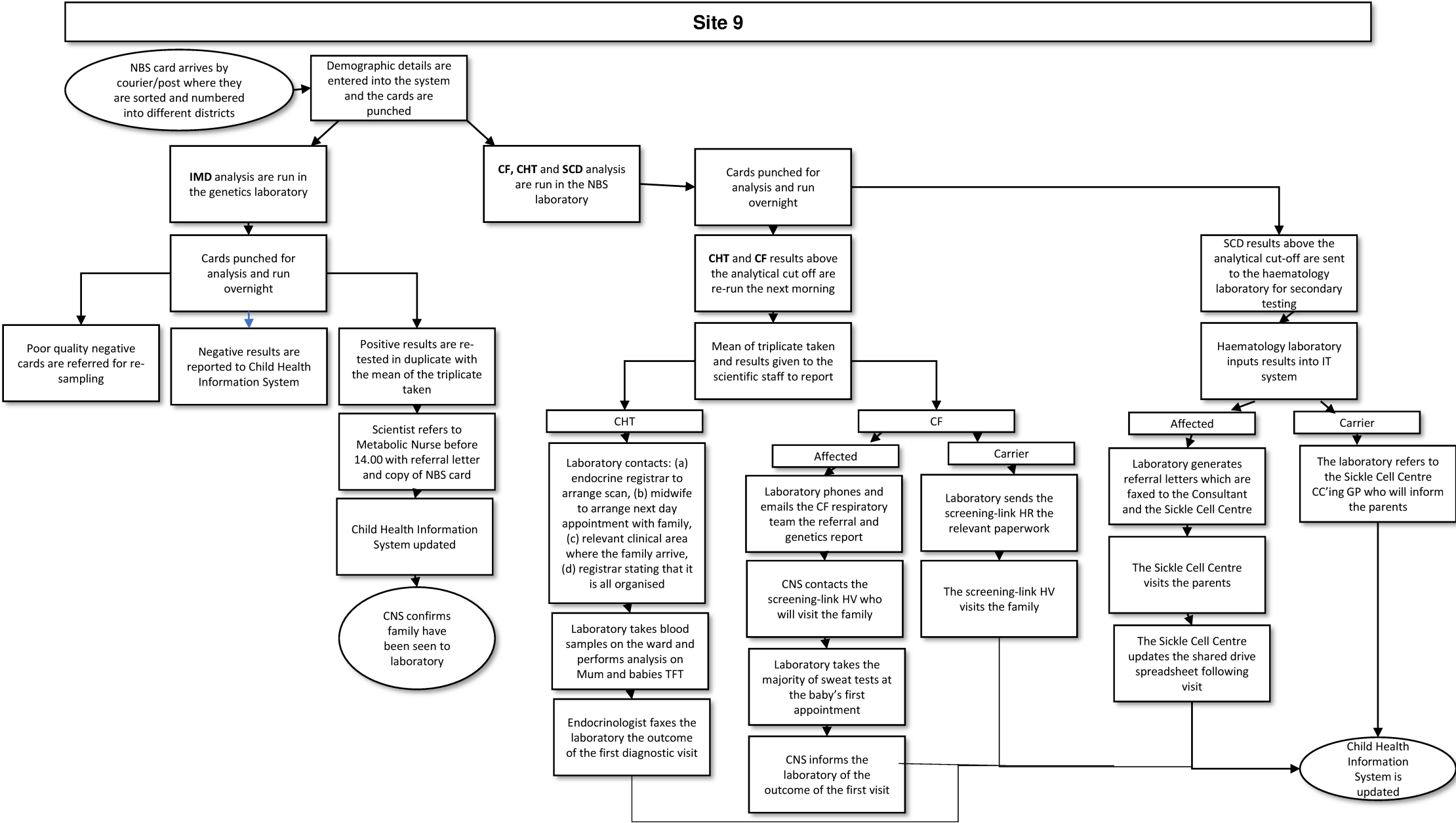

Site 10

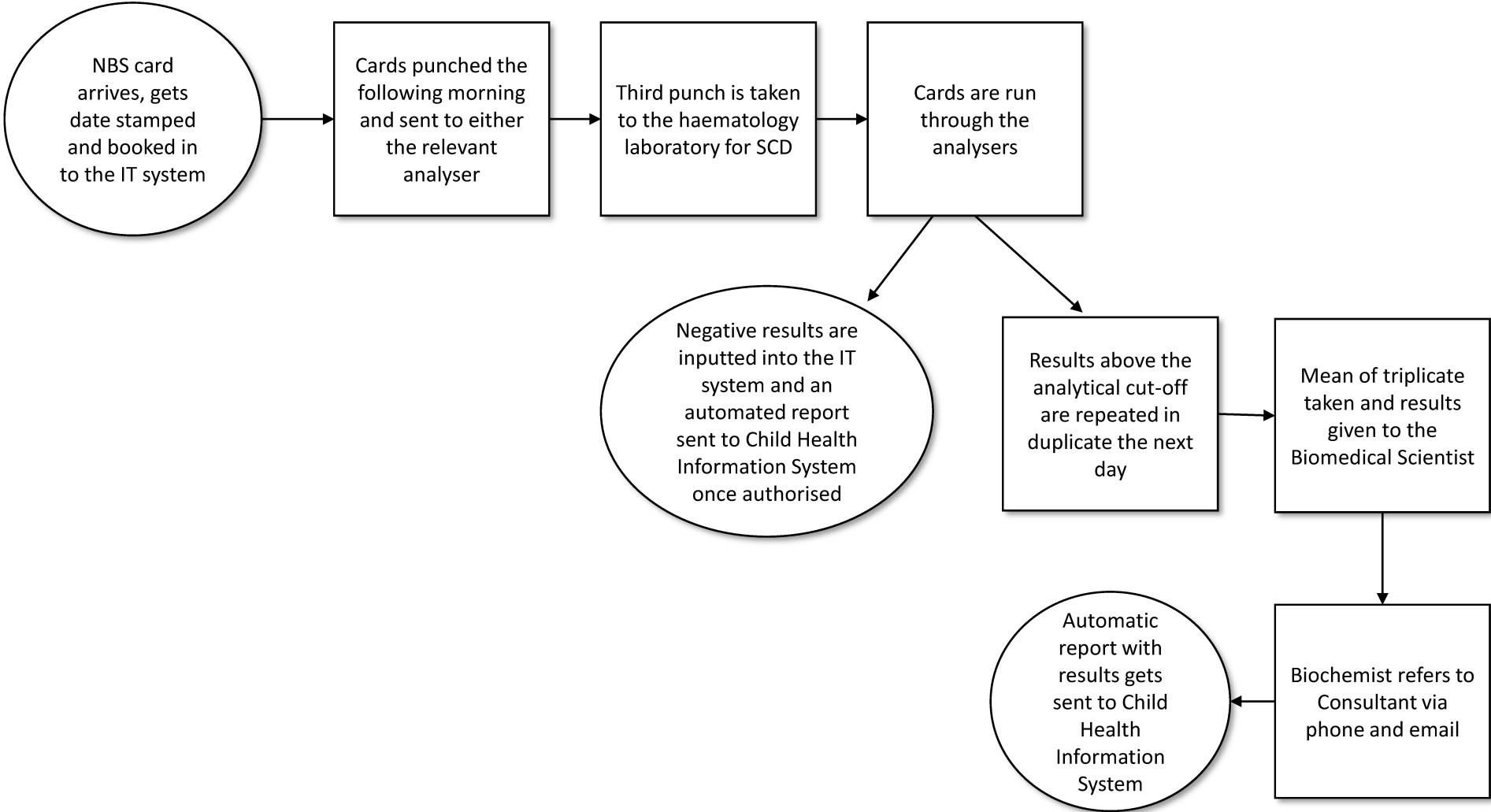

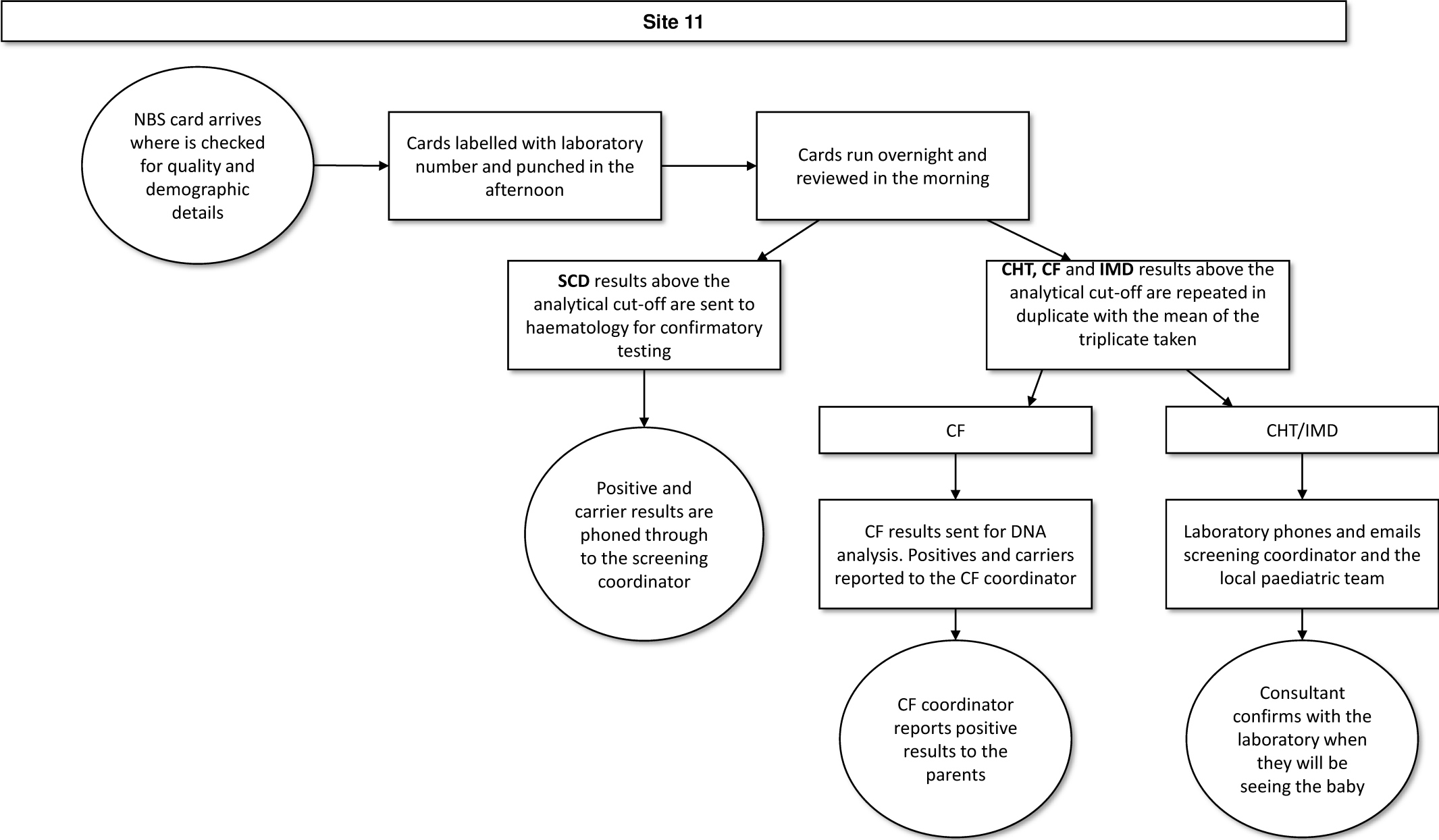

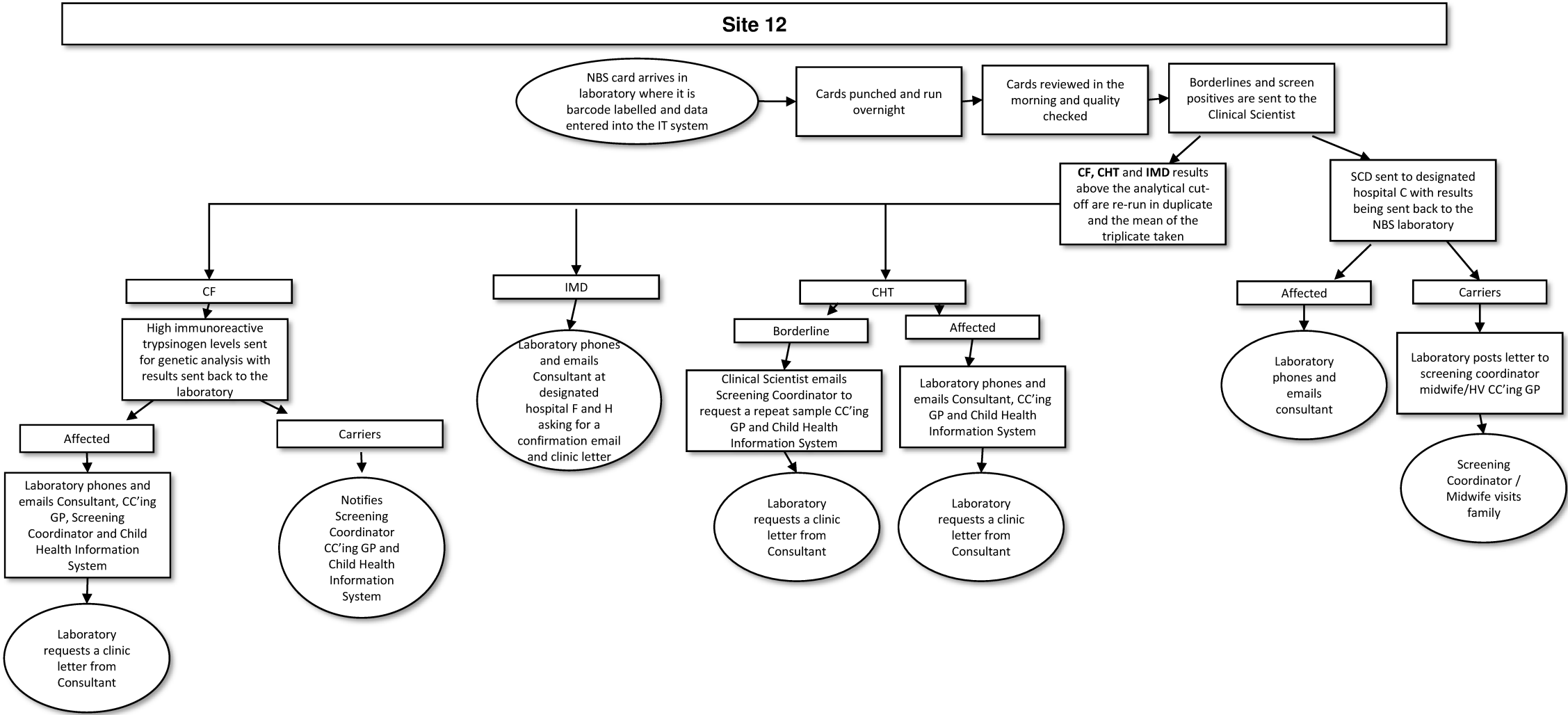

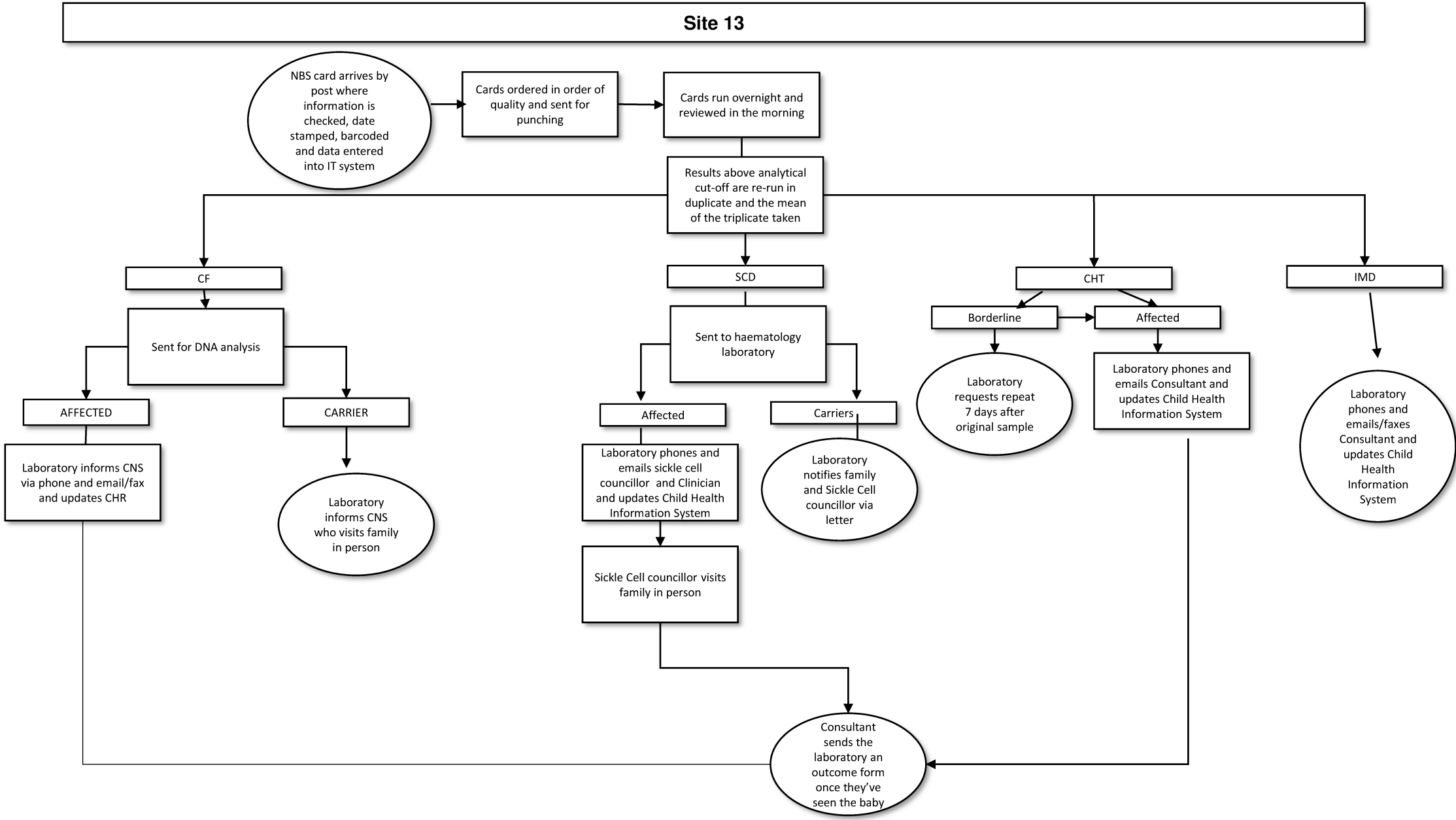

Supplement: Supplementary data [file bmjopen-2020-044755supp002.pdf]
