# Supplementary material for: Processing of positive newborn screening results: a qualitative exploration of current practice in England
Source: BMJ Open. 2020 Dec 12;10(12):e044755. doi: 10.1136/bmjopen-2020-044755 (PMC7735110; doi:10.1136/bmjopen-2020-044755)
Supplement: Author's manuscript [file bmjopen-2020-044755.draft_revisions.pdf]

BMJ Open is committed to open peer review. As part of this commitment we make the peer review history of every article we publish publicly available.

When an article is published we post the peer reviewers' comments and the authors' responses online. We also post the versions of the paper that were used during peer review. These are the versions that the peer review comments apply to.

The versions of the paper that follow are the versions that were submitted during the peer review process. They are not the versions of record or the final published versions. They should not be cited or distributed as the published version of this manuscript.

BMJ Open is an open access journal and the full, final, typeset and author-corrected version of record of the manuscript is available on our site with no access controls, subscription charges or pay-per-view fees (http://bmjopen.bmj.com).

If you have any questions on BMJ Open's open peer review process please email info.bmjopen@bmj.com

BMJ Open

Processing of positive newborn screening results: a qualitative exploration of current practice in England

Journal:	*BMJ Open*
Manuscript ID	bmjopen-2020-044755
Article Type:	Original research
Date Submitted by the Author:	11-Sep-2020
Complete List of Authors:	Chudleigh, Jane; City University, School of Health Sciences<br>Chinnery, Holly; St Mary's University Twickenham, Psychology and Pedagogic Sciences<br>Holder, Pru; City University, School of Health Sciences<br>Carling, Rachel; Guy's and St Thomas' NHS Foundation Trust, Biochemical Sciences, Viapath; King's College London, GKT School of Medical Education<br>Southern, Kevin; Institute of Child Health, Paediatrics<br>Olander, Ellinor; City University, School of Health Sciences<br>Moody, Louise; Coventry University<br>Morris, Stephen; University of Cambridge, Primary Care Unit<br>Ulph, Fiona; University of Manchester<br>Bryon, Mandy; Great Ormond Street Hospital For Children NHS Foundation Trust<br>Simpson, Alan; King's College London, Health Services and Population Research; King's College London
Keywords:	GENETICS, PAEDIATRICS, Organisation of health services < HEALTH SERVICES ADMINISTRATION & MANAGEMENT

SCHOLARONE™
Manuscripts

I, the Submitting Author has the right to grant and does grant on behalf of all authors of the Work (as defined in the below author licence), an exclusive licence and/or a non-exclusive licence for contributions from authors who are: i) UK Crown employees; ii) where BMJ has agreed a CC-BY licence shall apply, and/or iii) in accordance with the terms applicable for US Federal Government officers or employees acting as part of their official duties; on a worldwide, perpetual, irrevocable, royalty-free basis to BMJ Publishing Group Ltd ("BMJ") its licensees and where the relevant Journal is co-owned by BMJ to the co-owners of the Journal, to publish the Work in this journal and any other BMJ products and to exploit all rights, as set out in our licence.

The Submitting Author accepts and understands that any supply made under these terms is made by BMJ to the Submitting Author unless you are acting as an employee on behalf of your employer or a postgraduate student of an affiliated institution which is paying any applicable article publishing charge ("APC") for Open Access articles. Where the Submitting Author wishes to make the Work available on an Open Access basis (and intends to pay the relevant APC), the terms of reuse of such Open Access shall be governed by a Creative Commons licence – details of these licences and which Creative Commons licence will apply to this Work are set out in our licence referred to above.

Other than as permitted in any relevant BMJ Author's Self Archiving Policies, I confirm this Work has not been accepted for publication elsewhere, is not being considered for publication elsewhere and does not duplicate material already published. I confirm all authors consent to publication of this Work and authorise the granting of this licence.

For peer review only

TITLE: Processing of positive newborn screening results: a qualitative exploration of current practice in England

Jane Chudleigh[1]*, Holly Chinnery[2], Pru Holder[1], Rachel S Carling[3], Kevin W Southern[4], Ellinor K Olander[1], Louise Moody[5], Stephen Morris[6], Fiona Ulph[7], Mandy Bryon[8], Alan Simpson[9]

*corresponding author

[1]Centre for Maternal and Child Health Research, City, University of London, EC1V 0HB, UK; [2]Faculty of Sports, Health and Applied Science, St Mary's University Twickenham, TW1 4SX; [3] Biochemical Sciences, Viapath, Guys & St Thomas' NHS Foundation Trust, London, SE1 7EH and GKT School of Medical Education, King's College London, London, WC2R 2LS; [4]Women's and Children's Health, University of Liverpool, L69 3BX, UK; [5]Centre for Arts, Memory and Communities, Coventry University, CV1 5FB, UK; [6]University of Cambridge School of Clinical Medicine, CB2 0SP, UK; [7]Division of Psychology and Mental Health, The University of Manchester, M13 9PL, UK, [8]Paediatric Psychology and Play Services, Great Ormond Street Hospital for Children NHS Foundation Trust, WC1N 3JH, UK, [9]Florence Nightingale Faculty of Nursing, Midwifery & Palliative Care, King's College London SE1 8WA, UK;

j.chudleigh@city.ac.uk; holly.chinnery@stmarys.ac.uk; pru.holder@city.ac.uk; Rachel.Carling@viapath.co.uk; kwsouth@liv.ac.uk; Ellinor.Olander@city.ac.uk; l.moody@coventry.ac.uk; sm2428@medschl.cam.ac.uk; fiona.ulph@manchester.ac.uk; mandy.bryon@gosh.nhs.uk; alan.simpson@kcl.ac.uk;

For peer review only - http://bmjopen.bmj.com/site/about/guidelines.xhtml

ABSTRACT

Objective: To explore current communication practices for positive newborn screening results from the Newborn Bloodspot Screening (NBS) laboratory to clinicians to highlight differences, understand how the pathways are implemented in practice, identify barriers and facilitators and make recommendations for future practice and research.

Design: A qualitative exploratory design was employed using semi-structured interviews

Setting: Thirteen NBS laboratories in England

Participants: Seventy-one clinicians; 22 NBS laboratory staff across 13 laboratories and 49 members of relevant clinical teams were interviewed.

Results: Assurance of quality and consistency was a priority for all NBS laboratories. Findings indicated variation in approaches to communicating positive newborn bloodspot screening results from laboratories to clinical teams. This was particularly evident for congenital hypothyroidism and was largely influenced by local arrangements, resources and the fact individual laboratories had detailed standard operating procedures for how they work. Obtaining feedback from clinical teams to the laboratory after the child had been seen could be challenging and time-consuming for those involved. Pathways for communicating carrier results for cystic fibrosis and sickle cell disease, could be ambiguous and inconsistent which in turn could hamper the laboratories efforts to obtain timely feedback regarding whether or not the result had been communicated to the family. Communication pathways for positive NBS results between laboratories and clinical teams could therefore be time consuming and resource intensive.

Conclusion: The importance placed on ensuring positive NBS results were communicated effectively and in a timely fashion from the laboratory to the clinical team was evident from all participants. However, variation existed in terms of the processes used to report positive NBS results to clinical teams and the people involved. Variant practice identified may reflect local needs, but more often reflected local resources and a more consistent “best practice” approach is required, not just in the UK but perhaps globally.

Trial registration: ISRCTN 15330120

Key words: Newborn bloodspot screening, communication

For peer review only - http://bmjopen.bmj.com/site/about/guidelines.xhtml

Word count: 4595

For peer review only

For peer review only - http://bmjopen.bmj.com/site/about/guidelines.xhtml

Strengths and limitations of this study

- This is the first known study that has explored communication pathways for positive NBS results from the laboratory to clinical teams.
- Participants represented the 13 NBS laboratories in England involved in managing the nine conditions currently included in the NBS programme increasing the transferability of the findings.
- The study design, data collection and analysis were influenced by members of the PPI advisory group and relevant charities.
- The researchers are experienced in this field which may have biased data collection and analysis.

For peer review only

INTRODUCTION

In the UK, Newborn Screening (NBS) comprises a heel prick blood sample taken via a NBS card between days 5-8 of life to identify pre-symptomatic babies that are affected by genetic or congenital conditions[1]. It is known that early diagnosis leads to better health outcomes for the child[1 2]. In the UK, since January 2015, NBS covers nine conditions (See Table 1).

Table 1 Conditions included in the NBS Programme

Name of Condition	Abbreviation
Sickle Cell Disorder	SCD
Cystic Fibrosis	CF
Phenylketonuria	PKU
Homocystinuria	HCU
Glutaric aciduria type 1	GA1
Medium chain acyl CoA dehydrogenase deficiency	MCADD
Maple syrup urine disease	MSUD
Isovaleric aciduria	IVA
Congenital Hypothyroidism	CHT

Each year, around 10,000 babies born in the United Kingdom (UK) have a positive NBS result. These positive NBS results include both babies who will eventually be diagnosed as being affected by one of the nine life changing conditions currently screened for[3 4] (n= approximately 1500) and those who will later be confirmed as gene carriers for Sickle Cell Disorder (SCD) or Cystic Fibrosis (CF) but unaffected by the disease.

Communicating Presumptive Positive NBS results

Newborn Screening Laboratory guides are available for each of the conditions included in the NBS Programme in the UK[5-9]. Positive NBS results are referred to as being 'presumptive positive' (PP) results. That is, the result is presumed to indicate a positive result until further

For peer review only - http://bmjopen.bmj.com/site/about/guidelines.xhtml

diagnostic testing is undertaken to either confirm or rule out the condition being screened for. Within each condition, there is specific information on how PP cases should be reported and communicated. While some aspects of the pathways are similar, some are very different such as the time frame for the first clinic appointment. Table 2 summarises the different pathways as established in the guides, for each of the screened conditions.

The purpose of the current study was to explore current communication practices for positive newborn screening results from the NBS laboratory to clinicians to highlight differences, understand how the pathways are implemented in practice, identify barriers and facilitators and make recommendations for future practice.

For peer review only

For peer review only - http://bmjopen.bmj.com/site/about/guidelines.xhtml

Table 2: UK guidelines for Communicating positive NBS results from laboratories to clinical trams following a presumptive positive (PP) NBS result

Condition(s)	**Method of referral from laboratory to clinical team**	**Time Frame for communicating a PP result to the clinical team**	**Requirements when communicating PP results to families**	**Time frame for first clinic appointment**
Congenital Hypothyroidism[8]	Verbally and in writing by secure email including a link to the standardised diagnostic and initial treatment protocol, to either a paediatric endocrine team or a lead paediatrician with a special interest in CHT	Same or next working day of the definitive NBS result being available	Communicated to the family by an 'informed health professional' who should provide the family with the appropriate information leaflet available via the screening programme and the child's appointment details	Must take place on the same day or the next day after parents are informed of their baby's positive NBS result.
Cystic Fibrosis[5 9]	Refer the baby immediately to a designated person at the appropriate Regional CF Centre. The regional CF centre will usually be responsible for liaising with local CF clinics	Within one working day of the definitive screening result	Parents should be informed by an 'appropriate health professional' but not on a Friday, Saturday or Sunday and that ideally Thursday should be avoided as well. The family should be contacted via a structured	Parents should be offered an appointment on the following day after they are informed of the PP result and the diagnostic assessment within five working days of receiving the result.

For peer review only - http://bmjopen.bmj.com/site/about/guidelines.xhtml

			telephone call in the first instance informing them that the result will be discussed later that day by a healthcare professional with appropriate experience and support to give bad news. Following this, the guidance states that the positive CF screening result may be given at a home visit but sometimes a second structured phone call might be used. In both instances, it is recommended this should be done by a healthcare professional with knowledge of screening and CF	
Sickle Cell Disease[7]	Must be reported as per local pathways, to the clinician and/or designated sickle cell and thalassaemia centre	Not specified	Local protocols must ensure that PP results are given to parents by a trained healthcare professional face-to-face by 28 days of age	The baby should enter the care of a specialist haemoglobinopathy centre by 90 days of age

For peer review only - http://bmjopen.bmj.com/site/about/guidelines.xhtml

Inherited Metabolic Diseases (IMDs) including (MCADD, MSUD, PKU, IVA, HCU, GA1)[6]	Referral of all IMD PP patients should be undertaken in co-ordination with a clinical service that complies with the service specification for a paediatric IMD centre to ensure continuity of specialist care. PP cases should be reported to the specialist clinical team (or designated local team for PKU) verbally and in writing.	Same working day that the PP result is available	Parents should be informed of the PP NBS result face-to-face, usually at home by a member of the relevant IMD team and provided with the relevant 'suspected leaflet' and details of the first clinic appointment (PKU); provided with the relevant 'suspected leaflet' and details of the first clinic appointment via email (MCADD), by a member of the IMD specialist team (HCU) who should also liaise with the appropriate hospital until the baby can be transferred to the specialist IMD centre (MSUD, IVA, GA1)	The baby should be: reviewed at an appropriate hospital within 24 hours (MCADD) or on the next working day via web link or in person (HCU); or the same or next working day (PKU); or taken to an appropriate hospital (if not an inpatient already) where initial assessment will take place and then transferred to a specialist IMD centre on the same day the PP screening result is available. If transfer is not available, the specialist team will liaise with the local hospital to arrange diagnostic testing and supply of dietary supplements (MSUD, IVA, GA1).

For peer review only - http://bmjopen.bmj.com/site/about/guidelines.xhtml

METHODS

A qualitative exploratory design was utilised using semi-structured telephone interviews with laboratory staff employed in the 13 NBS laboratories in England and members of relevant clinical teams notified of positive NBS results from the respective NBS laboratories. This study is part of an ongoing programme of work[10] and was approved by the London Stanmore ethics committee (17/LO/2102).

Patient and Public Involvement

Patient and public involvement (PPI) was instrumental in the design and conduct of this study. Eight parents of babies who had received a positive NBS screening result for one of the nine screened conditions formed a PPI advisory group who met prior to, during and following data collection. Their suggestions were incorporated into the study design, the data collection tools and the data analysis and presentation. In addition, we obtained the views of representatives from charities for the screened conditions including Metabolic Support UK, the British Thyroid Foundation, the CF Trust and the Sickle Cell Society.

Setting

In England, there are 13 Newborn Screening laboratories that process the results for the nine conditions that are currently included in the NBS Programmes, these comprised the study sites.

Inclusion and Exclusion Criteria

Staff employed in NBS laboratories and involved in the processing of positive NBS results and members of relevant clinical teams involved in communicating positive NBS results to parents in the previous 6 months were included. Staff who had not been involved in processing or communicating positive NBS results in the last 6 months or who had personal experience of receiving a positive NBS result were excluded.

Recruitment and sampling

A two-stage sampling approach was employed where participants were first sampled purposively based on their experience with the phenomena of interest, followed by a second stage of snowball sampling where the first participants suggested others. Directors of all 13 NBS-laboratories in England were invited to participate. These were identified through the

For peer review only - http://bmjopen.bmj.com/site/about/guidelines.xhtml

UK Newborn Screening Laboratories Network (http://www.newbornscreening.org/site/laboratory-directory.asp) and were contacted via email by a member of the research team. Directors of newborn screening laboratories were invited to be the Local Principal Investigator for their study site and were asked to provide names and contact details of staff within the laboratory who met the inclusion criteria for the study. These staff members were contacted via email and invited to participate. Representative members of local clinical teams (medical consultants; general paediatricians; nurse specialists; health visitors; specialist screening nurses, genetic counsellors) were identified through individual trust websites and contacted via email to be invited to participate. Those who agreed to participate were also asked to identify other members of their team who they thought should be interviewed to provide further information about the NBS process. These additional potential participants were also contacted by email and invited to participate. Written informed consent was obtained from all participants.

Data Collection

Semi-structured telephone interviews comprising closed and open-ended questions were conducted by JC between June 2018 and February 2019 to identify the approaches used to communicate positive NBS results from NBS laboratories to health professionals. Data were collected on: the mode of communication strategy (face-to-face; letter; telephone; e-mail); the resources involved in each communication strategy; who provides the information and their role; location (co-located or alternative site) of relevant services for each condition.

Data Analysis

The purpose of data analysis was to describe and identify approaches currently used to communicate positive NBS results from NBS laboratories to health professionals. Qualitative data from open-ended questions were analysed using thematic analysis[11] with an inductive approach. Data from laboratory staff and clinical staff were analysed separately. Seven interview transcripts from laboratory staff were coded by two members of the research team (JC and HC) in order to aid coding comparisons and inform and align code development[12]. A code book was developed based on these jointly coded transcripts. A further seven laboratory transcripts were then coded separately by the same two members of the research team using the code book. These separately coded transcripts were compared; inter-coder reliability was 95%. A similar process was followed for the transcripts for clinical staff; inter-code reliability was 92%. Following this, the same two members of the research team coded the remainder of

For peer review only - http://bmjopen.bmj.com/site/about/guidelines.xhtml

the laboratory and clinical staff transcripts using the relevant code books. This was an ongoing, iterative process; new codes were developed and the definition of codes refined as analysis progressed.[13] Once this initial coding had been completed, these codes were then collapsed into themes. Process maps for each condition for each of the 13 NBS laboratories (unit of analysis) were also developed (Supplementary file A).

Positionality and reflexivity

Members of the study team (JC, LM, FU, MB, KWS) have been involved in or continue to undertake a variety of roles and activities associated with the NBS Programme in the UK. It is acknowledged that this could have led to potential bias during data collection and analysis. However, this was balanced by other members of the research team who had previously had minimal involvement in NBS (HC, EKO, AS, SM, PH). Data collection was undertaken by JC and data analysis was mainly undertaken by JC and HC who fall within both camps. Neither JC nor HC were employed in the organisations where data collection was undertaken.

For peer review only

RESULTS

In total, 71 interviews were conducted: 22 with NBS laboratory staff across 13 laboratories and 49 with members of clinical teams. Four eligible participants declined to be interviewed. Demographics of participants can be seen in Table 3.

Table 3: Demographics of participants

**NBS laboratory staff**		
**Profession**		**Number of staff interviewed**
Director of NBS laboratory		9
Consultant Biochemist / haematologist		2
Senior/ Clinical Scientist		11
Length of service	Mean 11.1 years	Range 1.0-22.0 years
Length of interview	Mean 31.12 minutes	Range 13.34-45.05 mins
**Clinical Teams**		
**Profession**		**Number of staff interviewed**
Medical Consultant		21

For peer review only - http://bmjopen.bmj.com/site/about/guidelines.xhtml

Clinical Nurse Specialist<br>Screening Specialist Nurse / Midwife<br>Service Coordinator<br>Paediatric Dietician		21<br>5<br>1<br>1
Length of service	Mean 11.7 years	Range 1.5-23.0 years
Length of interview	Mean 33.17 minutes	Range 10.40-54.58 mins

Themes

Five themes were identified from the data: importance of the screening result; referral from the laboratory to clinical teams; feedback from clinical teams to NBS laboratories; carrier results and resource use and responsibilities. These are explored in detail below and supported by illustrative quotations from interview data.

Importance of the screening result

Assurance of quality and consistency was a clear priority for all NBS laboratories. This applied to all aspects of analysis including quality checks of the NBS card, data entry, first line and if required, second line testing, and timeliness of reporting. There was also a clear appreciation of the significance of the results for families.

"…everybody is aware there is a baby at the end of this, there's a family at the end of this and everybody wants to do the best by that family. So, I think because of that everybody pulls together really well and makes sure, even if it's bad timing, which it usually is, everybody still pulls together to make sure it happens." Site 13

In addition, laboratory staff clearly appreciated the urgency of communicating positive NBS results to clinicians particularly for the Inherited Metabolic Diseases (IMDs) including MCADD, MSUD, PKU, IVA, HCU, GA1.

"Any of the metabolic conditions, if we see a raised result for first time, we will repeat that immediately. So, rather than it going through the system and being repeated, and us only getting the second result the next day, we'd repeat it immediately, so we repeat it effectively offline, and then if it turns out to be positive, we would make the referral straight away." Site 8.

For peer review only - http://bmjopen.bmj.com/site/about/guidelines.xhtml

Referral from the laboratory to clinical teams

Even though national guidelines are available from Public Health England (PHE) for referring positive NBS results from the laboratory to the relevant clinicians, 10 out of 13 of the laboratories created their own templates for this purpose following further development and improvement by staff:

"… we have a proforma that we send …when we get the results. I know there's a national one which we've looked at but we think our own one probably ticks more boxes. From our point of view, it seems to work for us better" Site 7

When a positive NBS result occurs, referrals are made to a range of different clinicians including condition specific consultants, their secretaries, condition specific specialist nurses, specialist screening nurses or screening co-ordinators. The referral process was often based on local arrangements, resources and the fact individual laboratories had detailed standard operating procedures for how they work. These could be quite complex with variation for each condition and often the need to cover a large geographical areas. However, usually these were well understood and implemented.

"…we've got SOPs [standard operating procedures] that define where we need to call for whichever result in whichever place…for our congenital hypothyroidism, we have named consultants at [Hospital E]. We have a name for [Hospital F] and [Hospitals G, H, I and J] where we'll just report to an on-call paediatrician, So, PKU only if they're [Hospital J] babies we report to a [Hospital J] paediatrician, or there's one of two [Hospital J] paediatricians. If we can't get them, then it goes to the metabolic team at the [Hospital A]. Then, MCADD babies if they are [Hospital E] or [Hospital J], then we can report them either to a [Hospital E] named clinician or the [Hospital J] named clinicians. Again, if they're not available, then they'll go down to the metabolic team at the [Hospital A]" Site 3

Many factors were seen to promote effective communication between the laboratory and clinical teams. This included teams being small which meant everyone within the team knew one another and understood each other's roles; the proximity of teams within sites made communication easier between laboratory staff and relevant clinical teams; and good working relationships amongst teams within the same hospital and across sites.

For peer review only - http://bmjopen.bmj.com/site/about/guidelines.xhtml

"I think the close relationship between all the different professional groups for this part of the country works extremely well. Everybody tends to know each other personally; it means there are no barriers to discussion… Now, increasingly, we're working as closely as we can with [Hospital D] in particular…they'll all cover each other, and the labs work quite closely together. We have joint meetings occasionally and to some extent, we have a joint strategic vision because there are specialist lab things that each one of the three of us do. So, we're trying to develop separate areas and compliment rather than compete." Site 9

However, for many laboratories, referral of positive NBS results for CHT was viewed as more problematic. For all of the other screened conditions, dedicated condition specific specialist clinical teams are available to receive the positive NBS result. However, while some babies who have a positive NBS result for CHT will be seen within specialist endocrine teams, CHT is generally viewed as being possible to manage by general paediatricians and therefore, often babies will be referred to local paediatric services. This can mean that in some instances, there is not a named individual to act as a point of contact.

"I'm having to go through a switchboard at a different hospital to try and find somebody who might not be in that hospital because their clinic's in another hospital… I would love it if I just had one person to call about all my hypothyroidism babies, make my life so much easier if I didn't have to phone different GPs and different consultant endocrinologists." Site 10

Concerns were also raised by laboratory staff and members of clinical teams about the equity of care particularly in relation to availability of scans following a positive NBS result for CHT.

"So in terms of equity of care, it would seem that, given that it's a national screening programme, people should be having the same tests for diagnosis as well." Site 4

Conversely, communication of positive CHT NBS results to families was seen as being more straightforward to manage than some of the other conditions included in the NBS programme.

"We do far less visits for CHT babies. They're mainly phoned up and told about the results, and then told when the appointment is and where …It's a lot simpler disorder." Site 1

For peer review only - http://bmjopen.bmj.com/site/about/guidelines.xhtml

Feedback from clinical teams to NBS laboratories

In the UK, following the referral of a baby with a positive screening result, NBS laboratories require feedback from the relevant clinical team once the baby has been seen, assessed and confirmatory testing has been undertaken. There was no consistent unified national approach to providing this feedback, which led to time-consuming efforts by the laboratory staff to obtain information. In contrast, the responsible clinicians often felt they had a clear idea of their duties in this regard.

"…if we don't receive feedback, we have to phone and write letter, and that does take quite a bit of time." Site 1

This was in contrast to the views of clinicians who were responsible for providing the feedback to the NBS laboratories who described steps they took to ensure this information was fed back to the laboratories. This suggests that there may be a mismatch between the information the laboratories actually require, the information clinicians are providing and who is seen to have ownership of the information.

"Then, what I will normally do then is email [the NBS laboratory] back to say, 'Yes, the parents will be attending,' and so on, or, if the parents declined, which has never happened, 'Okay, they're not coming,' and I would assume they would follow up." Site 7

The ability of the laboratory to collate and coordinate feedback from different sources, after a child had been seen, was considered to be particularly challenging and time-consuming for CHT. This was often attributed to the fact that affected babies were often seen in 'local' centres rather than tertiary referral centres due to the treatable nature of the condition. As a result, clinicians from many more localities might be involved in their care. To remedy this, some laboratories had sought local solutions to help them deal with the difficulties associated with feedback for positive NBS results for CHT.

"CHT is much more of a problem in this region because we are not phoning one individual consultant in this region…The CHT is-, I have to chase around a lot more to get that information from other hospitals. They are not very forthcoming, often, at providing the

For peer review only - http://bmjopen.bmj.com/site/about/guidelines.xhtml

information so I have to chase around, and often I have to get my consultant colleague here, the endocrinologist, to help me with that because that can be a real challenge." Site 10

This was in contrast to the IMDs where the conditions were viewed as being more urgent due to the potential life-threatening nature of particularly MCADD, MSUD and IVA. Also, because affected babies were only seen at tertiary centres where often the NBS laboratory staff worked closely both physically and personally with IMD clinical team members.

"…for the IMD conditions, because the consultant that sees them is based in the same hospital as me [Laboratory Director] and the fact he is my colleague, we work together to provide the IMD clinical service, I do get that information from him. I also can see that the diagnostic blood tests have been received in our laboratory. So, I know those babies have been seen." Site 10

Carrier results

Pathways for communicating carrier results to families were viewed by some laboratories as ambiguous and inconsistent. NBS laboratories expressed concern regarding whether parents had been informed of their baby's SCD or CF carrier status and by whom as this information was often difficult for them to ascertain. Carriers are healthy children who carry a single faulty gene. The most commonly received positive newborn screening result is for carrier status for SCD. Communication pathways can include a range of health professionals including sickle cell coordinators, sickle cell counsellors, general practitioners, and/or health visitors via telephone, letter, home visit or during the baby's health review when they are six weeks of age. Therefore, although this represents a different result scenario (the baby is considered healthy), there is still evidence of inconsistency in terms of the pathways used.

"… for a child who has who has been found to have a carrier status of sickle cell, we just correspond with the health visitor and the GP in a non-urgent way because that is something that just needs to be added to their record but probably won't impact on their health greatly." Site 2

The purpose of the CF NBS protocol in the UK is to maximise the detection of affected individuals (those with two disease causing mutations of the CF transmembrane regulator

For peer review only - http://bmjopen.bmj.com/site/about/guidelines.xhtml

(CFTR) gene) while minimising the detection of unaffected carriers of CF. However, when carrier results are identified, these too need to be communicated to families. Similar to SCD, this occurs in a range of ways by different people including general practitioners, health visitors, specialist nurses and genetic counsellors via telephone or home visits.

"CF carriers, we've got two specialist nurses within our screening lab and they actually go and do a home visit with the families and give them the CF carrier result in person" Site 13

Feedback to the NBS laboratories regarding whether the parents had been informed of their baby's SCD or CF carrier status and by whom was also of concern to some laboratories.

Resource use and responsibilities

NBS cards are used to collect blood from infants and information from their parents when the NBS heel prick sample is taken when the child is approximately 5 days of age. Procedures for processing NBS cards for the nine conditions currently included in the NBS Programme were considered to be transparent and efficient; clinicians within the NBS laboratories were able to discuss these in detail and seemed satisfied with how the laboratory guidelines were operationalised.

However, operationalising the communication aspect of the NBS pathways had clear implications in terms of resources; communicating positive newborn screening results for the screened conditions could be time consuming for a range of reasons. These included, not being able to contact the appropriate person or needing to wait for the appropriate busy clinician to return a telephone call.

"Sometimes…you'd speak to a secretary and you'd be waiting for a doctor to get back to you. So, it can take a few hours from when you'd started to try and process that, depending on how quickly get back to you." Site 12

"..it does sometimes feel like a bit of a battle trying to get hold of someone." Site 3

Again, this was also seen as being condition specific**.**

For peer review only - http://bmjopen.bmj.com/site/about/guidelines.xhtml

"Once I know about a positive result [for CF or one of the metabolic conditions], ten, fifteen minutes….for CHT, I might be chasing around for an hour." Site 10

Due to the time-consuming nature of referring positive NBS results to the relevant professional or team, specialist screening professionals were highly valued by laboratory staff due to their knowledge of the conditions but also their ability to provide a link between the laboratory and the clinical teams. However, this service was not universally available.

"We don't have anything luxurious like a screening specialist nurse or anything like that, which I think probably would be extremely useful, but we don't." Site 2

Once the screening result has been received by the relevant member of the clinical team, a large amount of time is dedicated to organising and preparing for the family to be seen by the clinical team for the first time. The responsibility for this is shared and might be undertaken by the laboratory team, the consultant's secretary and/or the consultant or clinical nurse specialist for the specific condition. This was often centre specific and depends on local arrangements and resources.

Although quite unusual, in some areas, the NBS laboratory would be responsible for arranging the appointment and sometimes the diagnostic tests. In others, this would be the responsibility of the specialist screening coordinator, screening nurse or screening health visitor. In other centres, this would be the responsibility of members of the relevant clinical team such as the consultant or specialist nurse or midwife.

Communication pathways:

Communication pathways for the 13 newborn screening laboratories in England for positive NBS results were developed from the interview responses. These described how positive NBS results were communicated from the NBS laboratory to parents via the clinical team and highlighted when variations occurred (Supplementary file A).

DISCUSSION

The findings of this study indicate that NBS laboratory staff are acutely aware of the significance of a positive NBS result and the potential impact this could have on the family, even though in the majority of cases, they do not have direct contact with the family. Despite this, some challenges existed when communicating results from laboratories to relevant

For peer review only - http://bmjopen.bmj.com/site/about/guidelines.xhtml

clinicians. These could involve time-consuming processes being employed to ensure the NBS ‘communication loop’ from the laboratory to clinical teams to families and back to the laboratory was closed.

The communication pathway for positive NBS results starts in the laboratory via relevant clinical teams and ends with the family of the affected child. Many studies have explored communication of positive NBS results to families[14-19] but none have explored communication of NBS results between the laboratory and clinical teams involved in this process.

Although national proformas for the communication of positive NBS results exist, most laboratories had developed their own proformas, designed to meet local needs more explicitly. However, this meant that for clinical teams who received positive NBS results from more than one laboratory, the information and format used varied. It is known that variations exist both nationally and internationally in terms of the approaches used to communicate positive NBS results to families [14-19] but this would also seem to extend to the approaches used to communicate positive NBS results between clinicians. In addition, laboratory staff could spend considerable time trying to locate and make a referral to the correct clinician. Finally, obtaining the necessary feedback from clinical teams to enable the laboratories to complete their reporting processes could also be time consuming and challenging. Although this is unlikely to influence communication with the family, it is important to ensure information relating to every child’s NBS journey is documented in a timely fashion and is available to relevant professionals involved in the child’s care. Interestingly, providing this feedback was not seen as being an issue by clinical staff which suggests that they may not be aware of the information that is needed and could explain why in some cases this is not fed back in a timely manner.

Communication of positive NBS results between the laboratory and clinical teams, occurred in a range of ways. In some instances, this was condition specific but often this was determined by local arrangements and availability of resources. Again, this reflects variations observed when positive NBS results are communicated to families [14-19]. Availability of specialist screening nurses who could act to bridge these processes by receiving the screening result, arrange the follow-up required and deliver the positive NBS result to the family, were highly valued by both laboratory staff and members of clinical teams and viewed as an

For peer review only - http://bmjopen.bmj.com/site/about/guidelines.xhtml

example of good practice. Communication pathways for carrier results, the most common outcome for NBS screening particularly for SCD, also varied significantly by condition and locality.

Communicating positive NBS results for CHT seemed to be particularly problematic perhaps because communication is not always via a specialist clinical team due to CHT being viewed as manageable by general paediatricians. Different models of care are in operation throughout the country and how these operate seemed to be influenced by local arrangements and resources but also to some extent historical influences. This often led to difficulties from the laboratory perspective: who the correct person was to refer a baby with suspected CHT to; a lack of confidence on occasions once the referral had been made; and concern that the child may not be followed up according to national guidelines. Other conditions have dedicated clinical teams that focus solely on that condition (CF) or range of conditions (SCD and the metabolic conditions) whereas babies with CHT are seen by endocrine teams who manage a range of other unrelated conditions in addition to CHT. This is in contrast particularly to the IMDs where the close working relationships both physically and personally between laboratory staff and clinical teams were seen to enhance the referral process.

Strengths and Limitations:

The current study has numerous strengths. This is the first known study that has explored communication pathways for positive NBS results from the laboratory to clinical teams. Participants represented the 13 NBS laboratories in England involved in managing the nine conditions currently included in the NBS programme increasing the transferability of the findings. In addition, the study design, data collection and analysis were influenced by members of the PPI advisory group and relevant charities. The researchers are experienced in this field which may have biased data collection and analysis.

Recommendations for practice

The findings of this study suggest a range of recommended routes, with some key requirements for communication of positive NBS results from laboratories to clinicians, allowing for local variation and complexity, would be beneficial. This would ensure information sharing amongst the range of professionals involved nationally is optimised following a positive NBS result. This would include information required by laboratories to complete their processes, which would reduce demand on resources currently used to ensure

For peer review only - http://bmjopen.bmj.com/site/about/guidelines.xhtml

this information is collected. Further research is needed to explore communication pathways for positive NBS results for CHT to ensure the process is streamlined and clear communication pathways are in place. Whilst it is acknowledged that a single approach for processing CHT results may not be appropriate or necessary, transparency and complete information such as named contact individuals may help to ensure the process is less labour intensive particularly from a laboratory perspective.

CONCLUSIONS

The importance of ensuring timely and effective communication of positive NBS results from laboratories to families via relevant clinicians was seen as a priority for all involved. Variation exists in terms of proformas and processes used to report positive NBS results to clinical teams, the approaches used, and the people involved. This was often determined by local arrangements and resources. These different approaches could be time consuming and challenging. Despite these challenges, ensuring quality and consistency was a clear priority for all NBS laboratories.

For peer review only

For peer review only - http://bmjopen.bmj.com/site/about/guidelines.xhtml

Acknowledgements: The authors would like to thank the Newborn Screening Laboratory Directors in England for agreeing to act as local Principal Investigators for this study. The authors would also like to thank all the parents in the Public and Patient Involvement Advisory Group for this study for their invaluable input.

Contributors: JC made substantial contributions to the conception and design of the work. She acquired and interpreted the data for the work. She was involved in drafting the work, approved the final version to be published and agrees to be accountable for all aspects of the work in ensuring that questions related to the accuracy or integrity of any part of the work are appropriately investigated and resolved. AS and SM made substantial contributions to the conception and design of the work. They were involved in drafting the work, approved the final version to be published and agree to be accountable for all aspects of the work in ensuring that questions related to the accuracy or integrity of any part of the work are appropriately investigated and resolved. EKO and HC were involved interpreting the data for the work and revising the work critically for important intellectual content, approved the final version to be published and agree to be accountable for all aspects of the work in ensuring that questions related to the accuracy or integrity of any part of the work are appropriately investigated and resolved. LM, FU, MB, RSC, MAP and KWS made substantial contributions to the conception and design of the work. They were involved in drafting the work, approved the final version to be published and agree to be accountable for all aspects of the work in ensuring that questions related to the accuracy or integrity of any part of the work are appropriately investigated and resolved.

Funding: This study is funded by the National Institute for Health Research (NIHR) [Health Services and Delivery Research (project reference 16/52/25)]. The views expressed are those of the authors and not necessarily those of the NIHR or the Department of Health and Social Care.

Competing interests: None declared.

Ethics approval: This study was approved by the London Stanmore ethics committee, reference 231291

For peer review only - http://bmjopen.bmj.com/site/about/guidelines.xhtml

Data Sharing statement: Data are available upon reasonable request from the corresponding author subject to restrictions to preserve anonymity and personal privacy [JC]. These data are not publicly available as they contain information that could compromise research participant privacy/consent

Data will be available beginning 1 year and ending 5 years after publication to researchers who propose a methodologically sound proposal. Proposals should be directed to j.chudleigh@city.ac.uk. To gain access, data requesters will need to sign a data access agreement.

For peer review only

References

1. UK Newborn Screening Programme Centre. Health Professional Handbook: A guide to newborn blood spot screening for healthcare professionals. London: UK Newborn Screening Programme Centre, 2012:1-56.
2. Bush A. Newborn screening for cystic fibrosis – benefit or bane? *Paediatric respiratory reviews* 2008;9:301-2.
3. Public Health England. Newborn Blood Spot Screening Programme in the UK: Data Collection and Performance Analysis Report 2016 to 2017. London, 2018:1-65.
4. Public Health England. NHS Sickle Cell and Thalassaemia Screening Programme Data Report 2015/16: Trends and performance analysis. London, 2017:1-81.
5. Public Health England. NHS Newborn Blood Spot Screening Programme: Managing positive results from cystic fibrosis screening. London, 2017:1-20.
6. Public Health England. NHS Newborn Blood Spot Screening Programme A laboratory guide to newborn blood spot screening for inherited metabolic diseases. London, 2017:1-140.
7. Public Health England. NHS Sickle Cell and Thalassaemia Screening Programme Handbook for newborn laboratories. London, 2017:1-32.
8. UK National Screening Committee. A Laboratory Guide to Newborn Screening in the UK for Congenital Hypothyroidism 1st Edition ed. England, 2014:1-39.
9. UK Newborn Screening Programme Centre. A Laboratory Guide to Newborn Screening in the UK for Cystic Fibrosis. 4th Edition ed. London, 2014:1-35.
10. Chudleigh J, Bonham J, Bryon M, et al. Rethinking Strategies for Positive Newborn Screening Result (NBS+) Delivery (ReSPoND): a process evaluation of co-designing interventions to minimise impact on parental emotional well-being and stress. *Pilot Feasibility Stud* 2019;5:108. doi: 10.1186/s40814-019-0487-5
11. Braun V, Clarke V. Using thematic analysis in psychology. *Qualitative Research in Psychology* 2006;3(2):77-101.
12. Milford C, Kriel Y, Njau I, et al. Teamwork in Qualitative Research: Descriptions of a Multicountry Team Approach. *International Journal of Qualitative Methods* 2017;16:1-10.
13. DeCuir-Gunby JT, Marshall PL, McCulloch AW. Developing and Using a Codebook for the Analysis of Interview Data: An Example from a Professional Development Research Project. *Field Methods* 2011;23(2):136-55.
14. Chudleigh J, Buckingham S, Dignan J, et al. Parents' Experiences of Receiving the Initial Positive Newborn Screening (NBS) Result for Cystic Fibrosis and Sickle Cell Disease. *J Genet Couns* 2016;25(6):1215-26. doi: 10.1007/s10897-016-9959-4
15. Parker H, Qureshi N, Ulph F, et al. Imparting carrier status results detected by universal newborn screening for sickle cell and cystic fibrosis in England: a qualitative study of current practice and policy challenges. *BMC Health Serv Res* 2007;7:203. doi: 1472-6963-7-203 [pii] 10.1186/1472-6963-7-203 [published Online First: 2007/12/15]
16. Ulph F, Cullinan T, Qureshi N, et al. The impact on parents of receiving a carrier result for sickle cell or cystic fibrosis for their child via newborn screening. *Eur J Hum Genet* 2014;22
17. Chudleigh J, Ren CL, Barben J, et al. International approaches for delivery of positive newborn bloodspot screening results for CF. *Journal of cystic fibrosis : official journal of the European Cystic Fibrosis Society* 2019;18(5):614-21. doi: 10.1016/j.jcf.2019.04.004
18. Kai J, Ulph F, Cullinan T, et al. Communication of carrier status information following universal newborn screening for sickle cell disorders and cystic fibrosis: qualitative study of experience and practice. *Health Technol Assess* 2009;13(57):1-82, iii. doi: 10.3310/hta13570 [published Online First: 2009/12/02]
19. Ulph F, Cullinan T, Qureshi N, et al. Parents' responses to receiving sickle cell or cystic fibrosis carrier results for their child following newborn screening. *Eur J Hum Genet* 2015;23(4):459-65. doi: DOI 10.1038/ejhg.2014.126

For peer review only - http://bmjopen.bmj.com/site/about/guidelines.xhtml

For peer review only

For peer review only - http://bmjopen.bmj.com/site/about/guidelines.xhtml

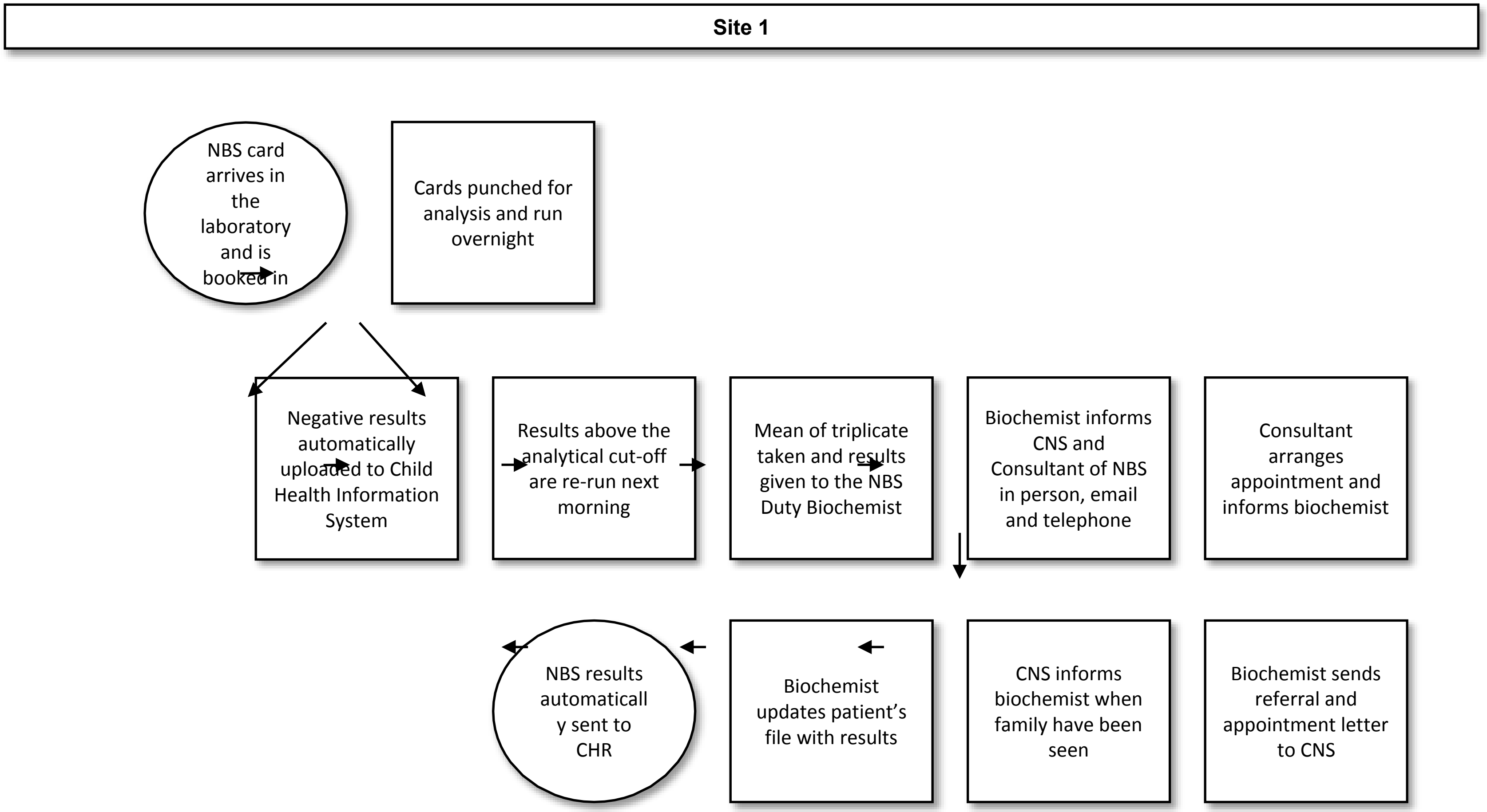
Site 1
NBS card arrives in the laboratory and is booked in
Cards punched for analysis and run overnight
Negative results automatically uploaded to Child Health Information System
Results above the analytical cut-off are re-run next morning
Mean of triplicate taken and results given to the NBS Duty Biochemist
Biochemist informs CNS and Consultant of NBS in person, email and telephone
Consultant arranges appointment and informs biochemist
Biochemist sends referral and appointment letter to CNS
CNS informs biochemist when family have been seen
Biochemist updates patient's file with results
NBS results automaticall y sent to CHR

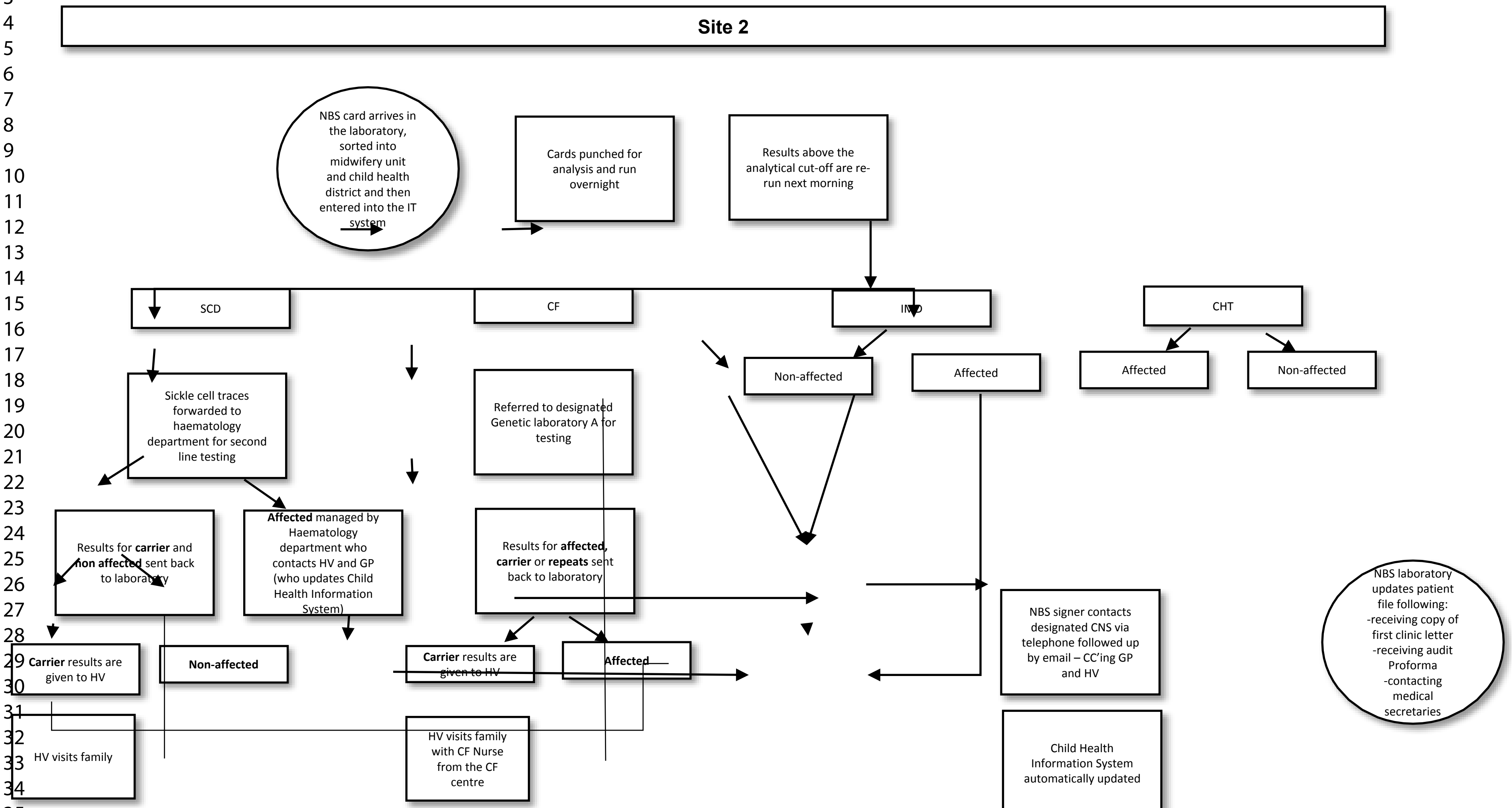
Site 2
NBS card arrives in the laboratory, sorted into midwifery unit and child health district and then entered into the IT system
Cards punched for analysis and run overnight
Results above the analytical cut-off are re-run next morning
SCD
CF
IMD
CHT
Non-affected
Affected
Affected
Non-affected
Sickle cell traces forwarded to haematology department for second line testing
Referred to designated Genetic laboratory A for testing
Results for carrier and non affected sent back to laboratory
Affected managed by Haematology department who contacts HV and GP (who updates Child Health Information System)
Results for affected, carrier or repeats sent back to laboratory
NBS signer contacts designated CNS via telephone followed up by email – CC'ing GP and HV
NBS laboratory updates patient file following: -receiving copy of first clinic letter -receiving audit Proforma -contacting medical secretaries
Carrier results are given to HV
Non-affected
Carrier results are given to HV
Affected
HV visits family
HV visits family with CF Nurse from the CF centre
Child Health Information System automatically updated

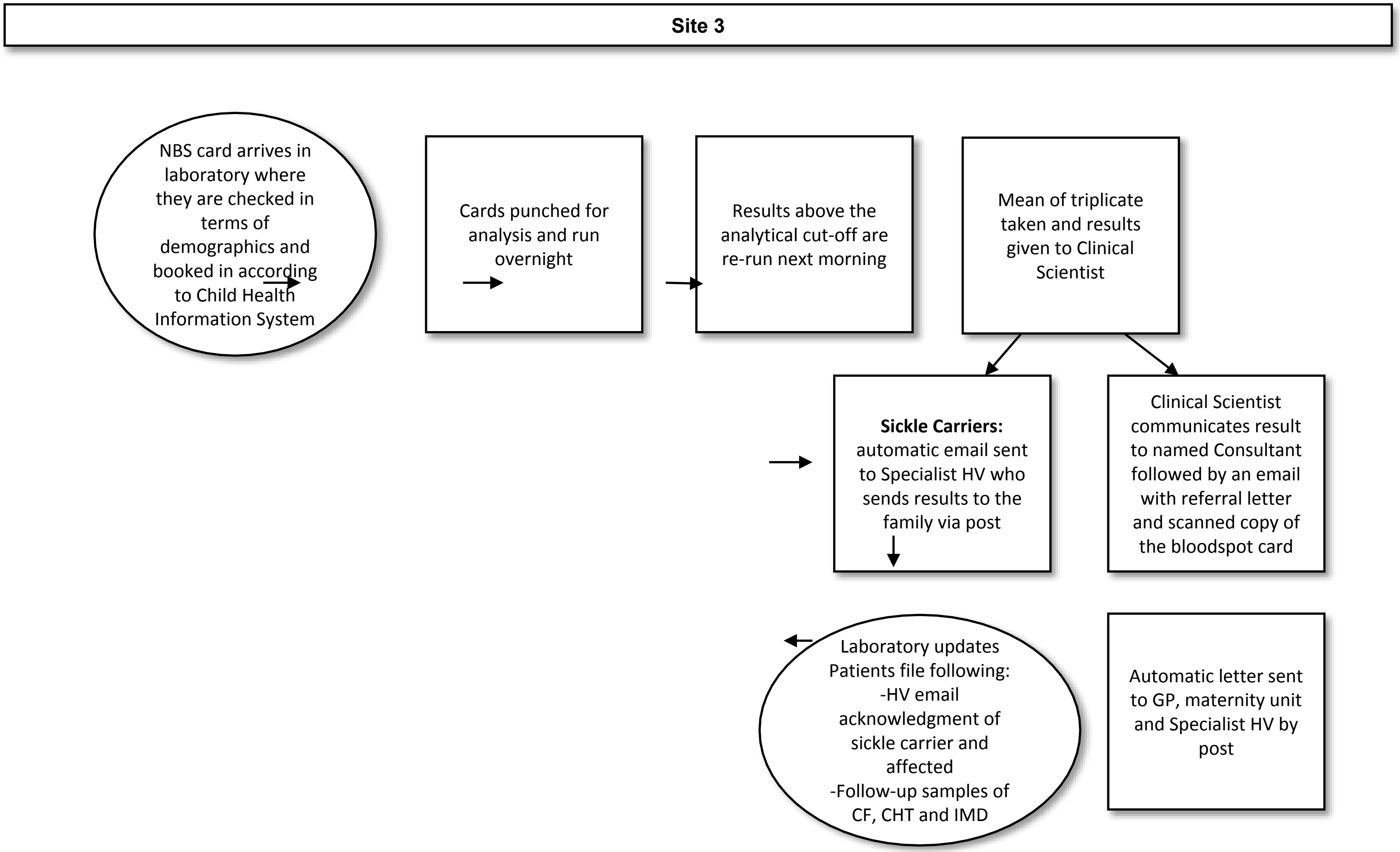
Site 3
NBS card arrives in laboratory where they are checked in terms of demographics and booked in according to Child Health Information System
Cards punched for analysis and run overnight
Results above the analytical cut-off are re-run next morning
Mean of triplicate taken and results given to Clinical Scientist
Sickle Carriers: automatic email sent to Specialist HV who sends results to the family via post
Clinical Scientist communicates result to named Consultant followed by an email with referral letter and scanned copy of the bloodspot card
Laboratory updates Patients file following: -HV email acknowledgment of sickle carrier and affected -Follow-up samples of CF, CHT and IMD
Automatic letter sent to GP, maternity unit and Specialist HV by post

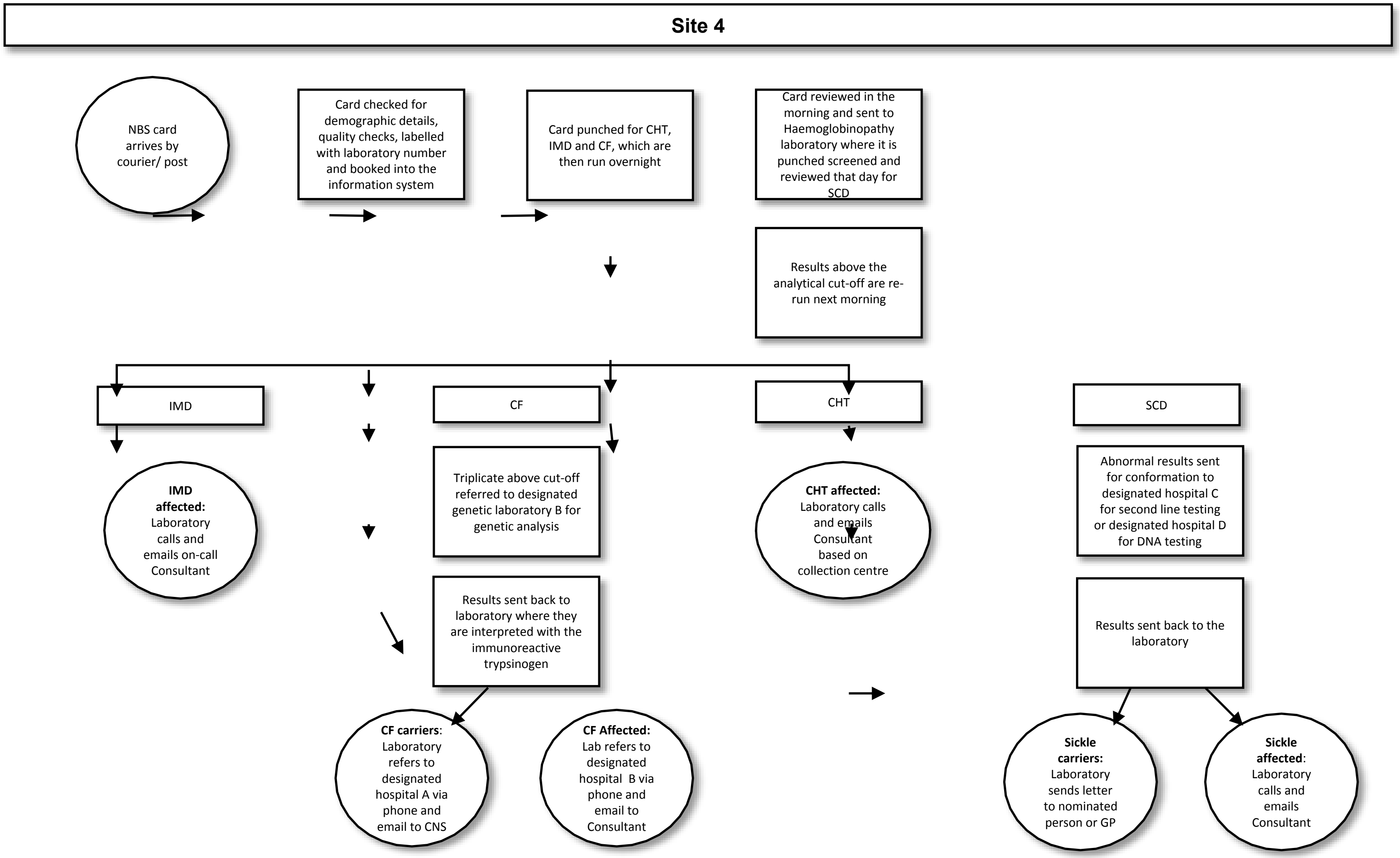
Site 4
NBS card arrives by courier/ post
Card checked for demographic details, quality checks, labelled with laboratory number and booked into the information system
Card punched for CHT, IMD and CF, which are then run overnight
Card reviewed in the morning and sent to Haemoglobinopathy laboratory where it is punched screened and reviewed that day for SCD
Results above the analytical cut-off are re-run next morning
IMD
CF
CHT
SCD
IMD affected: Laboratory calls and emails on-call Consultant
Triplicate above cut-off referred to designated genetic laboratory B for genetic analysis
CHT affected: Laboratory calls and emails Consultant based on collection centre
Abnormal results sent for conformation to designated hospital C for second line testing or designated hospital D for DNA testing
Results sent back to laboratory where they are interpreted with the immunoreactive trypsinogen
Results sent back to the laboratory
CF carriers: Laboratory refers to designated hospital A via phone and email to CNS
CF Affected: Lab refers to designated hospital B via phone and email to Consultant
Sickle carriers: Laboratory sends letter to nominated person or GP
Sickle affected: Laboratory calls and emails Consultant

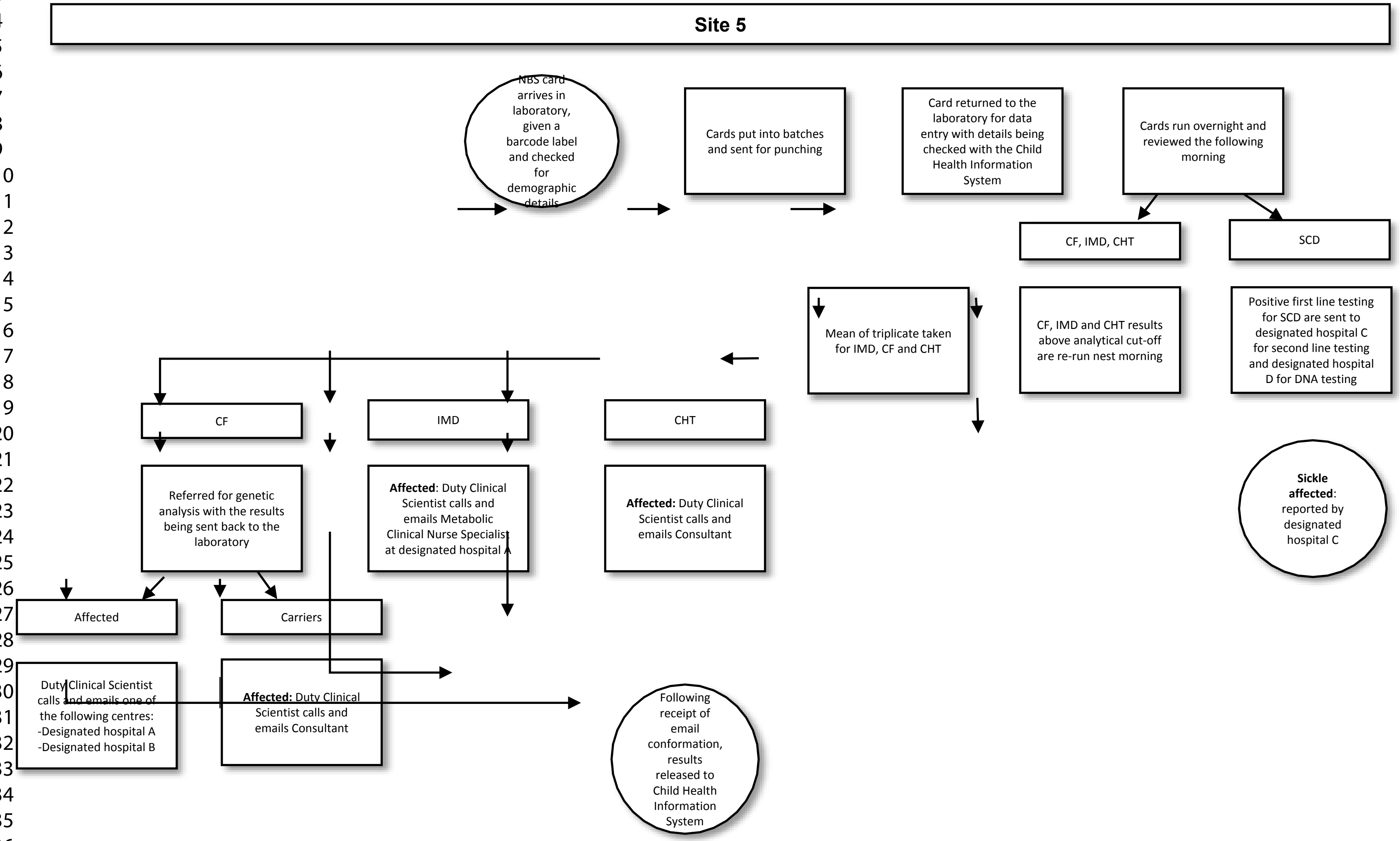
Site 5
NBS card arrives in laboratory, given a barcode label and checked for demographic details
Cards put into batches and sent for punching
Card returned to the laboratory for data entry with details being checked with the Child Health Information System
Cards run overnight and reviewed the following morning
CF, IMD, CHT
SCD
Mean of triplicate taken for IMD, CF and CHT
CF, IMD and CHT results above analytical cut-off are re-run nest morning
Positive first line testing for SCD are sent to designated hospital C for second line testing and designated hospital D for DNA testing
CF
IMD
CHT
Referred for genetic analysis with the results being sent back to the laboratory
Affected: Duty Clinical Scientist calls and emails Metabolic Clinical Nurse Specialist at designated hospital A
Affected: Duty Clinical Scientist calls and emails Consultant
Sickle affected: reported by designated hospital C
Affected
Carriers
Duty Clinical Scientist calls and emails one of the following centres:
-Designated hospital A
-Designated hospital B
Affected: Duty Clinical Scientist calls and emails Consultant
Following receipt of email conformation, results released to Child Health Information System

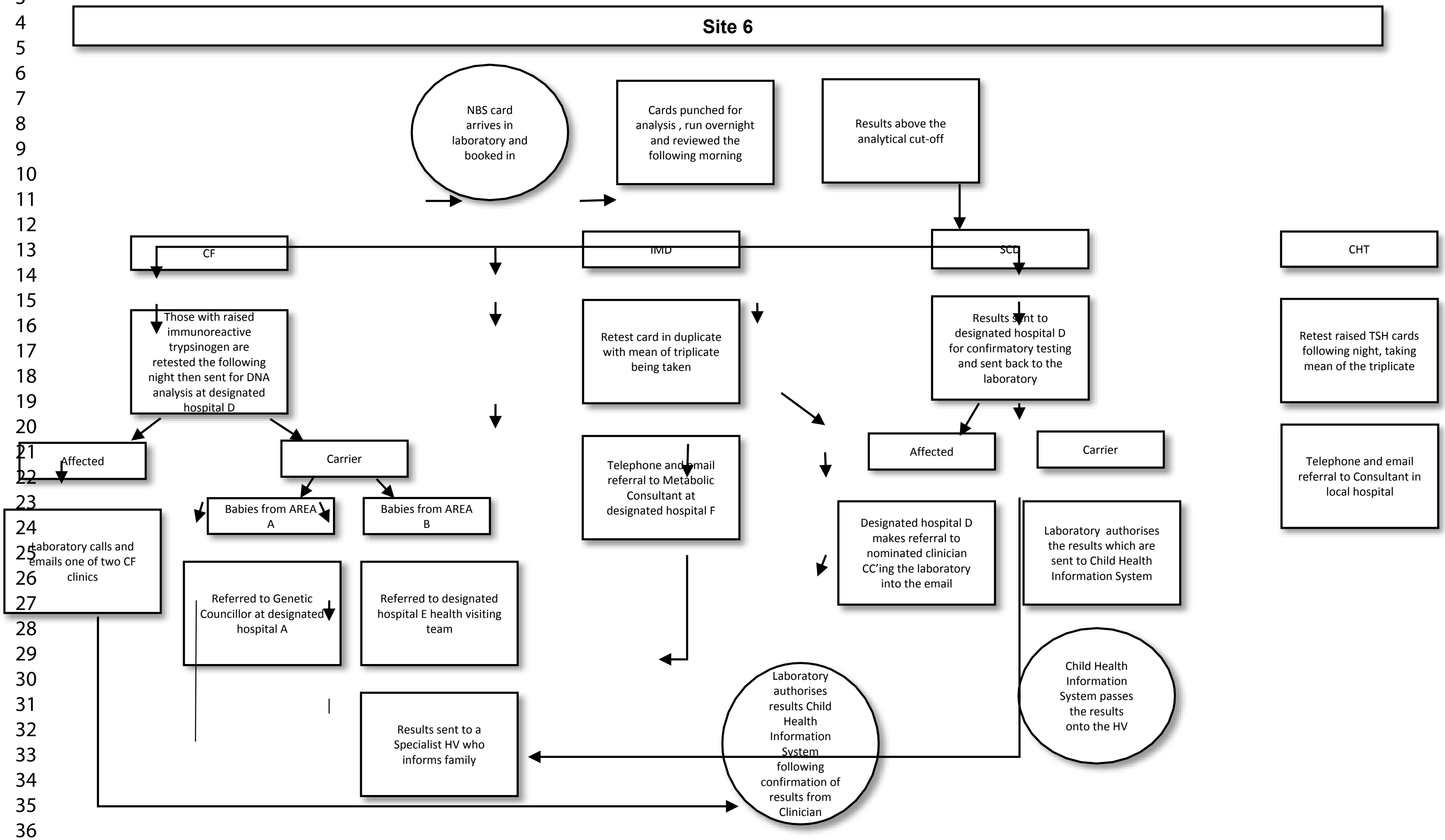
Site 6
NBS card arrives in laboratory and booked in
Cards punched for analysis , run overnight and reviewed the following morning
Results above the analytical cut-off
CF
IMD
SCD
CHT
Those with raised immunoreactive trypsinogen are retested the following night then sent for DNA analysis at designated hospital D
Retest card in duplicate with mean of triplicate being taken
Results sent to designated hospital D for confirmatory testing and sent back to the laboratory
Retest raised TSH cards following night, taking mean of the triplicate
Affected
Carrier
Telephone and email referral to Metabolic Consultant at designated hospital F
Affected
Carrier
Telephone and email referral to Consultant in local hospital
Babies from AREA A
Babies from AREA B
Laboratory calls and emails one of two CF clinics
Designated hospital D makes referral to nominated clinician CC'ing the laboratory into the email
Laboratory authorises the results which are sent to Child Health Information System
Referred to Genetic Councillor at designated hospital A
Referred to designated hospital E health visiting team
Child Health Information System passes the results onto the HV
Results sent to a Specialist HV who informs family
Laboratory authorises results Child Health Information System following confirmation of results from Clinician

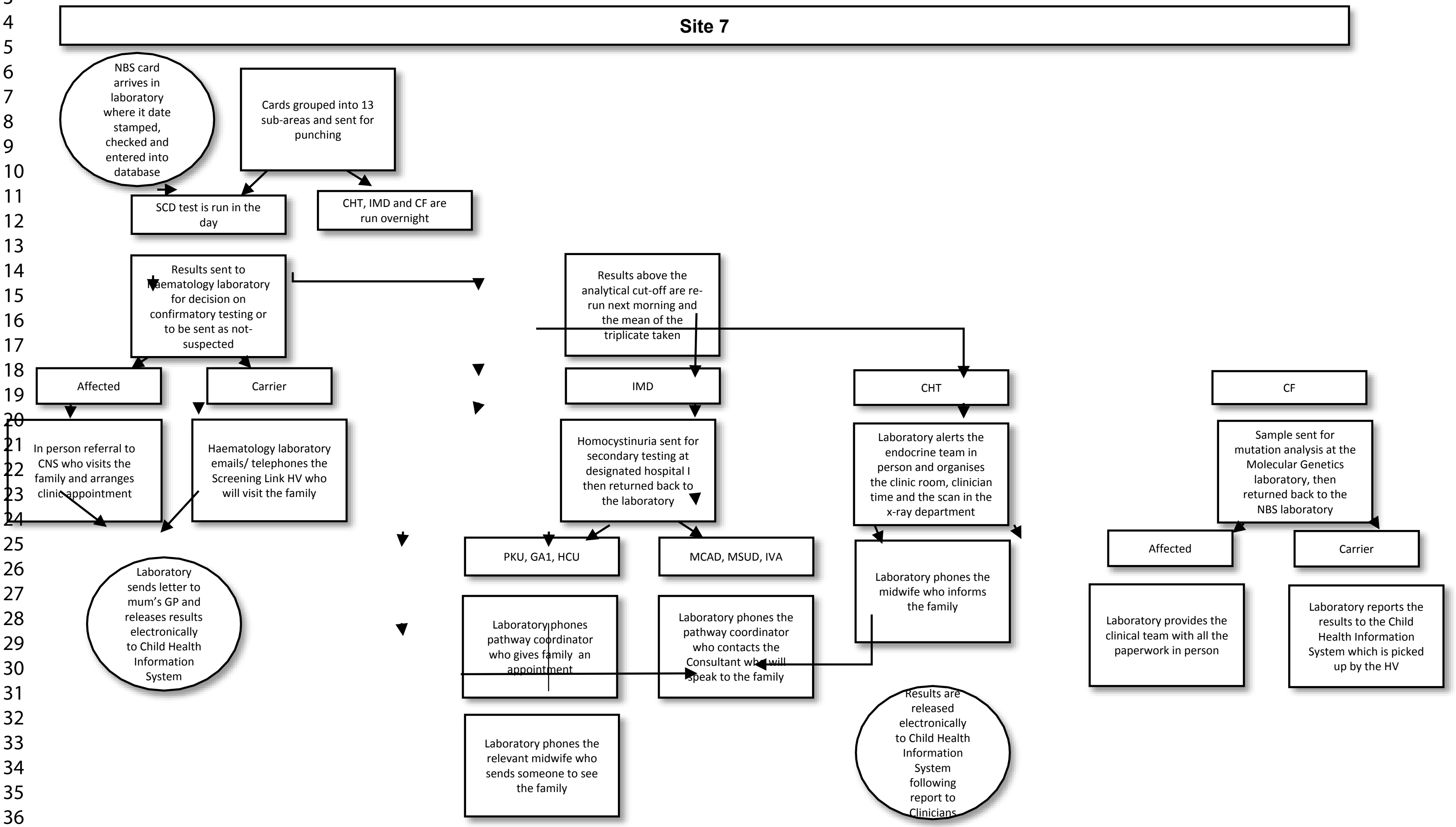
Site 7
NBS card arrives in laboratory where it date stamped, checked and entered into database
Cards grouped into 13 sub-areas and sent for punching
SCD test is run in the day
CHT, IMD and CF are run overnight
Results sent to Haematology laboratory for decision on confirmatory testing or to be sent as not-suspected
Results above the analytical cut-off are re-run next morning and the mean of the triplicate taken
Affected
Carrier
IMD
CHT
CF
In person referral to CNS who visits the family and arranges clinic appointment
Haematology laboratory emails/ telephones the Screening Link HV who will visit the family
Homocystinuria sent for secondary testing at designated hospital I then returned back to the laboratory
Laboratory alerts the endocrine team in person and organises the clinic room, clinician time and the scan in the x-ray department
Sample sent for mutation analysis at the Molecular Genetics laboratory, then returned back to the NBS laboratory
PKU, GA1, HCU
MCAD, MSUD, IVA
Laboratory phones the midwife who informs the family
Affected
Carrier
Laboratory sends letter to mum's GP and releases results electronically to Child Health Information System
Laboratory phones pathway coordinator who gives family an appointment
Laboratory phones the pathway coordinator who contacts the Consultant who will speak to the family
Laboratory provides the clinical team with all the paperwork in person
Laboratory reports the results to the Child Health Information System which is picked up by the HV
Laboratory phones the relevant midwife who sends someone to see the family
Results are released electronically to Child Health Information System following report to Clinicians

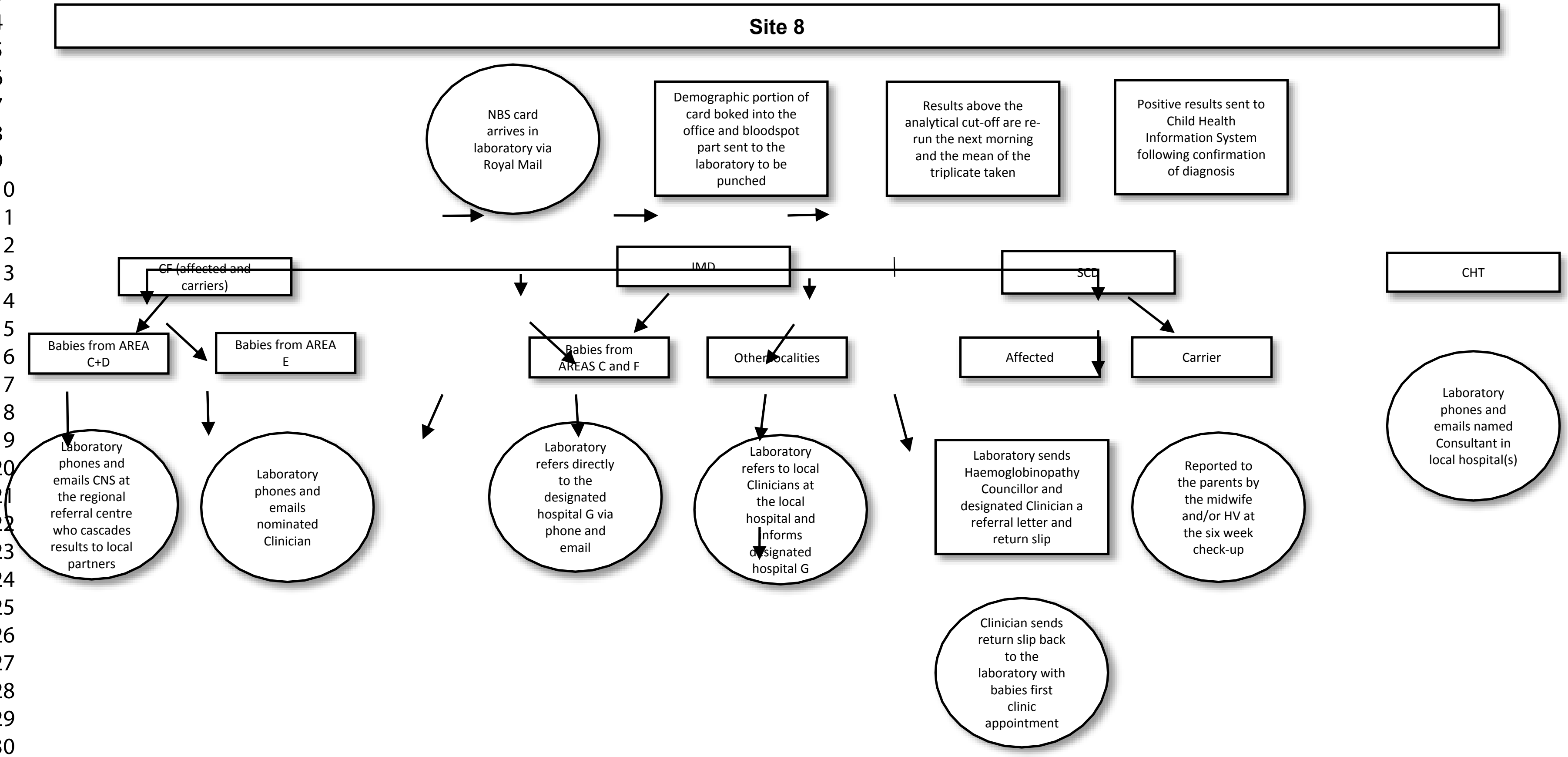
Site 8
NBS card arrives in laboratory via Royal Mail
Demographic portion of card boked into the office and bloodspot part sent to the laboratory to be punched
Results above the analytical cut-off are re-run the next morning and the mean of the triplicate taken
Positive results sent to Child Health Information System following confirmation of diagnosis
CF (affected and carriers)
IMD
SCD
CHT
Babies from AREA C+D
Babies from AREA E
Babies from AREAS C and F
Other localities
Affected
Carrier
Laboratory phones and emails CNS at the regional referral centre who cascades results to local partners
Laboratory phones and emails nominated Clinician
Laboratory refers directly to the designated hospital G via phone and email
Laboratory refers to local Clinicians at the local hospital and informs designated hospital G
Laboratory sends Haemoglobinopathy Councillor and designated Clinician a referral letter and return slip
Reported to the parents by the midwife and/or HV at the six week check-up
Laboratory phones and emails named Consultant in local hospital(s)
Clinician sends return slip back to the laboratory with babies first clinic appointment

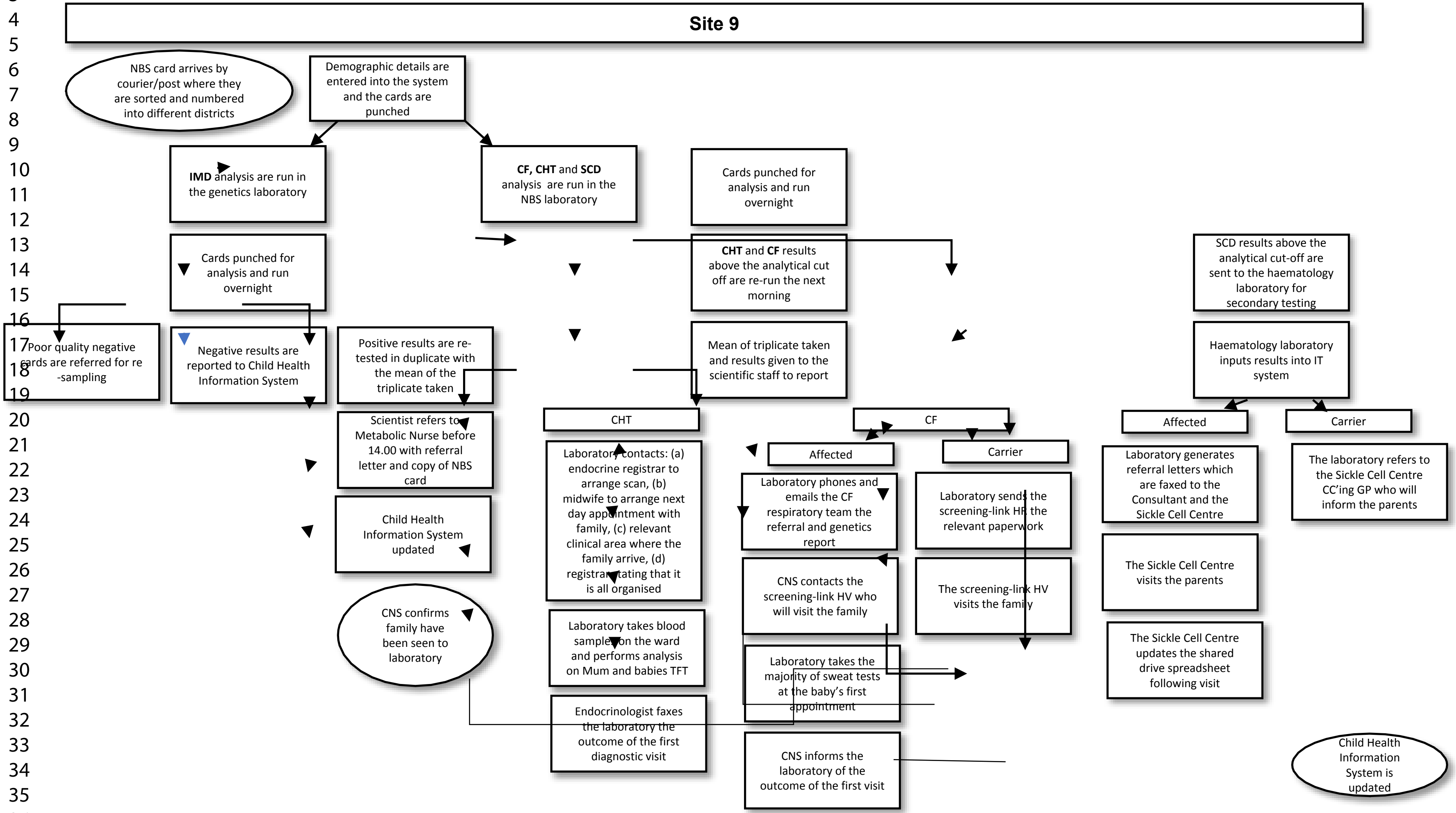
Site 9
NBS card arrives by courier/post where they are sorted and numbered into different districts
Demographic details are entered into the system and the cards are punched
IMD analysis are run in the genetics laboratory
CF, CHT and SCD analysis are run in the NBS laboratory
Cards punched for analysis and run overnight
Cards punched for analysis and run overnight
CHT and CF results above the analytical cut off are re-run the next morning
SCD results above the analytical cut-off are sent to the haematology laboratory for secondary testing
Poor quality negative cards are referred for re-sampling
Negative results are reported to Child Health Information System
Positive results are re-tested in duplicate with the mean of the triplicate taken
Mean of triplicate taken and results given to the scientific staff to report
Haematology laboratory inputs results into IT system
Scientist refers to Metabolic Nurse before 14.00 with referral letter and copy of NBS card
CHT
CF
Affected
Carrier
Affected
Carrier
Child Health Information System updated
Laboratory contacts: (a) endocrine registrar to arrange scan, (b) midwife to arrange next day appointment with family, (c) relevant clinical area where the family arrive, (d) registrar stating that it is all organised
Laboratory phones and emails the CF respiratory team the referral and genetics report
Laboratory sends the screening-link HF the relevant paperwork
Laboratory generates referral letters which are faxed to the Consultant and the Sickle Cell Centre
The laboratory refers to the Sickle Cell Centre CC'ing GP who will inform the parents
CNS confirms family have been seen to laboratory
CNS contacts the screening-link HV who will visit the family
The screening-link HV visits the family
The Sickle Cell Centre visits the parents
Laboratory takes blood sample on the ward and performs analysis on Mum and babies TFT
Laboratory takes the majority of sweat tests at the baby's first appointment
The Sickle Cell Centre updates the shared drive spreadsheet following visit
Endocrinologist faxes the laboratory the outcome of the first diagnostic visit
CNS informs the laboratory of the outcome of the first visit
Child Health Information System is updated

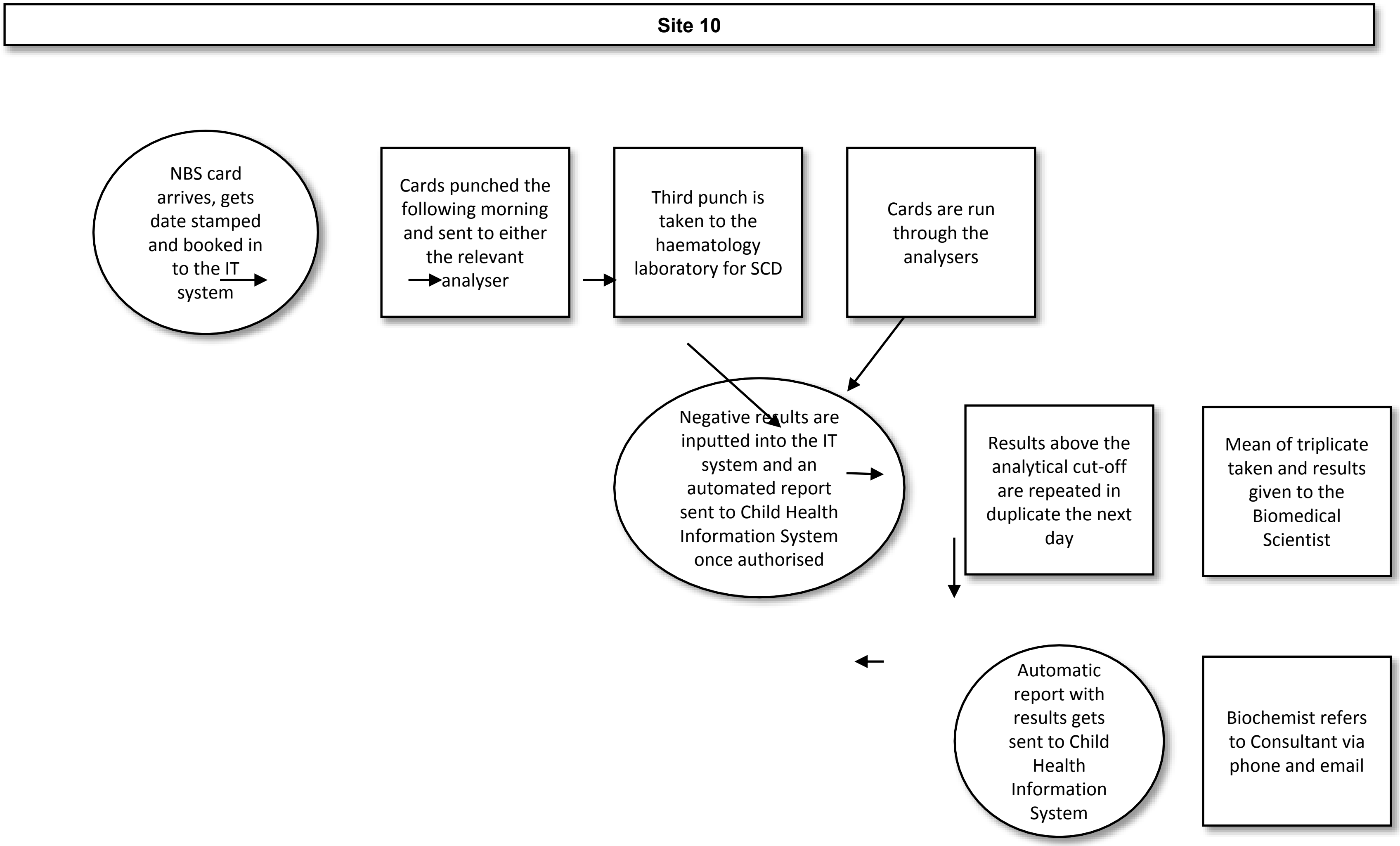
Site 10
NBS card arrives, gets date stamped and booked in to the IT system
Cards punched the following morning and sent to either the relevant analyser
Third punch is taken to the haematology laboratory for SCD
Cards are run through the analysers
Negative results are inputted into the IT system and an automated report sent to Child Health Information System once authorised
Results above the analytical cut-off are repeated in duplicate the next day
Mean of triplicate taken and results given to the Biomedical Scientist
Automatic report with results gets sent to Child Health Information System
Biochemist refers to Consultant via phone and email

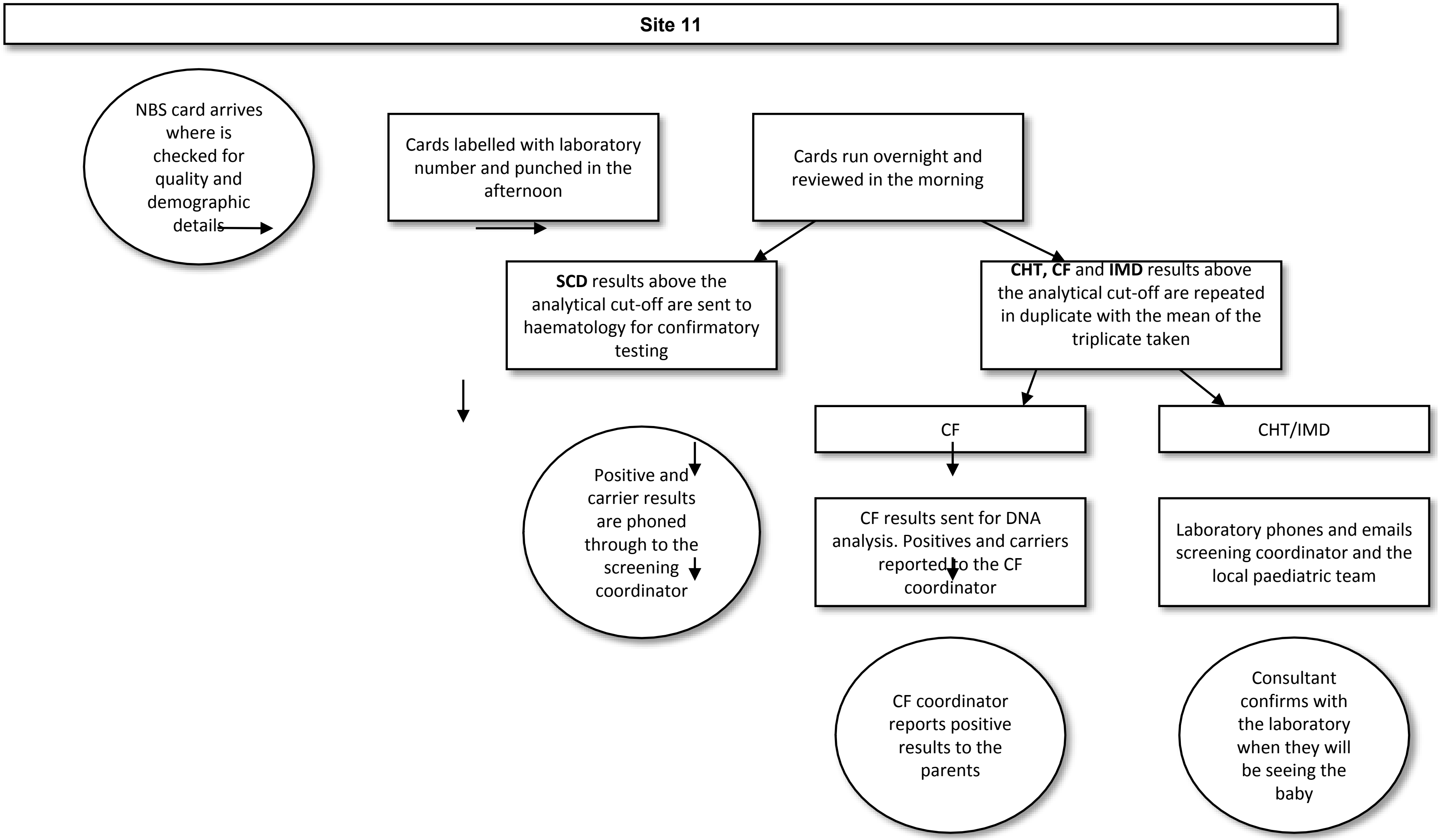
Site 11
NBS card arrives where is checked for quality and demographic details
Cards labelled with laboratory number and punched in the afternoon
Cards run overnight and reviewed in the morning
SCD results above the analytical cut-off are sent to haematology for confirmatory testing
CHT, CF and IMD results above the analytical cut-off are repeated in duplicate with the mean of the triplicate taken
CF
CHT/IMD
Positive and carrier results are phoned through to the screening coordinator
CF results sent for DNA analysis. Positives and carriers reported to the CF coordinator
Laboratory phones and emails screening coordinator and the local paediatric team
CF coordinator reports positive results to the parents
Consultant confirms with the laboratory when they will be seeing the baby

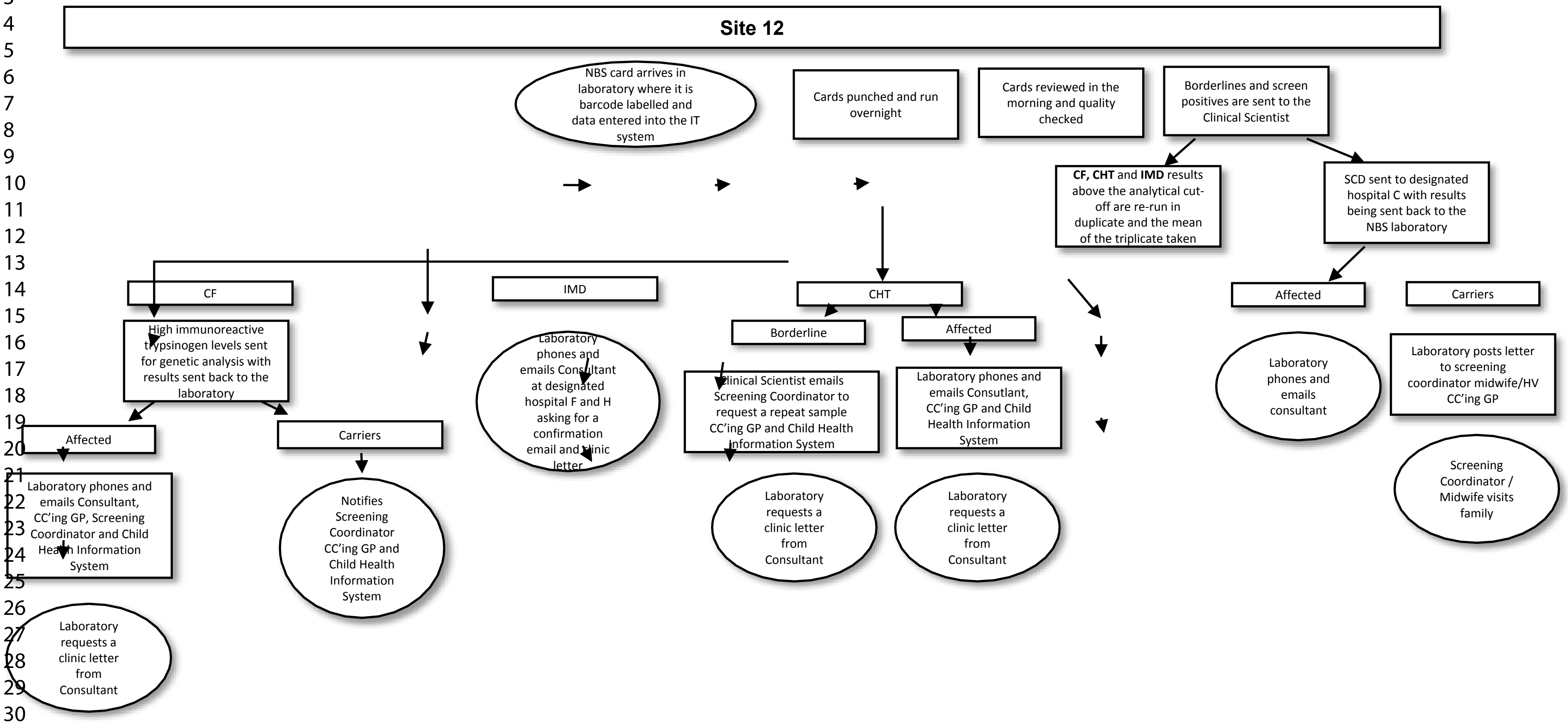
Site 12
NBS card arrives in laboratory where it is barcode labelled and data entered into the IT system
Cards punched and run overnight
Cards reviewed in the morning and quality checked
Borderlines and screen positives are sent to the Clinical Scientist
CF, CHT and IMD results above the analytical cut-off are re-run in duplicate and the mean of the triplicate taken
SCD sent to designated hospital C with results being sent back to the NBS laboratory
CF
IMD
CHT
Affected
Carriers
High immunoreactive trypsinogen levels sent for genetic analysis with results sent back to the laboratory
Laboratory phones and emails Consultant at designated hospital F and H asking for a confirmation email and clinic letter
Borderline
Affected
Laboratory phones and emails consultant
Laboratory posts letter to screening coordinator midwife/HV CC'ing GP
Affected
Carriers
Clinical Scientist emails Screening Coordinator to request a repeat sample CC'ing GP and Child Health Information System
Laboratory phones and emails Consutlant, CC'ing GP and Child Health Information System
Screening Coordinator / Midwife visits family
Laboratory phones and emails Consultant, CC'ing GP, Screening Coordinator and Child Health Information System
Notifies Screening Coordinator CC'ing GP and Child Health Information System
Laboratory requests a clinic letter from Consultant
Laboratory requests a clinic letter from Consultant
Laboratory requests a clinic letter from Consultant

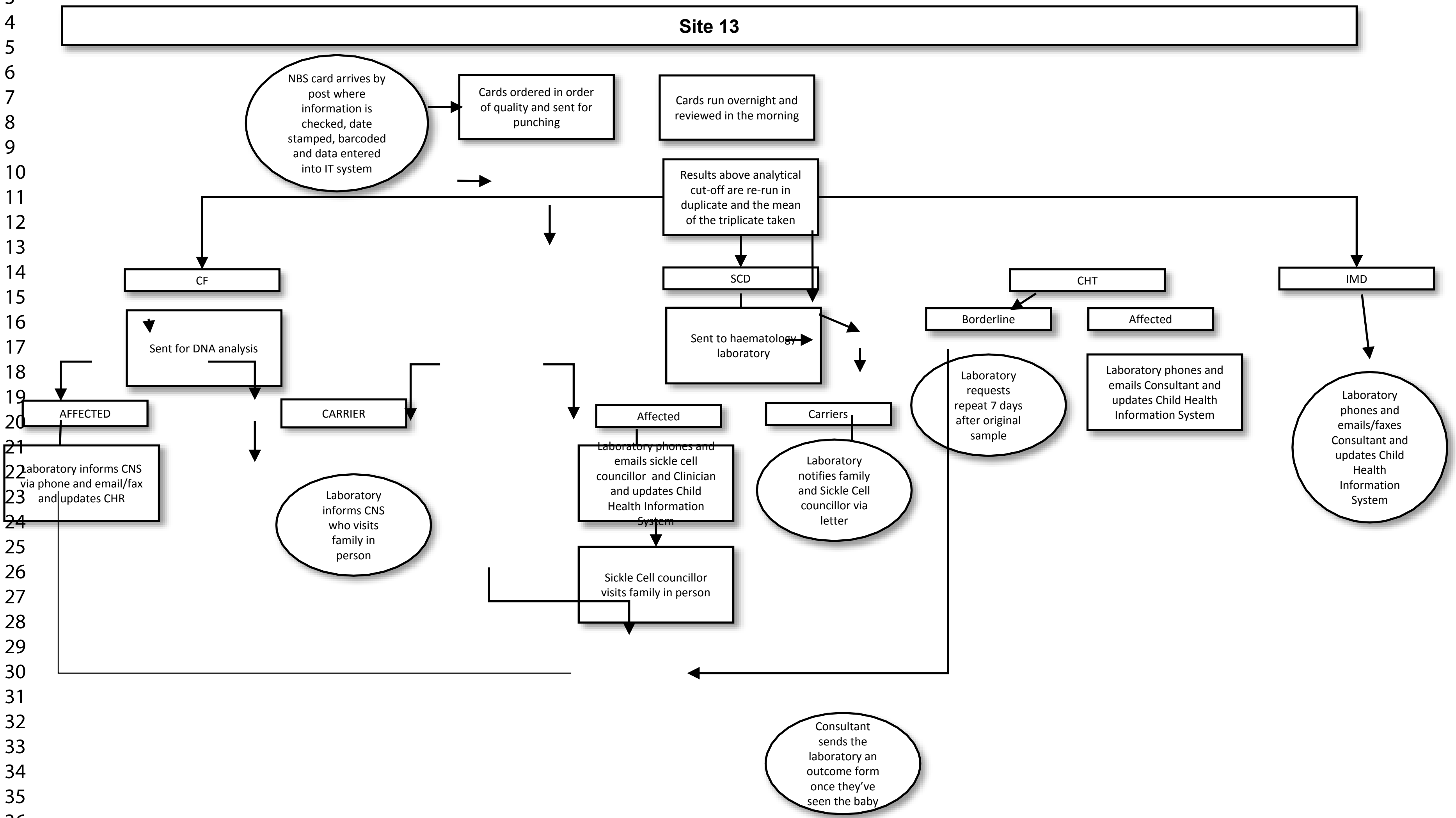
Site 13
NBS card arrives by post where information is checked, date stamped, barcoded and data entered into IT system
Cards ordered in order of quality and sent for punching
Cards run overnight and reviewed in the morning
Results above analytical cut-off are re-run in duplicate and the mean of the triplicate taken
CF
SCD
CHT
IMD
Sent for DNA analysis
Sent to haematology laboratory
Borderline
Affected
AFFECTED
CARRIER
Affected
Carriers
Laboratory requests repeat 7 days after original sample
Laboratory phones and emails Consultant and updates Child Health Information System
Laboratory phones and emails/faxes Consultant and updates Child Health Information System
Laboratory informs CNS via phone and email/fax and updates CHR
Laboratory informs CNS who visits family in person
Laboratory phones and emails sickle cell councillor and Clinician and updates Child Health Information System
Laboratory notifies family and Sickle Cell councillor via letter
Sickle Cell councillor visits family in person
Consultant sends the laboratory an outcome form once they've seen the baby

Consolidated criteria for reporting qualitative studies (COREQ): 32-item checklist

Developed from:
Tong A, Sainsbury P, Craig J. Consolidated criteria for reporting qualitative research (COREQ): a 32-item checklist for interviews and focus groups. *International Journal for Quality in Health Care*. 2007. Volume 19, Number 6: pp. 349 – 357

YOU MUST PROVIDE A RESPONSE FOR ALL ITEMS. ENTER N/A IF NOT APPLICABLE

**No. Item**	**Guide questions/description**	**Reported on Page #**
**Domain 1: Research team and reflexivity**		
Personal Characteristics		
1. Inter viewer/facilitator	Which author/s conducted the inter view or focus group?	Data Collection section
2. Credentials	What were the researcher's credentials? E.g. PhD, MD	PhD
3. Occupation	What was their occupation at the time of the study?	Children's Nurse (JC) and
4. Gender	Was the researcher male or female?	Female
5. Experience and training	What experience or training did the researcher have?	MSc Research Methods; extensive clinical experience; interview techniques.
Relationship with participants		
6. Relationship established	Was a relationship established prior to study commencement?	No
7. Participant knowledge of the interviewer	What did the participants know about the researcher? e.g. personal goals, reasons for doing the research	Professional role – Children's nurse, researcher role in this study
8. Interviewer characteristics	What characteristics were reported about the inter viewer/facilitator? e.g. Bias, assumptions, reasons and interests in the research topic	Positionality and reflexivity section
**Domain 2: study design**		
Theoretical framework		
9. Methodological orientation and Theory	What methodological orientation was stated to underpin the study? e.g. grounded theory, discourse analysis, ethnography, phenomenology, content analysis	Qualitative exploratory design
Participant selection		

For peer review only

10. Sampling	How were participants selected? e.g. purposive, convenience, consecutive, snowball	Purposive followed by snowball
11. Method of approach	How were participants approached? e.g. face-to-face, telephone, mail, email	Email
12. Sample size	How many participants were in the study?	Seventy-one
13. Non-participation	How many people refused to participate or dropped out? Reasons?	Four declined– time constraints / workload demands
Setting		
14. Setting of data collection	Where was the data collected? e.g. home, clinic, workplace	Workplace
15. Presence of non-participants	Was anyone else present besides the participants and researchers?	No
16. Description of sample	What are the important characteristics of the sample? e.g. demographic data, date	Staff involved in communicating the initial positive NBS result in the preceding 6 months.
Data collection		
17. Interview guide	Were questions, prompts, guides provided by the authors? Was it pilot tested?	Yes
18. Repeat interviews	Were repeat inter views carried out? If yes, how many?	N/A
19. Audio/visual recording	Did the research use audio or visual recording to collect the data?	Audio recording
20. Field notes	Were field notes made during and/or after the inter view or focus group?	Yes
21. Duration	What was the duration of the inter views or focus group?	10.40-54.58 mins
22. Data saturation	Was data saturation discussed?	Yes
23. Transcripts returned	Were transcripts returned to participants for comment and/or correction?	No but a staff meeting was held after all interviews had been conducted as per the protocol
**Domain 3: analysis and findings**		
Data analysis		
24. Number of data coders	How many data coders coded the data?	Two
25. Description of the coding tree	Did authors provide a description of the coding tree?	Yes
26. Derivation of themes	Were themes identified in advance or derived from the data?	Derived from the data
27. Software	What software, if applicable, was used to manage the data?	N/A
28. Participant checking	Did participants provide feedback on the findings?	Yes

For peer review only

Reporting		
29. Quotations presented	Were participant quotations presented to illustrate the themes/findings? Was each quotation identified? e.g. participant number	Results section
30. Data and findings consistent	Was there consistency between the data presented and the findings?	Yes
31. Clarity of major themes	Were major themes clearly presented in the findings?	Results
32. Clarity of minor themes	Is there a description of diverse cases or discussion of minor themes?	Discussion

Once you have completed this checklist, please save a copy and upload it as part of your submission. When requested to do so as part of the upload process, please select the file type: *Checklist*. You will NOT be able to proceed with submission unless the checklist has been uploaded. Please DO NOT include this checklist as part of the main manuscript document. It must be uploaded as a separate file.

For peer review only

BMJ Open

Processing of positive newborn screening results: a qualitative exploration of current practice in England

Journal:	*BMJ Open*
Manuscript ID	bmjopen-2020-044755.R1
Article Type:	Original research
Date Submitted by the Author:	04-Nov-2020
Complete List of Authors:	Chudleigh, Jane; City University, School of Health Sciences<br>Chinnery, Holly; St Mary's University Twickenham, Psychology and Pedagogic Sciences<br>Holder, Pru; City University, School of Health Sciences<br>Carling, Rachel; Guy's and St Thomas' NHS Foundation Trust, Biochemical Sciences, Viapath; King's College London, GKT School of Medical Education<br>Southern, Kevin; Institute of Child Health, Paediatrics<br>Olander, Ellinor; City University, School of Health Sciences<br>Moody, Louise; Coventry University<br>Morris, Stephen; University of Cambridge, Primary Care Unit<br>Ulph, Fiona; University of Manchester<br>Bryon, Mandy; Great Ormond Street Hospital For Children NHS Foundation Trust<br>Simpson, Alan; King's College London, Health Services and Population Research; King's College London
**Primary Subject Heading**:	Paediatrics
Secondary Subject Heading:	Genetics and genomics, Health services research, Qualitative research
Keywords:	GENETICS, PAEDIATRICS, Organisation of health services < HEALTH SERVICES ADMINISTRATION & MANAGEMENT

SCHOLARONE™
Manuscripts

I, the Submitting Author has the right to grant and does grant on behalf of all authors of the Work (as defined in the below author licence), an exclusive licence and/or a non-exclusive licence for contributions from authors who are: i) UK Crown employees; ii) where BMJ has agreed a CC-BY licence shall apply, and/or iii) in accordance with the terms applicable for US Federal Government officers or employees acting as part of their official duties; on a worldwide, perpetual, irrevocable, royalty-free basis to BMJ Publishing Group Ltd ("BMJ") its licensees and where the relevant Journal is co-owned by BMJ to the co-owners of the Journal, to publish the Work in this journal and any other BMJ products and to exploit all rights, as set out in our licence.

The Submitting Author accepts and understands that any supply made under these terms is made by BMJ to the Submitting Author unless you are acting as an employee on behalf of your employer or a postgraduate student of an affiliated institution which is paying any applicable article publishing charge ("APC") for Open Access articles. Where the Submitting Author wishes to make the Work available on an Open Access basis (and intends to pay the relevant APC), the terms of reuse of such Open Access shall be governed by a Creative Commons licence – details of these licences and which Creative Commons licence will apply to this Work are set out in our licence referred to above.

Other than as permitted in any relevant BMJ Author's Self Archiving Policies, I confirm this Work has not been accepted for publication elsewhere, is not being considered for publication elsewhere and does not duplicate material already published. I confirm all authors consent to publication of this Work and authorise the granting of this licence.

For peer review only

TITLE: Processing of positive newborn screening results: a qualitative exploration of current practice in England

Jane Chudleigh[1]*, Holly Chinnery[2], Pru Holder[1], Rachel S Carling[3], Kevin W Southern[4], Ellinor K Olander[1], Louise Moody[5], Stephen Morris[6], Fiona Ulph[7], Mandy Bryon[8], Alan Simpson[9]

*corresponding author

[1]Centre for Maternal and Child Health Research, City, University of London, EC1V 0HB, UK; [2]Faculty of Sports, Health and Applied Science, St Mary's University Twickenham, TW1 4SX; [3] Biochemical Sciences, Viapath, Guys & St Thomas' NHS Foundation Trust, London, SE1 7EH and GKT School of Medical Education, King's College London, London, WC2R 2LS; [4]Women's and Children's Health, University of Liverpool, L69 3BX, UK; [5]Centre for Arts, Memory and Communities, Coventry University, CV1 5FB, UK; [6]University of Cambridge School of Clinical Medicine, CB2 0SP, UK; [7]Division of Psychology and Mental Health, The University of Manchester, M13 9PL, UK, [8]Paediatric Psychology and Play Services, Great Ormond Street Hospital for Children NHS Foundation Trust, WC1N 3JH, UK, [9]Florence Nightingale Faculty of Nursing, Midwifery & Palliative Care, King's College London SE1 8WA, UK;

j.chudleigh@city.ac.uk; holly.chinnery@stmarys.ac.uk; pru.holder@city.ac.uk; Rachel.Carling@viapath.co.uk; kwsouth@liv.ac.uk; Ellinor.Olander@city.ac.uk; l.moody@coventry.ac.uk; sm2428@medschl.cam.ac.uk; fiona.ulph@manchester.ac.uk; mandy.bryon@gosh.nhs.uk; alan.simpson@kcl.ac.uk;

For peer review only

ABSTRACT

Objective: To explore current communication practices for positive newborn screening results from the Newborn Bloodspot Screening (NBS) laboratory to clinicians to highlight differences, understand how the pathways are implemented in practice, identify barriers and facilitators and make recommendations for future practice and research.

Design: A qualitative exploratory design was employed using semi-structured interviews

Setting: Thirteen NBS laboratories in England

Participants: Seventy-one clinicians; 22 NBS laboratory staff across 13 laboratories and 49 members of relevant clinical teams were interviewed.

Results: Assurance of quality and consistency was a priority for all NBS laboratories. Findings indicated variation in approaches to communicating positive newborn bloodspot screening results from laboratories to clinical teams. This was particularly evident for congenital hypothyroidism and was largely influenced by local arrangements, resources and the fact individual laboratories had detailed standard operating procedures for how they work. Obtaining feedback from clinical teams to the laboratory after the child had been seen could be challenging and time-consuming for those involved. Pathways for communicating carrier results for cystic fibrosis and sickle cell disease could be ambiguous and inconsistent which in turn could hamper the laboratories efforts to obtain timely feedback regarding whether or not the result had been communicated to the family. Communication pathways for positive NBS results between laboratories and clinical teams could therefore be time consuming and resource intensive.

Conclusion: The importance placed on ensuring positive NBS results were communicated effectively and in a timely fashion from the laboratory to the clinical team was evident from all participants. However, variation existed in terms of the processes used to report positive NBS results to clinical teams and the people involved. Variant practice identified may reflect local needs, but more often reflected local resources and a more consistent "best practice" approach is required, not just in the UK but perhaps globally.

Trial registration: ISRCTN 15330120

Key words: Newborn bloodspot screening, communication

Word count: 4595

For peer review only - http://bmjopen.bmj.com/site/about/guidelines.xhtml

Strengths and limitations of this study

- This is the first known study that has explored communication pathways for positive NBS results from the laboratory to clinical teams.
- Participants represented the 13 NBS laboratories in England involved in managing the nine conditions currently included in the NBS programme increasing the transferability of the findings.
- The study design, data collection and analysis were influenced by members of the PPI advisory group and relevant charities.
- The researchers are experienced in this field which may have biased data collection and analysis.

For peer review only

INTRODUCTION

In the UK, Newborn Screening (NBS) comprises a heel prick blood sample taken via a NBS card between days 5-8 of life to identify pre-symptomatic babies that are affected by genetic or congenital conditions[1]. It is known that early diagnosis leads to better health outcomes for the child[1 2]. In the UK, since January 2015, NBS covers nine conditions (See Table 1).

Table 1 Conditions included in the NBS Programme

Name of Condition	Abbreviation
Sickle Cell Disorder	SCD
Cystic Fibrosis	CF
Phenylketonuria	PKU
Homocystinuria	HCU
Glutaric aciduria type 1	GA1
Medium chain acyl CoA dehydrogenase deficiency	MCADD
Maple syrup urine disease	MSUD
Isovaleric aciduria	IVA
Congenital Hypothyroidism	CHT

Each year, around 10,000 babies born in the United Kingdom (UK) have a positive NBS result. These positive NBS results include both babies who will eventually be diagnosed as being affected by one of the nine life changing conditions currently screened for[3 4] (n= approximately 1500), those who will later be confirmed as gene carriers for Sickle Cell Disorder (SCD) or Cystic Fibrosis (CF) but unaffected by the disease and babies who screen positive but for whom screened disease is ruled out (false positive results).

Communicating Presumptive Positive NBS results

NBS Laboratory guides are available for each of the conditions included in the NBS Programme in the UK[5-9]. Positive NBS results are referred to as being 'presumptive positive'

For peer review only - http://bmjopen.bmj.com/site/about/guidelines.xhtml

For peer review only

(PP) results. That is, the result is presumed to indicate a positive result until further diagnostic testing is undertaken to either confirm or rule out the condition being screened for. Within each condition, there is specific information on how PP cases should be reported and communicated. While some aspects of the pathways are similar, some are very different such as the time frame for the first clinic appointment. Table 2 summarises the different pathways as established in the guides, for each of the screened conditions.

Communicating positive NBS results is not an event but a process that starts from the moment the result is identified by the NBS laboratory as being above the agreed analytical 'cut off' and ends when the parents are given the definitive diagnosis for their child[10]. Most literature to date has focussed on how positive NBS results are communicated to families via appropriate clinicians[11-16]. These studies have highlighted that despite guidance, this occurs in a range of ways which are not currently well defined. In addition, poor, or inappropriate, strategies for communication of positive NBS result to families can influence parental outcomes in the short term[11 13 16-19] but may also have a longer-term impact on children and families[20].

The NBS laboratory guides[5-9] provide detailed explanations regarding the analytical aspects of NBS, quality assurance, clinical referral and follow up and reporting and data collection for each of the screened conditions. However, no literature has explored how the process of communication from the laboratory to clinical teams is implemented in practice and how this might influence communication with families.

The purpose of the current study was to explore current communication practices for positive newborn screening results from the NBS laboratory to clinicians to highlight differences, understand how the pathways are implemented in practice, identify barriers and facilitators and make recommendations for future practice. Health professionals' experiences of communicating positive newborn bloodspot screening (NBS) results to families have been reported elsewhere[10].

For peer review only - http://bmjopen.bmj.com/site/about/guidelines.xhtml

Table 2: UK guidelines for Communicating positive NBS results from laboratories to clinical trams following a presumptive positive (PP) NBS result

Condition(s)	**Method of referral from laboratory to clinical team**	**Time Frame for communicating a PP result to the clinical team**	**Requirements when communicating PP results to families**	**Time frame for first clinic appointment**
Congenital Hypothyroidism[8]	Verbally and in writing by secure email including a link to the standardised diagnostic and initial treatment protocol, to either a paediatric endocrine team or a lead paediatrician with a special interest in CHT	Same or next working day of the definitive NBS result being available	Communicated to the family by an 'informed health professional' who should provide the family with the appropriate information leaflet available via the screening programme and the child's appointment details	Must take place on the same day or the next day after parents are informed of their baby's positive NBS result.
Cystic Fibrosis[5 9]	Refer the baby immediately to a designated person at the appropriate Regional CF Centre. The regional CF centre will usually be responsible for liaising with local CF clinics	Within one working day of the definitive screening result	Parents should be informed by an 'appropriate health professional' but not on a Friday, Saturday or Sunday and that ideally Thursday should be avoided as well. The family should be contacted via a structured	Parents should be offered an appointment on the following day after they are informed of the PP result and the diagnostic assessment within five working days of receiving the result.

For peer review only - http://bmjopen.bmj.com/site/about/guidelines.xhtml

			telephone call in the first instance informing them that the result will be discussed later that day by a healthcare professional with appropriate experience and support to give bad news. Following this, the guidance states that the positive CF screening result may be given at a home visit but sometimes a second structured phone call might be used. In both instances, it is recommended this should be done by a healthcare professional with knowledge of screening and CF	
Sickle Cell Disease[7]	Must be reported as per local pathways, to the clinician and/or designated sickle cell and thalassaemia centre	Not specified	Local protocols must ensure that PP results are given to parents by a trained healthcare professional face-to-face by 28 days of age	The baby should enter the care of a specialist haemoglobinopathy centre by 90 days of age

For peer review only

For peer review only - http://bmjopen.bmj.com/site/about/guidelines.xhtml

Inherited Metabolic Diseases (IMDs) including (MCADD, MSUD, PKU, IVA, HCU, GA1)[6]	Referral of all IMD PP patients should be undertaken in co-ordination with a clinical service that complies with the service specification for a paediatric IMD centre to ensure continuity of specialist care. PP cases should be reported to the specialist clinical team (or designated local team for PKU) verbally and in writing.	Same working day that the PP result is available	Parents should be informed of the PP NBS result face-to-face, usually at home by a member of the relevant IMD team and provided with the relevant 'suspected leaflet' and details of the first clinic appointment (PKU); provided with the relevant 'suspected leaflet' and details of the first clinic appointment via email (MCADD), by a member of the IMD specialist team (HCU) who should also liaise with the appropriate hospital until the baby can be transferred to the specialist IMD centre (MSUD, IVA, GA1)	The baby should be: reviewed at an appropriate hospital within 24 hours (MCADD) or on the next working day via web link or in person (HCU); or the same or next working day (PKU); or taken to an appropriate hospital (if not an inpatient already) where initial assessment will take place and then transferred to a specialist IMD centre on the same day the PP screening result is available. If transfer is not available, the specialist team will liaise with the local hospital to arrange diagnostic testing and supply of dietary supplements (MSUD, IVA, GA1).

For peer review only

For peer review only - http://bmjopen.bmj.com/site/about/guidelines.xhtml

METHODS

A qualitative exploratory design was utilised using semi-structured telephone interviews with laboratory staff employed in the 13 NBS laboratories in England and members of relevant clinical teams notified of positive NBS results from the respective NBS laboratories. This study is part of an ongoing programme of work[21] and was approved by the London Stanmore ethics committee (17/LO/2102).

Patient and Public Involvement

Patient and public involvement (PPI) was instrumental in the design and conduct of this study. Eight parents of babies who had received a positive NBS screening result for one of the nine screened conditions formed a PPI advisory group who met prior to, during and following data collection. Their suggestions were incorporated into the study design, the data collection tools and the data analysis and presentation. The PPI group were presented with data from the annual reports of the NBS Programmes and made suggestions as to which sites should be used in later Phases of the study design. The PPI group also suggested clarifying that positive NBS results indicated an 'abnormal' result while negative NBS results indicated a 'normal' result during data collection, as they had found this confusing when they received their child's NBS result. Initial findings of were presented to member of the PPI group during regular 6 monthly meetings and drafts of manuscripts were also shared with PPI members to ensure these were presented in a readable format. In addition, we obtained the views of representatives from charities for the screened conditions including Metabolic Support UK, the British Thyroid Foundation, the CF Trust and the Sickle Cell Society.

Setting

In England, there are 13 Newborn Screening laboratories that process the results for the nine conditions that are currently included in the NBS Programmes, these comprised the study sites.

Inclusion and Exclusion Criteria

Staff employed in NBS laboratories and involved in the processing of positive NBS results and members of relevant clinical teams who had received a positive NBS from NBS laboratories in the previous 6 months were included. Staff who had not been involved in processing or receiving positive NBS results in the last 6 months or who had personal experience of receiving a positive NBS result were excluded.

For peer review only - http://bmjopen.bmj.com/site/about/guidelines.xhtml

Recruitment and sampling

A two-stage sampling approach was employed where participants were first sampled purposively based on their experience with the phenomena of interest, followed by a second stage of snowball sampling where the first participants suggested others. Directors of all 13 NBS-laboratories in England were invited to participate. These were identified through the UK Newborn Screening Laboratories Network (http://www.newbornscreening.org/site/laboratory-directory.asp) and were contacted via email by a member of the research team. Directors of newborn screening laboratories were invited to be the Local Principal Investigator for their study site and were asked to provide names and contact details of staff within the laboratory who met the inclusion criteria for the study. These staff members were contacted via email and invited to participate. Representative members of local clinical teams (medical consultants; general paediatricians; nurse specialists; health visitors; specialist screening nurses, genetic counsellors) were identified through individual trust websites and contacted via email to be invited to participate. Those who agreed to participate were also asked to identify other members of their team who they thought should be interviewed to provide further information about the NBS process. These additional potential participants were also contacted by email and invited to participate. Written informed consent was obtained from all participants.

Data Collection

Semi-structured telephone interviews comprising closed and open-ended questions were conducted by JC between June 2018 and February 2019 to identify the approaches used to communicate positive NBS results from NBS laboratories to health professionals (Supplementary File A). Data were collected on: the mode of communication strategy (face-to-face; letter; telephone; e-mail); the resources involved in each communication strategy; who provides the information and their role; location (co-located or alternative site) of relevant services for each condition.

Data Analysis

The purpose of data analysis was to describe and identify approaches currently used to communicate positive NBS results from NBS laboratories to health professionals. Qualitative data from open-ended questions were analysed using thematic analysis[22] with an inductive

For peer review only - http://bmjopen.bmj.com/site/about/guidelines.xhtml

approach. Data from laboratory staff and clinical staff were analysed separately. Seven interview transcripts from laboratory staff were coded by two members of the research team (JC and HC) in order to aid coding comparisons and inform and align code development[23]. A code book was developed based on these jointly coded transcripts. A further seven laboratory transcripts were then coded separately by the same two members of the research team using the code book. These separately coded transcripts were compared, a similar process was followed for the transcripts for clinical staff. Following this, the same two members of the research team coded the remainder of the laboratory and clinical staff transcripts using the relevant code books. This was an ongoing, iterative process; new codes were developed and the definition of codes refined as analysis progressed.[24] Once this initial coding had been completed, these codes were then collapsed into themes. Communication pathways for each condition for each of the 13 NBS laboratories (unit of analysis) were also developed (Supplementary file B).

Positionality and reflexivity

Members of the study team (JC, LM, FU, MB, KWS) have been involved in or continue to undertake a variety of roles and activities associated with the NBS Programme in the UK. It is acknowledged that this could have led to potential bias during data collection and analysis. However, this was balanced by other members of the research team who had previously had minimal involvement in NBS (HC, EKO, AS, SM, PH). Data collection was undertaken by JC and data analysis was mainly undertaken by JC and HC who fall within both camps. Neither JC nor HC were employed in the organisations where data collection was undertaken.

RESULTS

In total, 71 interviews were conducted: 22 with NBS laboratory staff across 13 laboratories and 49 with members of clinical teams. Four eligible participants declined to be interviewed. Demographics of participants can be seen in Table 3.

Table 3: Demographics of participants

NBS laboratory staff	
**Profession**	**Number of staff interviewed**
Director of NBS laboratory	9

For peer review only - http://bmjopen.bmj.com/site/about/guidelines.xhtml

Consultant Biochemist / haematologist		2
Senior/ Clinical Scientist		11
Length of service	Median 12.0 years	Range 1.0-22.0 years
Length of interview	Median 30.8 minutes	Range 13.3-45.1 mins
**Clinical Teams**		
**Profession**		**Number of staff interviewed**
Medical Consultant		21
Clinical Nurse Specialist		21
Screening Specialist Nurse / Midwife		5
Service Coordinator		1
Paediatric Dietician		1
Length of service	Median 11.0 years	Range 1.5-23.0 years
Length of interview	Median 33.4 minutes	Range 10.4-54.6 mins

Themes

Five themes were identified from the data: the importance of the result to the child and family; different referral approaches for different conditions; no unified process to provide feedback to laboratories; providing carrier results; inconsistency and ambiguity and resource use and responsibilities. These are explored in detail below and supported by illustrative quotations from interview data.

The importance of the result to the child and family

Assurance of quality and consistency was a clear priority for all NBS laboratories. This applied to all aspects of analysis including quality checks of the NBS card, data entry, first line and if required, second line testing, and timeliness of reporting. There was also a clear appreciation of the significance of the results for families.

"…everybody is aware there is a baby at the end of this, there's a family at the end of this and everybody wants to do the best by that family. So, I think because of that everybody pulls together really well and makes sure, even if it's bad timing, which it usually is, everybody still pulls together to make sure it happens." Site 13

For peer review only - http://bmjopen.bmj.com/site/about/guidelines.xhtml

In addition, laboratory staff clearly appreciated the urgency of communicating positive NBS results to clinicians particularly for the Inherited Metabolic Diseases (IMDs) including MCADD, MSUD, PKU, IVA, HCU, GA1.

"Any of the metabolic conditions, if we see a raised result for first time, we will repeat that immediately. So, rather than it going through the system and being repeated, and us only getting the second result the next day, we'd repeat it immediately, so we repeat it effectively offline, and then if it turns out to be positive, we would make the referral straight away." Site 8.

Different referral approaches for different conditions

Even though national guidelines are available from Public Health England (PHE) for referring positive NBS results from the laboratory to the relevant clinicians, 10 out of 13 of the laboratories created their own templates for this purpose following further development and improvement by staff:

"... we have a proforma that we send ...when we get the results. I know there's a national one which we've looked at but we think our own one probably ticks more boxes. From our point of view, it seems to work for us better" Site 7

When a positive NBS result occurs, referrals are made to a range of different clinicians including condition specific consultants, their secretaries, condition specific specialist nurses, specialist screening nurses or screening co-ordinators. The referral process was often based on local arrangements, resources and the fact individual laboratories had detailed standard operating procedures for how they work. These could be quite complex with variation for each condition and often the need to cover large geographical areas. However, usually these were well understood and implemented.

"...we've got SOPs [standard operating procedures] that define where we need to call for whichever result in whichever place...for our congenital hypothyroidism, we have named consultants at [Hospital E]. We have a name for [Hospital F] and [Hospitals G, H, I and J] where we'll just report to an on-call paediatrician, So, PKU only if they're [Hospital J] babies we report to a [Hospital J] paediatrician, or there's one of two [Hospital J] paediatricians. If we can't get them, then it goes to the metabolic team at the [Hospital A].

For peer review only - http://bmjopen.bmj.com/site/about/guidelines.xhtml

Then, MCADD babies if they are [Hospital E] or [Hospital J], then we can report them either to a [Hospital E] named clinician or the [Hospital J] named clinicians. Again, if they're not available, then they'll go down to the metabolic team at the [Hospital A]" Site 3

Many factors were seen to promote effective communication between the laboratory and clinical teams. This included teams being small which meant everyone within the team knew one another and understood each other's roles; the proximity of teams within sites made communication easier between laboratory staff and relevant clinical teams; and good working relationships amongst teams within the same hospital and across sites.

"I think the close relationship between all the different professional groups for this part of the country works extremely well. Everybody tends to know each other personally; it means there are no barriers to discussion… Now, increasingly, we're working as closely as we can with [Hospital D] in particular…they'll all cover each other, and the labs work quite closely together. We have joint meetings occasionally and to some extent, we have a joint strategic vision because there are specialist lab things that each one of the three of us do. So, we're trying to develop separate areas and compliment rather than compete." Site 9

However, for many laboratories, referral of positive NBS results for CHT was viewed as more problematic. For all of the other screened conditions, dedicated condition specific specialist clinical teams are available to receive the positive NBS result. However, while some babies who have a positive NBS result for CHT will be seen within specialist endocrine teams, CHT is generally viewed as being possible to manage by general paediatricians and therefore, often babies will be referred to local paediatric services. This can mean that in some instances, there is not a named individual to act as a point of contact.

"I'm having to go through a switchboard at a different hospital to try and find somebody who might not be in that hospital because their clinic's in another hospital… I would love it if I just had one person to call about all my hypothyroidism babies, make my life so much easier if I didn't have to phone different GPs and different consultant endocrinologists." Site 10

Despite these challenges, laboratory staff were acutely aware of the importance of obtaining this feedback to ensure the screening process was complete. Therefore, even though it meant that they often had to invest additional time, laboratory staff would ensure that feedback was

For peer review only - http://bmjopen.bmj.com/site/about/guidelines.xhtml

For peer review only

received so the final outcome (true positive, false positive, carrier) could be recorded accurately and in a timely manner. Concerns were also raised by laboratory staff and members of clinical teams about the equity of care particularly in relation to availability of scans following a positive NBS result for CHT.

"So in terms of equity of care, it would seem that, given that it's a national screening programme, people should be having the same tests for diagnosis as well." Site 4

Conversely, communication of positive CHT NBS results to families was seen as being more straightforward to manage than some of the other conditions included in the NBS programme.

"We do far less visits for CHT babies. They're mainly phoned up and told about the results, and then told when the appointment is and where …It's a lot simpler disorder." Site 1

No unified process to provide feedback to laboratories

In the UK, following the referral of a baby with a positive screening result, NBS laboratories require feedback from the relevant clinical team once the baby has been seen, assessed and confirmatory testing has been undertaken. There was no consistent unified national approach to providing this feedback, which led to time-consuming efforts by the laboratory staff to obtain information. In contrast, the responsible clinicians often felt they had a clear idea of their duties in this regard.

"…if we don't receive feedback, we have to phone and write letter, and that does take quite a bit of time." Site 1

This was in contrast to the views of clinicians who were responsible for providing the feedback to the NBS laboratories who described steps they took to ensure this information was fed back to the laboratories. This suggests that there may be a mismatch between the information the laboratories actually require, the information clinicians are providing and who is seen to have ownership of the information.

For peer review only - http://bmjopen.bmj.com/site/about/guidelines.xhtml

"Then, what I will normally do then is email [the NBS laboratory] back to say, 'Yes, the parents will be attending,' and so on, or, if the parents declined, which has never happened, 'Okay, they're not coming,' and I would assume they would follow up." Site 7

The ability of the laboratory to collate and coordinate feedback from different sources, after a child had been seen, was considered to be particularly challenging and time-consuming for CHT. This was often attributed to the fact that affected babies were often seen in 'local' centres rather than tertiary referral centres due to the treatable nature of the condition. As a result, clinicians from many more localities might be involved in their care. To remedy this, some laboratories had sought local solutions to help them deal with the difficulties associated with feedback for positive NBS results for CHT.

"CHT is much more of a problem in this region because we are not phoning one individual consultant in this region…The CHT is-, I have to chase around a lot more to get that information from other hospitals. They are not very forthcoming, often, at providing the information so I have to chase around, and often I have to get my consultant colleague here, the endocrinologist, to help me with that because that can be a real challenge." Site 10

This was in contrast to the IMDs where the conditions were viewed as being more urgent due to the potential life-threatening nature of particularly MCADD, MSUD and IVA. Also, because affected babies were only seen at tertiary centres where often the NBS laboratory staff worked closely both physically and personally with IMD clinical team members.

"…for the IMD conditions, because the consultant that sees them is based in the same hospital as me [Laboratory Director] and the fact he is my colleague, we work together to provide the IMD clinical service, I do get that information from him. I also can see that the diagnostic blood tests have been received in our laboratory. So, I know those babies have been seen." Site 10

Providing carrier results; inconsistency and ambiguity

Pathways for communicating carrier results to families were viewed by some laboratories as ambiguous and inconsistent. NBS laboratories expressed concern regarding whether parents had been informed of their baby's SCD or CF carrier status and by whom as this information was often difficult for them to ascertain. Carriers are healthy children who carry a single

For peer review only - http://bmjopen.bmj.com/site/about/guidelines.xhtml

faulty gene. The most commonly received positive newborn screening result is for carrier status for SCD. Communication pathways can include a range of health professionals including sickle cell coordinators, sickle cell counsellors, general practitioners, and/or health visitors via telephone, letter, home visit or during the baby's health review when they are six weeks of age. Therefore, although this represents a different result scenario (the baby is considered healthy), there is still evidence of inconsistency in terms of the pathways used.

"... for a child who has who has been found to have a carrier status of sickle cell, we just correspond with the health visitor and the GP in a non-urgent way because that is something that just needs to be added to their record but probably won't impact on their health greatly." Site 2

The purpose of the CF NBS protocol in the UK is to maximise the detection of affected individuals (those with two disease causing mutations of the CF transmembrane regulator (CFTR) gene) while minimising the detection of unaffected carriers of CF. However, when carrier results are identified, these too need to be communicated to families. Similar to SCD, this occurs in a range of ways by different people including general practitioners, health visitors, specialist nurses and genetic counsellors via telephone or home visits.

"CF carriers, we've got two specialist nurses within our screening lab and they actually go and do a home visit with the families and give them the CF carrier result in person" Site 13

Feedback to the NBS laboratories regarding whether the parents had been informed of their baby's SCD or CF carrier status and by whom was also of concern to some laboratories.

Resource use and responsibilities

NBS cards are used to collect blood from infants and information from their parents when the NBS heel prick sample is taken when the child is approximately 5 days of age. Procedures for processing NBS cards for the nine conditions currently included in the NBS Programme were considered to be transparent and efficient; clinicians within the NBS laboratories were able to discuss these in detail and seemed satisfied with how the laboratory guidelines were operationalised.

For peer review only - http://bmjopen.bmj.com/site/about/guidelines.xhtml

For peer review only

However, operationalising the communication aspect of the NBS pathways had clear implications in terms of resources; communicating positive newborn screening results for the screened conditions could be time consuming for a range of reasons. These included, not being able to contact the appropriate person or needing to wait for the appropriate busy clinician to return a telephone call.

"Sometimes…you'd speak to a secretary and you'd be waiting for a doctor to get back to you. So, it can take a few hours from when you'd started to try and process that, depending on how quickly get back to you." Site 12

"..it does sometimes feel like a bit of a battle trying to get hold of someone." Site 3

Again, this was also seen as being condition specific**.**

"Once I know about a positive result [for CF or one of the metabolic conditions], ten, fifteen minutes….for CHT, I might be chasing around for an hour." Site 10

Due to the time-consuming nature of referring positive NBS results to the relevant professional or team, specialist screening professionals were highly valued by laboratory staff due to their knowledge of the conditions but also their ability to provide a link between the laboratory and the clinical teams. However, this service was not universally available.

"We don't have anything luxurious like a screening specialist nurse or anything like that, which I think probably would be extremely useful, but we don't." Site 2

Once the screening result has been received by the relevant member of the clinical team, a large amount of time is dedicated to organising and preparing for the family to be seen by the clinical team for the first time. The responsibility for this is shared and might be undertaken by the laboratory team, the consultant's secretary and/or the consultant or clinical nurse specialist for the specific condition. This was often centre specific and depends on local arrangements and resources.

Although quite unusual, in some areas, the NBS laboratory would be responsible for arranging the appointment and sometimes the diagnostic tests. In others, this would be the responsibility of the specialist screening coordinator, screening nurse or screening health visitor. In other

For peer review only - http://bmjopen.bmj.com/site/about/guidelines.xhtml

centres, this would be the responsibility of members of the relevant clinical team such as the consultant or specialist nurse or midwife.

Communication pathways:

Communication pathways for the 13 newborn screening laboratories in England for positive NBS results were developed from the interview responses. These described how positive NBS results were communicated from the NBS laboratory to parents via the clinical team and highlighted when variations occurred (Supplementary file A).

DISCUSSION

The findings of this study indicate that NBS laboratory staff are acutely aware of the significance of a positive NBS result and the potential impact this could have on the family, even though in the majority of cases, they do not have direct contact with the family. Despite this, some challenges existed when communicating results from laboratories to relevant clinicians. These could involve time-consuming processes being employed to ensure the NBS ‘communication loop’ from the laboratory to clinical teams to families and back to the laboratory was closed. Nevertheless, laboratory staff in the present study were willing to invest the additional time and effort needed to ensure the performance thresholds of the NBS Programme were met in particular in relation to referral of screen positive samples, and timely entry into clinical care for all screen positive babies referred to specialist services[25].

The communication pathway for positive NBS results starts in the laboratory via relevant clinical teams and ends with the family of the affected child. Many studies have explored communication of positive NBS results to families[11-16] but none have explored communication of NBS results between the laboratory and clinical teams involved in this process.

Although templates for the communication of positive NBS results exist, most laboratories had developed their own proformas, designed to meet local needs more explicitly. Although many laboratories stated these were based on the standard national templates, these had been adapted for a variety of reasons including: attempting to make the formatting of the proforma compatible with existing computer systems and data generated during processing of the NBS result; feedback from clinical teams regarding the content of information that would be useful

For peer review only

For peer review only - http://bmjopen.bmj.com/site/about/guidelines.xhtml

when receiving the NBS result; and the addition of information to assist laboratories to obtain information from clinical teams about when the baby had been seen so this information could be uploaded to the Child Health Information Service (CHIS) (clinical care records for children which contains information about a child's public health interventions, such as screening, immunisations and outcomes). However, this meant that for clinical teams who received positive NBS results from more than one laboratory, the information and format used varied. It is known that variations exist both nationally and internationally in terms of the approaches used to communicate positive NBS results to families [11-16] but this would also seem to extend to the approaches used to communicate positive NBS results between clinicians. In addition, laboratory staff could spend considerable time trying to locate and make a referral to the correct clinician. Finally, obtaining the necessary feedback from clinical teams to enable the laboratories to complete their reporting processes could also be time consuming and challenging. Although this is unlikely to influence communication with the family, it is important to ensure information relating to every child's NBS journey is documented in a timely fashion and is available to relevant professionals involved in the child's care. Interestingly, providing this feedback was not seen as being an issue by clinical staff which suggests that they may not be aware of the information that is needed and could explain why in some cases this is not fed back in a timely manner.

The communication pathways identified some key similarities and differences between the different NBS laboratories. Key similarities included: contacting the relevant clinical team via telephone or in person (depending on physical proximity to the NBS laboratory) to alert them of a potential positive NBS positive result prior to sending the formal proforma, normally via secure email; requesting feedback from clinical teams regarding when the baby had been seen and the outcome (although this could either be in the form of a locally generated feedback form or a request for a copy of the clinic letter generated after the initial consultation with the child and family); and automatic upload of screening outcomes to the CHIS. Differences included: who in the clinical team was contacted by the NBS laboratory. This included the Consultant, Registrar, the Clinical Nurse Specialist, Specialist Health Visitors / Midwives, Genetic Counsellors and Screening / Pathway Co-ordinators. In some instances, this was condition specific but often this was determined by local arrangements and availability of resources. The person responsible for arranging the logistics of the initial appointment with the family also varied from the Consultant to more commonly the Clinical Nurse Specialist to infrequently a member of laboratory staff or the Consultant's secretary.

For peer review only - http://bmjopen.bmj.com/site/about/guidelines.xhtml

This variability reflects those observed when positive NBS results are communicated to families [11-16]. Availability of specialist screening nurses who could act to bridge these processes by receiving the screening result, arrange the follow-up required and deliver the positive NBS result to the family, were highly valued by both laboratory staff and members of clinical teams and viewed as an example of good practice. Communication pathways for carrier results, the most common outcome for NBS screening particularly for SCD, also varied significantly by condition and locality.

Communicating positive NBS results for CHT seemed to be particularly problematic perhaps because communication is not always via a specialist clinical team due to CHT being viewed as manageable by general paediatricians. Different models of care are in operation throughout the country and how these operate seemed to be influenced by local arrangements and resources but also to some extent historical influences. This often led to difficulties from the laboratory perspective: who the correct person was to refer a baby with suspected CHT to; a lack of confidence on occasions once the referral had been made; and concern that the child may not be followed up according to national guidelines. However, performance data from 2017-2018[26 27] indicates that 92.7% of babies with a CHT positive screening result had a clinical referral initiated within three working days of sample receipt by the NBS laboratory, this compared to 100% of babies with MSUD, GA1, IVA, MCADD and 99.1% of babies with PKU. In addition, 93% of children with CHT entered clinical care in a timely manner, this was higher than for those babies with MSUD (50.0%), PKU 61.1%), HCU (66.7%), CF (66.8%) and MCADD (76.2%). Therefore, while these performance data indicate that difficulties communicating the positive NBS result for CHT may have hindered timely referral to relevant clinical teams, this did not delay initiation of clinical care. Other conditions have dedicated clinical teams that focus solely on that condition (CF) or range of conditions (SCD and the metabolic conditions) whereas babies with CHT are seen by endocrine teams who manage a range of other unrelated conditions in addition to CHT. As such. the performance data[26 27] indicates that referrals for these conditions were made more quickly although this was not reflected in the time taken to enter clinical care. However, this could also reflect the differing complexities of these conditions in terms of treatment. Another factor that was considered to improve the referral process particularly for the IMDs was the close working relationships both physically and personally between laboratory staff and clinical teams were seen to enhance the referral process. However, this could also pose a potential risk if for instance relationships deteriorate or people move jobs. This was not

For peer review only - http://bmjopen.bmj.com/site/about/guidelines.xhtml

evident in the present study but is perhaps something that needs careful consideration to avoid an overreliance on relationships rather than robust systems.

Strengths and Limitations:

The current study has numerous strengths. This is the first known study that has explored communication pathways for positive NBS results from the laboratory to clinical teams. Participants represented the 13 NBS laboratories in England involved in managing the nine conditions currently included in the NBS programme increasing the transferability of the findings. In addition, the study design, data collection and analysis were influenced by members of the PPI advisory group and relevant charities. In terms of limitations, the duration of the interviews ranged quite widely and the richness of the data collected was limited in the shorter interviews; in most instances, this was considered to reflect the limited experiences of some of the staff who were interviewed. The researchers are experienced in this field which may have biased data collection and analysis.

Recommendations for practice

The findings of this study suggest a range of recommended routes, with some key requirements for communication of positive NBS results from laboratories to clinicians, allowing for local variation and complexity, would be beneficial. This would ensure information sharing amongst the range of professionals involved nationally is optimised following a positive NBS result. This would include information required by laboratories to complete their processes, which would reduce demand on resources currently used to ensure this information is collected. Further research is needed to explore communication pathways for positive NBS results for CHT to ensure the process is streamlined and clear communication pathways are in place. Whilst it is acknowledged that a single approach for processing CHT results may not be appropriate or necessary, transparency and complete information such as named contact individuals may help to ensure the process is less labour intensive particularly from a laboratory perspective. In addition, to reduce variability in communication practices, the inclusion of specialist screening nurses as part of the NBS laboratory team was viewed as an example of good practice.

CONCLUSIONS

For peer review only - http://bmjopen.bmj.com/site/about/guidelines.xhtml

The importance of ensuring timely and effective communication of positive NBS results from laboratories to families via relevant clinicians was seen as a priority for all involved. Variation exists in terms of proformas and processes used to report positive NBS results to clinical teams, the approaches used, and the people involved. This was often determined by local arrangements and resources. These different approaches could be time consuming and challenging. Despite these challenges, ensuring quality and consistency was a clear priority for all NBS laboratories.

For peer review only

For peer review only - http://bmjopen.bmj.com/site/about/guidelines.xhtml

Acknowledgements: The authors would like to thank the Newborn Screening Laboratory Directors in England for agreeing to act as local Principal Investigators for this study. The authors would also like to thank all the parents in the Public and Patient Involvement Advisory Group for this study for their invaluable input.

Contributors: JC made substantial contributions to the conception and design of the work. She acquired and interpreted the data for the work. She was involved in drafting the work, approved the final version to be published and agrees to be accountable for all aspects of the work in ensuring that questions related to the accuracy or integrity of any part of the work are appropriately investigated and resolved. AS and SM made substantial contributions to the conception and design of the work. They were involved in drafting the work, approved the final version to be published and agree to be accountable for all aspects of the work in ensuring that questions related to the accuracy or integrity of any part of the work are appropriately investigated and resolved. EKO, PH and HC were involved interpreting the data for the work and revising the work critically for important intellectual content, approved the final version to be published and agree to be accountable for all aspects of the work in ensuring that questions related to the accuracy or integrity of any part of the work are appropriately investigated and resolved. LM, FU, MB, RSC, and KWS made substantial contributions to the conception and design of the work. They were involved in drafting the work, approved the final version to be published and agree to be accountable for all aspects of the work in ensuring that questions related to the accuracy or integrity of any part of the work are appropriately investigated and resolved.

Funding: This study is funded by the National Institute for Health Research (NIHR) [Health Services and Delivery Research (project reference 16/52/25)]. The views expressed are those of the authors and not necessarily those of the NIHR or the Department of Health and Social Care.

Competing interests: None declared.

Ethics approval: This study was approved by the London Stanmore ethics committee, reference 231291

For peer review only

Data Sharing statement: Data are available upon reasonable request from the corresponding author subject to restrictions to preserve anonymity and personal privacy [JC]. These data are not publicly available as they contain information that could compromise research participant privacy/consent

Data will be available beginning 1 year and ending 5 years after publication to researchers who propose a methodologically sound proposal. Proposals should be directed to j.chudleigh@city.ac.uk. To gain access, data requesters will need to sign a data access agreement.

For peer review only

For peer review only - http://bmjopen.bmj.com/site/about/guidelines.xhtml

References

1. UK Newborn Screening Programme Centre. Health Professional Handbook: A guide to newborn blood spot screening for healthcare professionals. London: UK Newborn Screening Programme Centre, 2012:1-56.
2. Bush A. Newborn screening for cystic fibrosis – benefit or bane? *Paediatric respiratory reviews* 2008;9:301-2.
3. Public Health England. Newborn Blood Spot Screening Programme in the UK: Data Collection and Performance Analysis Report 2016 to 2017. London, 2018:1-65.
4. Public Health England. NHS Sickle Cell and Thalassaemia Screening Programme Data Report 2015/16: Trends and performance analysis. London, 2017:1-81.
5. Public Health England. NHS Newborn Blood Spot Screening Programme: Managing positive results from cystic fibrosis screening. London, 2017:1-20.
6. Public Health England. NHS Newborn Blood Spot Screening Programme A laboratory guide to newborn blood spot screening for inherited metabolic diseases. London, 2017:1-140.
7. Public Health England. NHS Sickle Cell and Thalassaemia Screening Programme Handbook for newborn laboratories. London, 2017:1-32.
8. UK National Screening Committee. A Laboratory Guide to Newborn Screening in the UK for Congenital Hypothyroidism 1st Edition ed. England, 2014:1-39.
9. UK Newborn Screening Programme Centre. A Laboratory Guide to Newborn Screening in the UK for Cystic Fibrosis. 4th Edition ed. London, 2014:1-35.
10. Chudleigh J, Chinnery H, Bonham JR, et al. Qualitative exploration of health professionals' experiences of communicating positive newborn bloodspot screening results for nine conditions in England. *BMJ open* 2020;10(10):e037081. doi: 10.1136/bmjopen-2020-037081 [published Online First: 2020/10/03]
11. Chudleigh J, Buckingham S, Dignan J, et al. Parents' Experiences of Receiving the Initial Positive Newborn Screening (NBS) Result for Cystic Fibrosis and Sickle Cell Disease. *J Genet Couns* 2016;25(6):1215-26. doi: 10.1007/s10897-016-9959-4
12. Parker H, Qureshi N, Ulph F, et al. Imparting carrier status results detected by universal newborn screening for sickle cell and cystic fibrosis in England: a qualitative study of current practice and policy challenges. *BMC Health Serv Res* 2007;7:203. doi: 1472-6963-7-203 [pii] 10.1186/1472-6963-7-203 [published Online First: 2007/12/15]
13. Ulph F, Cullinan T, Qureshi N, et al. The impact on parents of receiving a carrier result for sickle cell or cystic fibrosis for their child via newborn screening. *Eur J Hum Genet* 2014;22
14. Chudleigh J, Ren CL, Barben J, et al. International approaches for delivery of positive newborn bloodspot screening results for CF. *Journal of cystic fibrosis : official journal of the European Cystic Fibrosis Society* 2019;18(5):614-21. doi: 10.1016/j.jcf.2019.04.004
15. Kai J, Ulph F, Cullinan T, et al. Communication of carrier status information following universal newborn screening for sickle cell disorders and cystic fibrosis: qualitative study of experience and practice. *Health Technol Assess* 2009;13(57):1-82, iii. doi: 10.3310/hta13570 [published Online First: 2009/12/02]
16. Ulph F, Cullinan T, Qureshi N, et al. Parents' responses to receiving sickle cell or cystic fibrosis carrier results for their child following newborn screening. *Eur J Hum Genet* 2015;23(4):459-65. doi: DOI 10.1038/ejhg.2014.126
17. Buchbinder M, Timmermans S. Newborn screening for metabolic disorders: parental perceptions of the initial communication of results. *Clin Pediatr (Phila)* 2012;51(8):739-44. doi: 0009922812446011 [pii] 10.1177/0009922812446011 [published Online First: 2012/05/09]
18. Salm A, Yetter E, Tluczek A. Informing parents about positive newborn screening results: Parents' recommendations *Journal of Child Health Care* 2012;16(4):367-81.
19. Rueegg CS, Barben J, Hafen GM, et al. Newborn screening for cystic fibrosis - The parent perspective. *Journal of cystic fibrosis : official journal of the European Cystic Fibrosis Society* 2016;15(4):443-51. doi: 10.1016/j.jcf.2015.12.003

For peer review only - http://bmjopen.bmj.com/site/about/guidelines.xhtml

20. Tluczek A, Clark R, McKechnie AC, et al. Factors affecting parent-child relationships one year after positive newborn screening for cystic fibrosis or congenital hypothyroidism. *J Dev Behav Pediatr* 2015;36(1):24-34. doi: 10.1097/DBP.0000000000000112
21. Chudleigh J, Bonham J, Bryon M, et al. Rethinking Strategies for Positive Newborn Screening Result (NBS+) Delivery (ReSPoND): a process evaluation of co-designing interventions to minimise impact on parental emotional well-being and stress. *Pilot Feasibility Stud* 2019;5:108. doi: 10.1186/s40814-019-0487-5
22. Braun V, Clarke V. Using thematic analysis in psychology. *Qualitative Research in Psychology* 2006;3(2):77-101.
23. Milford C, Kriel Y, Njau I, et al. Teamwork in Qualitative Research: Descriptions of a Multicountry Team Approach. *International Journal of Qualitative Methods* 2017;16:1-10.
24. DeCuir-Gunby JT, Marshall PL, McCulloch AW. Developing and Using a Codebook for the Analysis of Interview Data: An Example from a Professional Development Research Project. *Field Methods* 2011;23(2):136-55.
25. Public Health England. Guidance Newborn blood spot screening standards valid for data collected from 1 April 2020 London: Public Health England, 2020.
26. Public Health England. Newborn blood spot screening: data collection report 2017 to 2018. London: Public Health England, 2020.
27. Public Health England. Sickle cell and thalassaemia screening: data report 2017 to 2018. London: Public Health England, 2020.

For peer review only

For peer review only - http://bmjopen.bmj.com/site/about/guidelines.xhtml

NBSL Interview Guide

1. In which laboratory do you work

2. What is your job title?

3. When did your lab start processing NBS results?

4. How long have you been involved in processing NBS results?

5. Please can you describe the process you follow when you receive a NBS card for processing in the lab?

    Prompts

    a. Does this differ depending on the condition being screened for?
    b. Is there a time frame this needs to be completed within?

6. For each of the screened conditions, can you clarify what would be considered a positive NBS result?
    a. Sickle cell disease (SCD)
    b. Cystic Fibrosis (CF)
    c. Congenital Hypothyroidism (CHT)
    d. phenylketonuria (PKU)
    e. medium-chain acyl-CoA dehydrogenase deficiency (MCADD)
    f. maple syrup urine disease (MSUD)
    g. isovaleric acidaemia (IVA)
    h. glutaric aciduria type 1 (GA1)
    i. homocystinuria (pyridoxine unresponsive) (HCU)

7. When you have a positive result, what is the next step?

8. Who and how are the clinical team contacted to inform them of a positive NBS result?

    Prompts

    a. Telephone/email/letter?
    b. Does this differ for the different conditions?

9. For each of the screened conditions, what information do you convey to the clinical team?
    a. Sickle cell disease (SCD)
    b. Cystic Fibrosis (CF)
    c. Congenital Hypothyroidism (CHT)
    d. phenylketonuria (PKU)
    e. medium-chain acyl-CoA dehydrogenase deficiency (MCADD)
    f. maple syrup urine disease (MSUD)
    g. isovaleric acidaemia (IVA)
    h. glutaric aciduria type 1 (GA1)
    i. homocystinuria (pyridoxine unresponsive) (HCU)

For peer review only

10. What happens if you are unable to get hold of the relevant clinical team?

11. Do you advise anyone else of the positive NBS result?

Clinical Team Interview Guide

1. In which hospital do you work?

2. What is your job title?

3. For which of the nine screened conditions are you responsible for communicating positive NBS results?

    a. Sickle cell disease (SCD)
    b. Cystic Fibrosis (CF)
    c. Congenital Hypothyroidism (CHT)
    d. phenylketonuria (PKU)
    e. medium-chain acyl-CoA dehydrogenase deficiency (MCADD)
    f. maple syrup urine disease (MSUD)
    g. isovaleric acidaemia (IVA)
    h. glutaric aciduria type 1 (GA1)
    i. homocystinuria (pyridoxine unresponsive) (HCU)

4. How long have you been involved in communicating positive NBS results to families?

5. Who initially communicates the positive NBS result for X condition to the family?

6. How is this information initially communicated to the family?

    Prompts

    a. Letter, from
    b. Email, from
    c. Phone call, from
    d. Other…..

7. When the positive NBS result is communicated to the family in this way, what information is provided?

    Prompts
    a. Where to go
    b. Contact details for
    c. Information about the condition (life expectancy, genetic information, treatment, severity)
    d. Use of information sources (written, verbal, internet)

For peer review only

8. Is any further contact made with the family prior to them being seen in clinic/hospital
   Prompts
   e.g. home visit, follow-up phone call?
   How does this happen?

9. Do you inform anyone else of the result either before or after you speak with the child's family?
   Prompts
   e.g. HV or GP?
   If yes, how, when and why? If no, why not?

10. What happens after the family is told that their child has a positive NBS result for X condition?
    Prompts
    a. Is the child admitted?
    b. How quickly are they seen by the specialist team?
    c. Are any other tests carried out to confirm the diagnosis? When and how?
    d. How often are they followed up?

11. What other members of the MDT do you involve at this stage?
    Prompts
    e.g. refer to dietician, genetic counselling, physiotherapy etc

12. What do you think works well in terms of the way you currently communicate positive NBS results for x condition to the family?

13. If possible, is there anything you would like to change about the way you communicate positive NBS results for x condition to the family?

For peer review only

Site 1

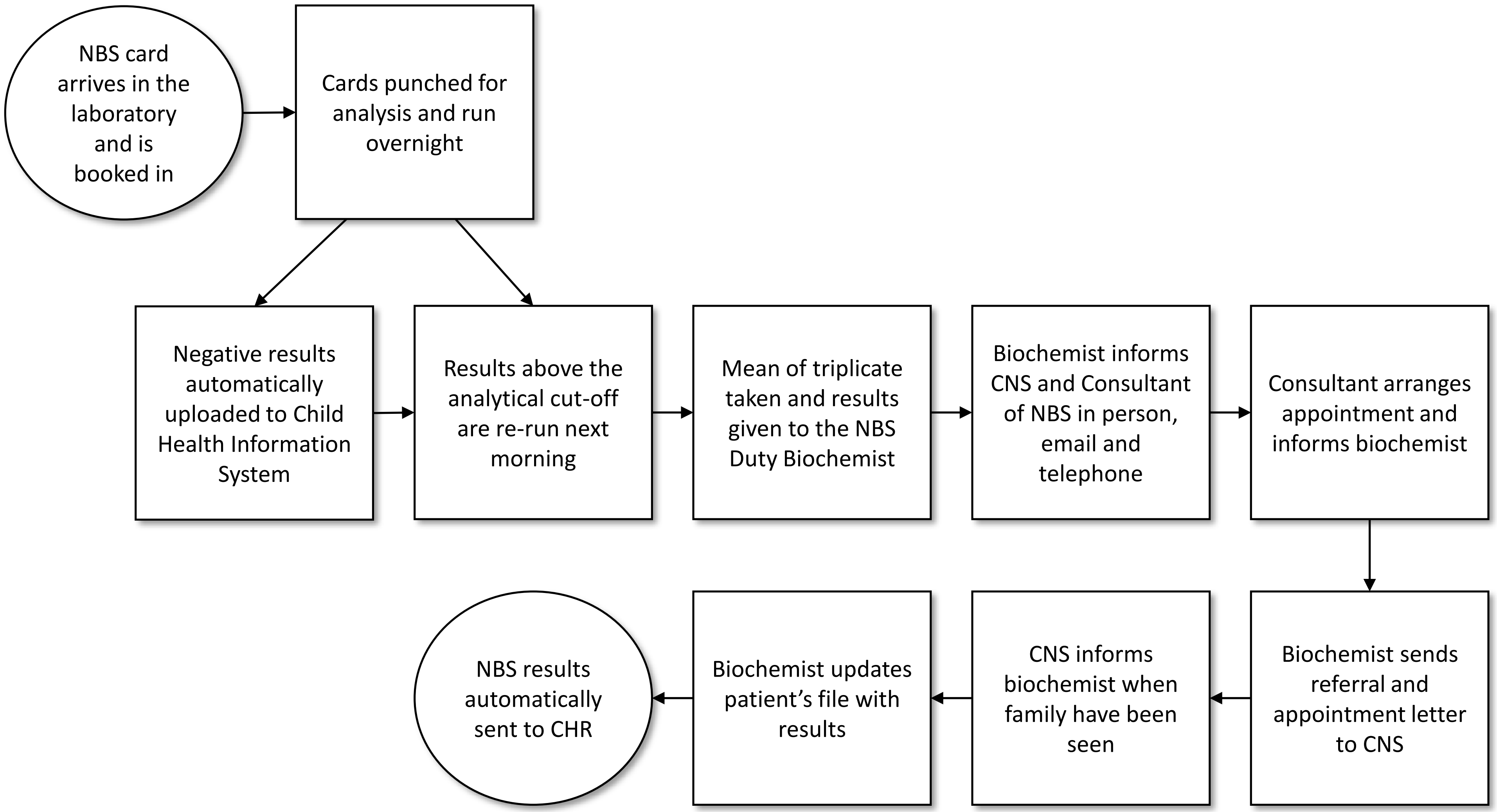
NBS card arrives in the laboratory and is booked in
Cards punched for analysis and run overnight
Negative results automatically uploaded to Child Health Information System
Results above the analytical cut-off are re-run next morning
Mean of triplicate taken and results given to the NBS Duty Biochemist
Biochemist informs CNS and Consultant of NBS in person, email and telephone
Consultant arranges appointment and informs biochemist
Biochemist sends referral and appointment letter to CNS
CNS informs biochemist when family have been seen
Biochemist updates patient's file with results
NBS results automatically sent to CHR

Site 2

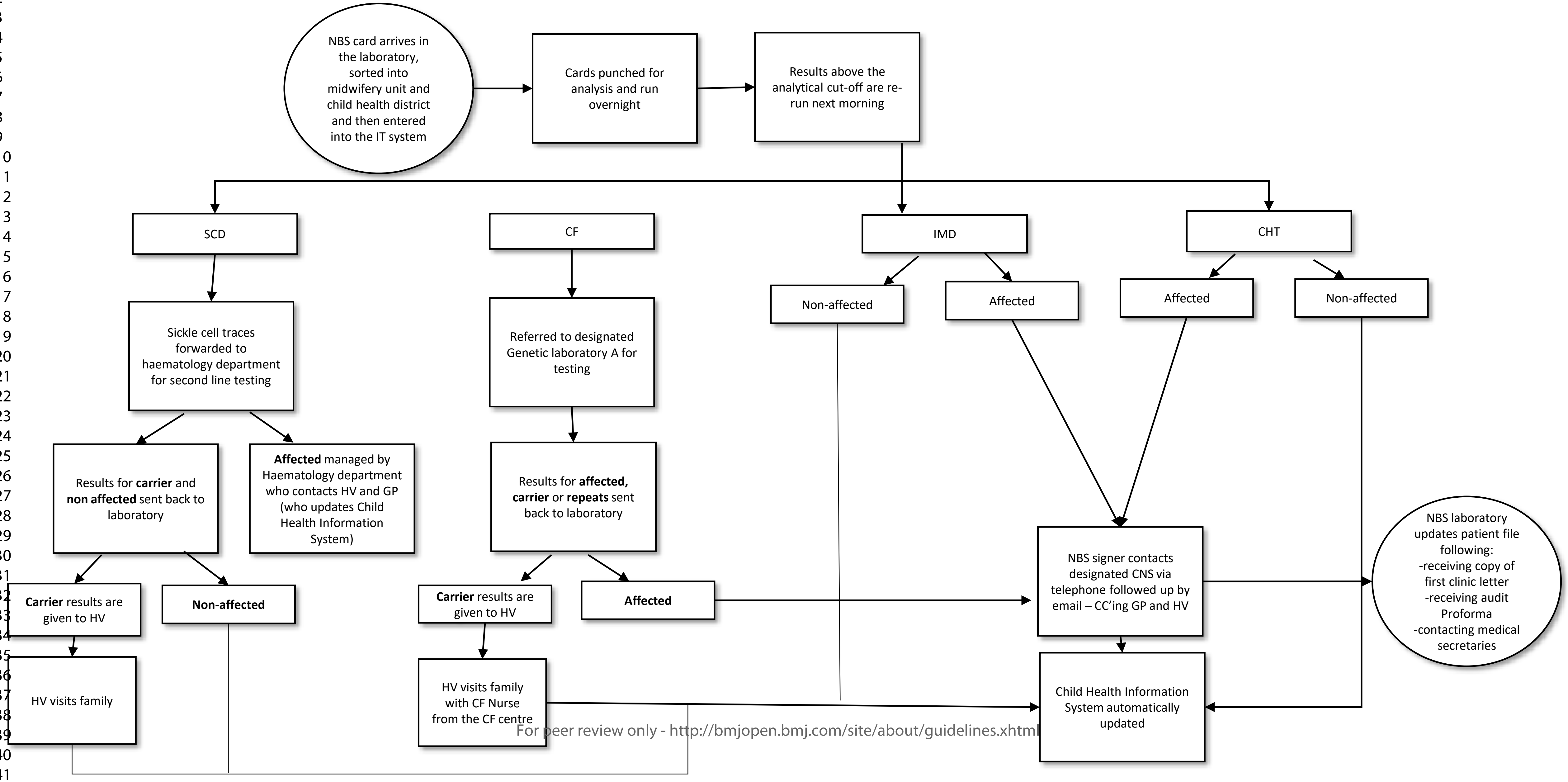
NBS card arrives in the laboratory, sorted into midwifery unit and child health district and then entered into the IT system
Cards punched for analysis and run overnight
Results above the analytical cut-off are re-run next morning
SCD
CF
IMD
CHT
Non-affected
Affected
Affected
Non-affected
Sickle cell traces forwarded to haematology department for second line testing
Referred to designated Genetic laboratory A for testing
Results for carrier and non affected sent back to laboratory
Affected managed by Haematology department who contacts HV and GP (who updates Child Health Information System)
Results for affected, carrier or repeats sent back to laboratory
Carrier results are given to HV
Non-affected
Carrier results are given to HV
Affected
NBS signer contacts designated CNS via telephone followed up by email – CC'ing GP and HV
NBS laboratory updates patient file following:
-receiving copy of first clinic letter
-receiving audit Proforma
-contacting medical secretaries
HV visits family
HV visits family with CF Nurse from the CF centre
Child Health Information System automatically updated

Site 3

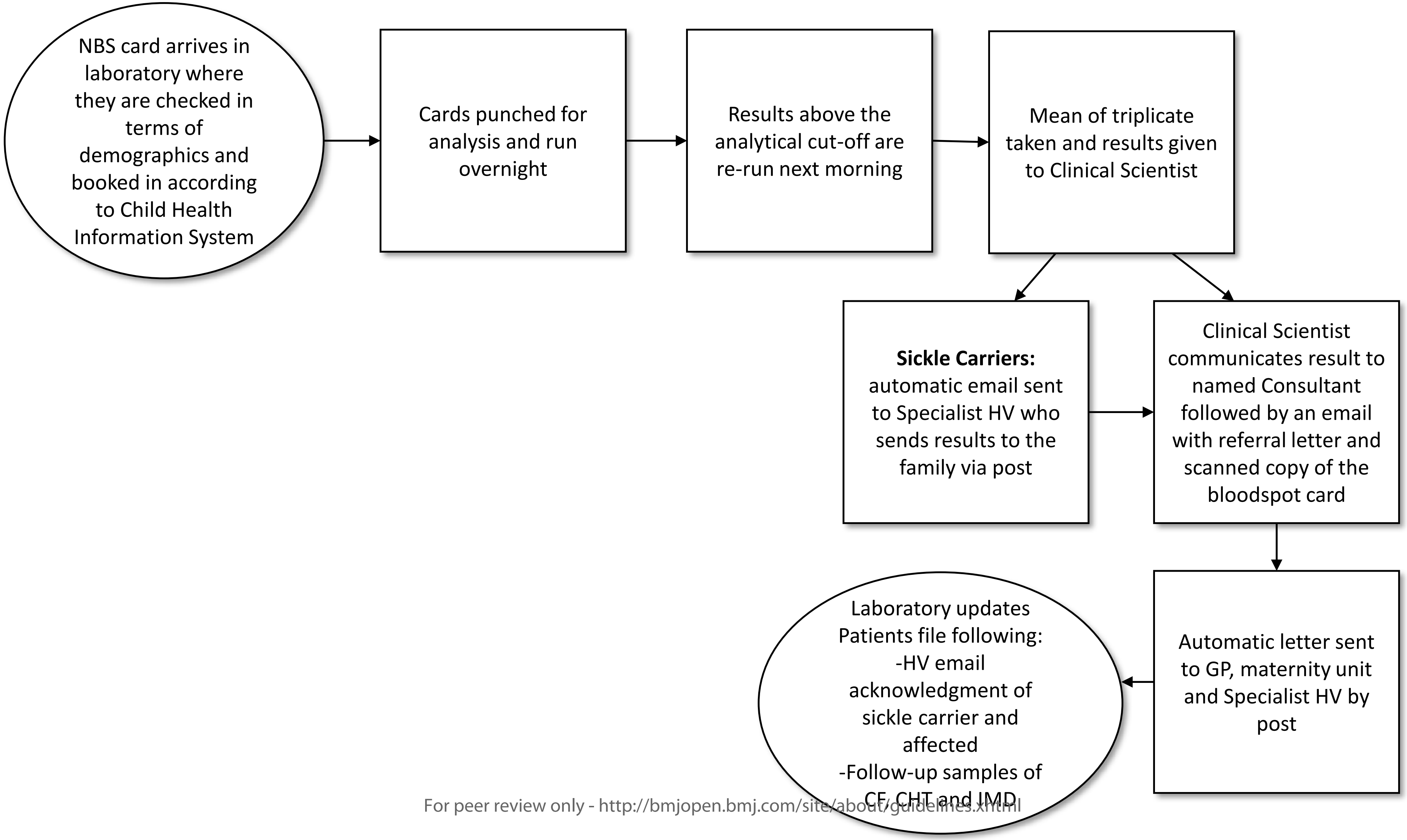
NBS card arrives in laboratory where they are checked in terms of demographics and booked in according to Child Health Information System
Cards punched for analysis and run overnight
Results above the analytical cut-off are re-run next morning
Mean of triplicate taken and results given to Clinical Scientist
Sickle Carriers: automatic email sent to Specialist HV who sends results to the family via post
Clinical Scientist communicates result to named Consultant followed by an email with referral letter and scanned copy of the bloodspot card
Automatic letter sent to GP, maternity unit and Specialist HV by post
Laboratory updates Patients file following:
-HV email acknowledgment of sickle carrier and affected
-Follow-up samples of CF, CHT and IMD

Site 4

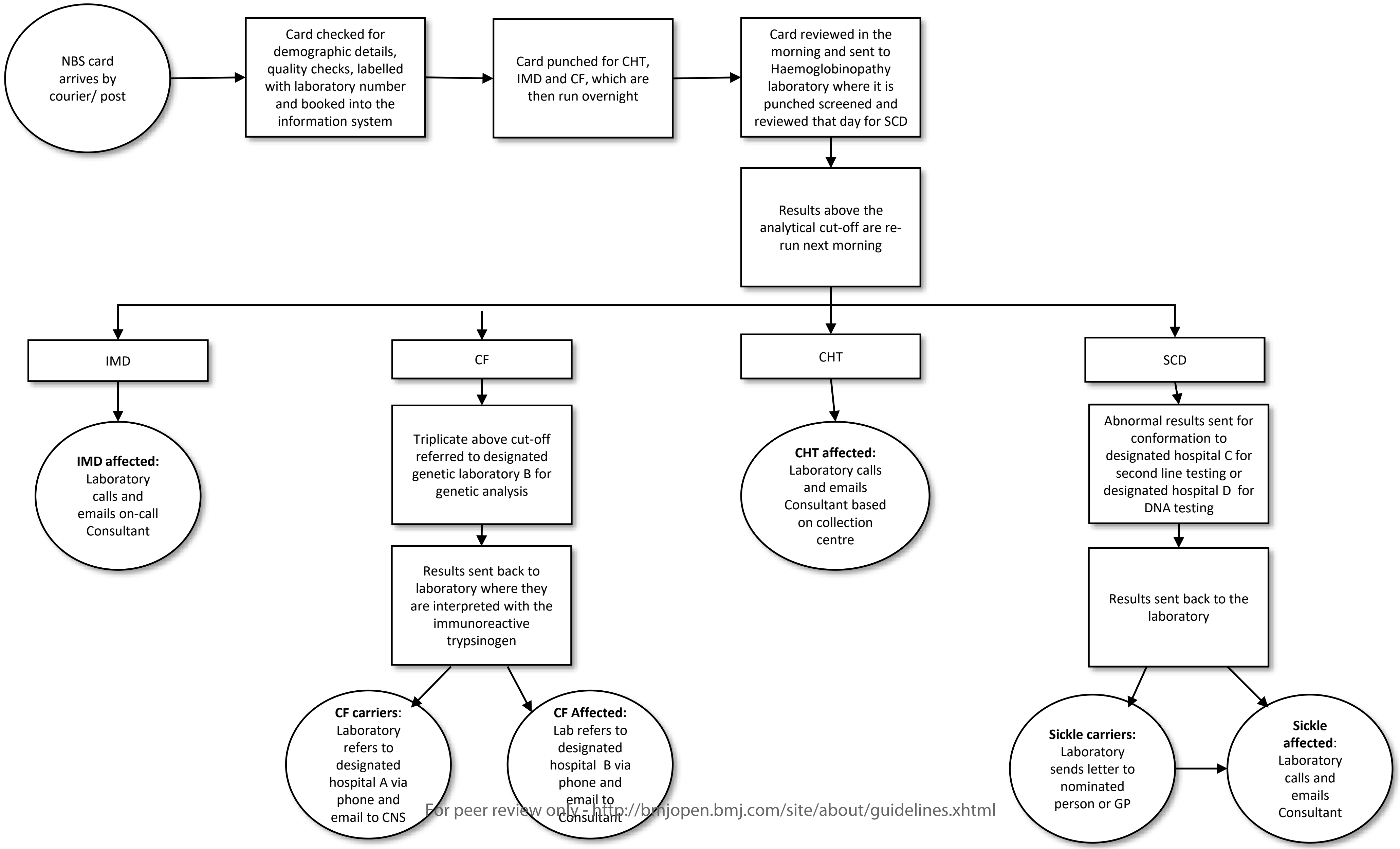
NBS card arrives by courier/ post
Card checked for demographic details, quality checks, labelled with laboratory number and booked into the information system
Card punched for CHT, IMD and CF, which are then run overnight
Card reviewed in the morning and sent to Haemoglobinopathy laboratory where it is punched screened and reviewed that day for SCD
Results above the analytical cut-off are re-run next morning
IMD
CF
CHT
SCD
IMD affected: Laboratory calls and emails on-call Consultant
Triplicate above cut-off referred to designated genetic laboratory B for genetic analysis
Results sent back to laboratory where they are interpreted with the immunoreactive trypsinogen
CF carriers: Laboratory refers to designated hospital A via phone and email to CNS
CF Affected: Lab refers to designated hospital B via phone and email to Consultant
CHT affected: Laboratory calls and emails Consultant based on collection centre
Abnormal results sent for conformation to designated hospital C for second line testing or designated hospital D for DNA testing
Results sent back to the laboratory
Sickle carriers: Laboratory sends letter to nominated person or GP
Sickle affected: Laboratory calls and emails Consultant

Site 5

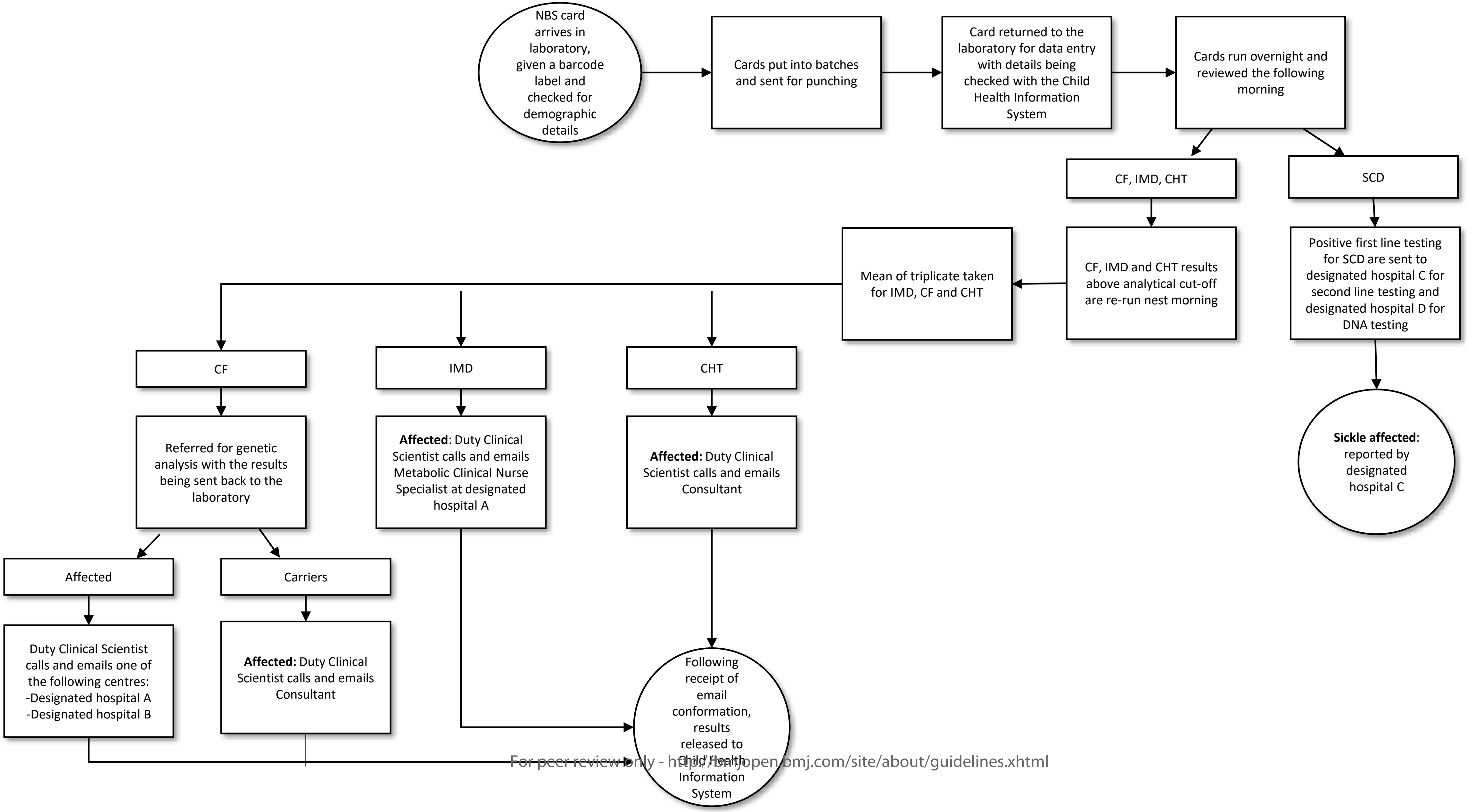

For peer review only - http://bmjopen.bmj.com/site/about/guidelines.xhtml

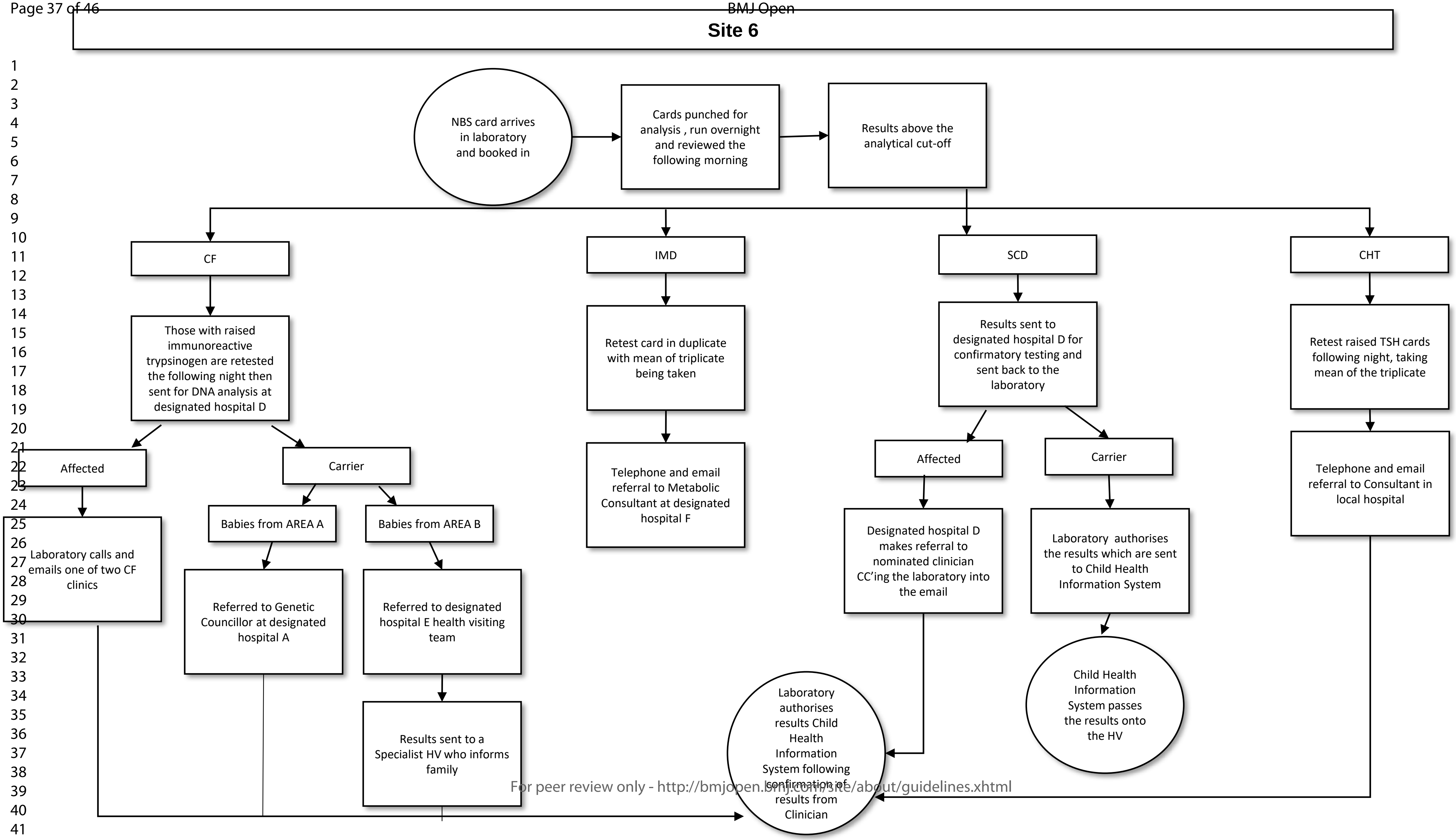

For peer review only - http://bmjopen.bmj.com/site/about/guidelines.xhtml

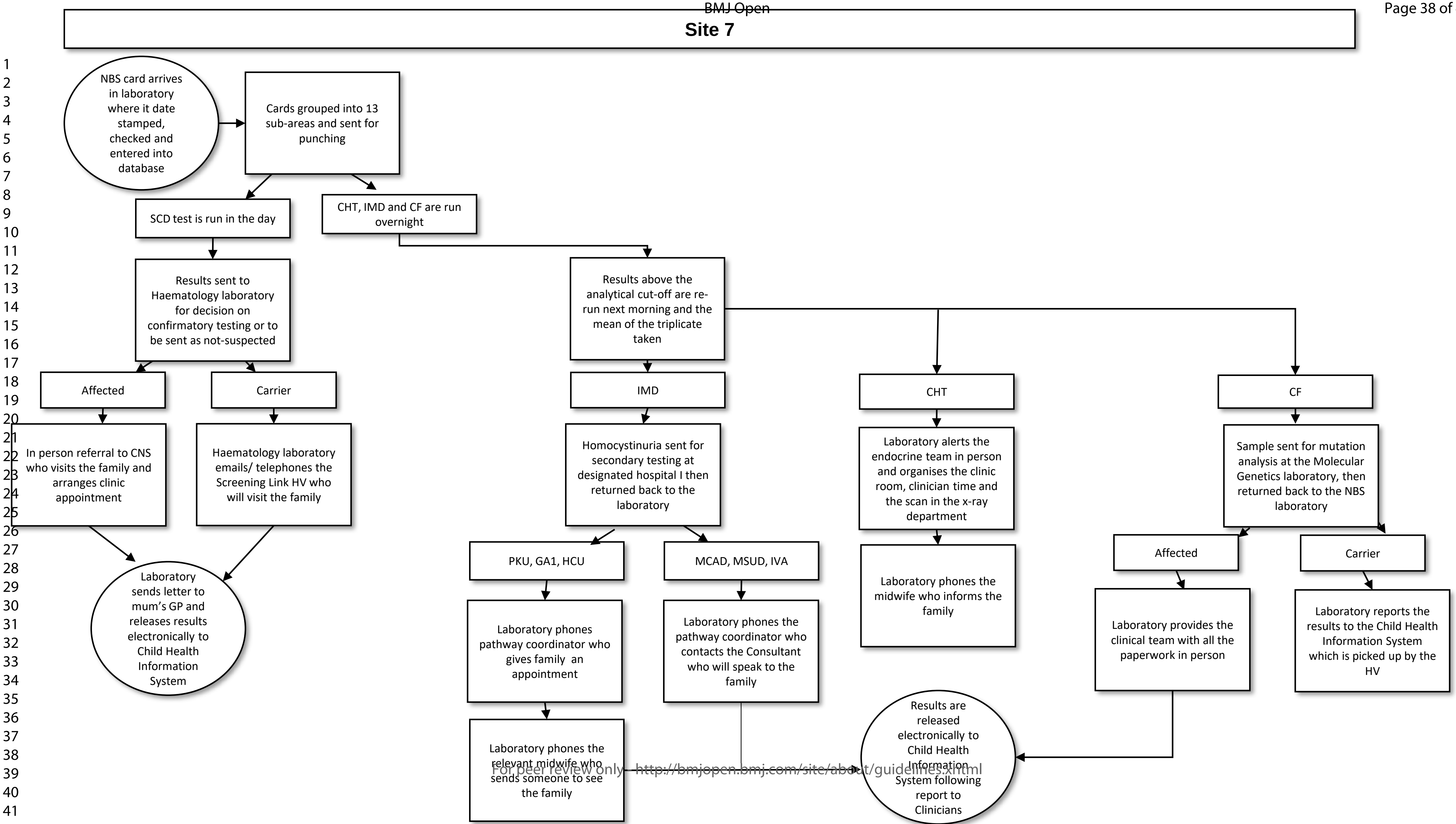
Site 7
NBS card arrives in laboratory where it date stamped, checked and entered into database
Cards grouped into 13 sub-areas and sent for punching
SCD test is run in the day
CHT, IMD and CF are run overnight
Results sent to Haematology laboratory for decision on confirmatory testing or to be sent as not-suspected
Results above the analytical cut-off are re-run next morning and the mean of the triplicate taken
Affected
Carrier
IMD
CHT
CF
In person referral to CNS who visits the family and arranges clinic appointment
Haematology laboratory emails/ telephones the Screening Link HV who will visit the family
Homocystinuria sent for secondary testing at designated hospital I then returned back to the laboratory
Laboratory alerts the endocrine team in person and organises the clinic room, clinician time and the scan in the x-ray department
Sample sent for mutation analysis at the Molecular Genetics laboratory, then returned back to the NBS laboratory
PKU, GA1, HCU
MCAD, MSUD, IVA
Affected
Carrier
Laboratory sends letter to mum's GP and releases results electronically to Child Health Information System
Laboratory phones the midwife who informs the family
Laboratory phones pathway coordinator who gives family an appointment
Laboratory phones the pathway coordinator who contacts the Consultant who will speak to the family
Laboratory provides the clinical team with all the paperwork in person
Laboratory reports the results to the Child Health Information System which is picked up by the HV
Laboratory phones the relevant midwife who sends someone to see the family
Results are released electronically to Child Health Information System following report to Clinicians

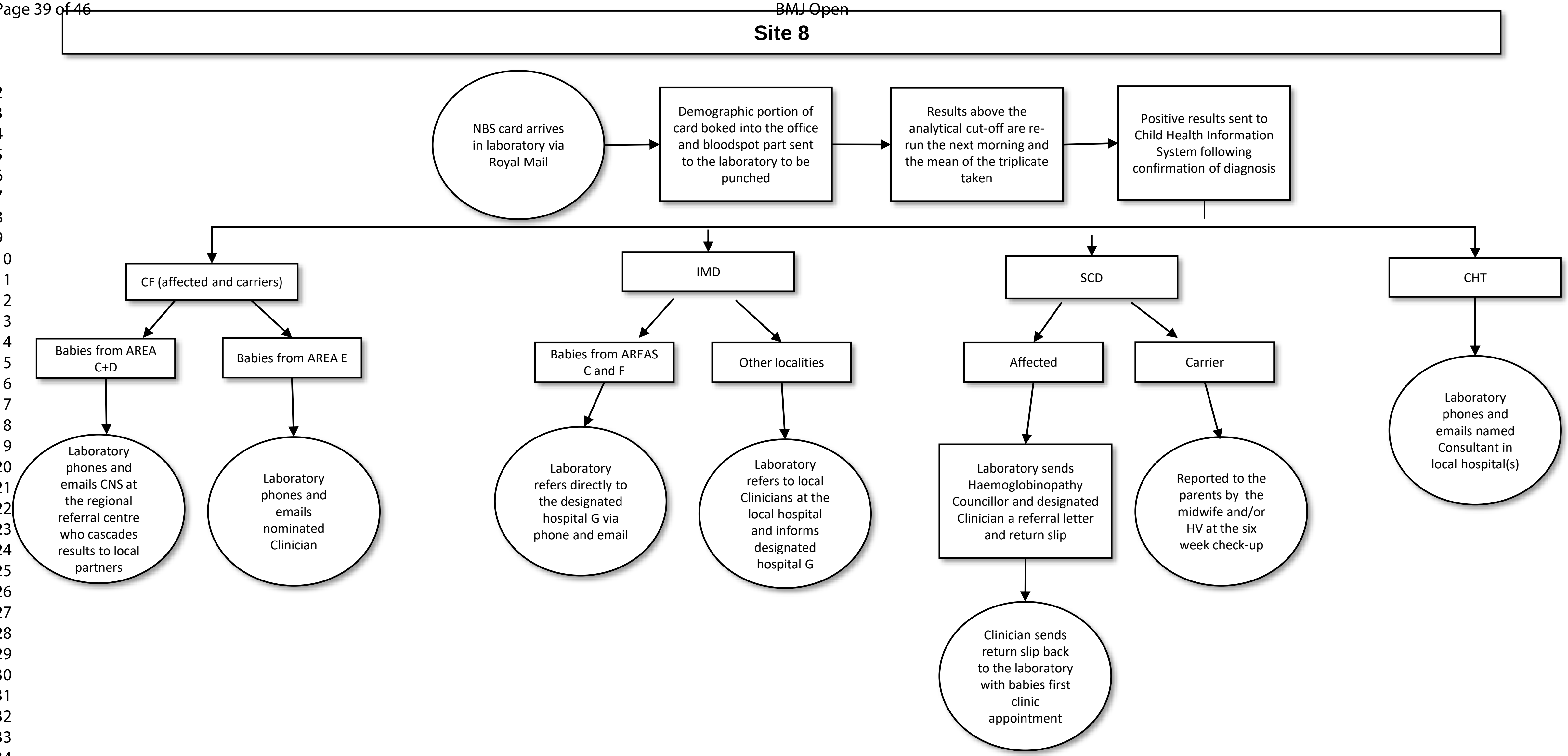
Site 8
NBS card arrives in laboratory via Royal Mail
Demographic portion of card boked into the office and bloodspot part sent to the laboratory to be punched
Results above the analytical cut-off are re-run the next morning and the mean of the triplicate taken
Positive results sent to Child Health Information System following confirmation of diagnosis
CF (affected and carriers)
IMD
SCD
CHT
Babies from AREA C+D
Babies from AREA E
Babies from AREAS C and F
Other localities
Affected
Carrier
Laboratory phones and emails named Consultant in local hospital(s)
Laboratory phones and emails CNS at the regional referral centre who cascades results to local partners
Laboratory phones and emails nominated Clinician
Laboratory refers directly to the designated hospital G via phone and email
Laboratory refers to local Clinicians at the local hospital and informs designated hospital G
Laboratory sends Haemoglobinopathy Councillor and designated Clinician a referral letter and return slip
Reported to the parents by the midwife and/or HV at the six week check-up
Clinician sends return slip back to the laboratory with babies first clinic appointment

Site 9

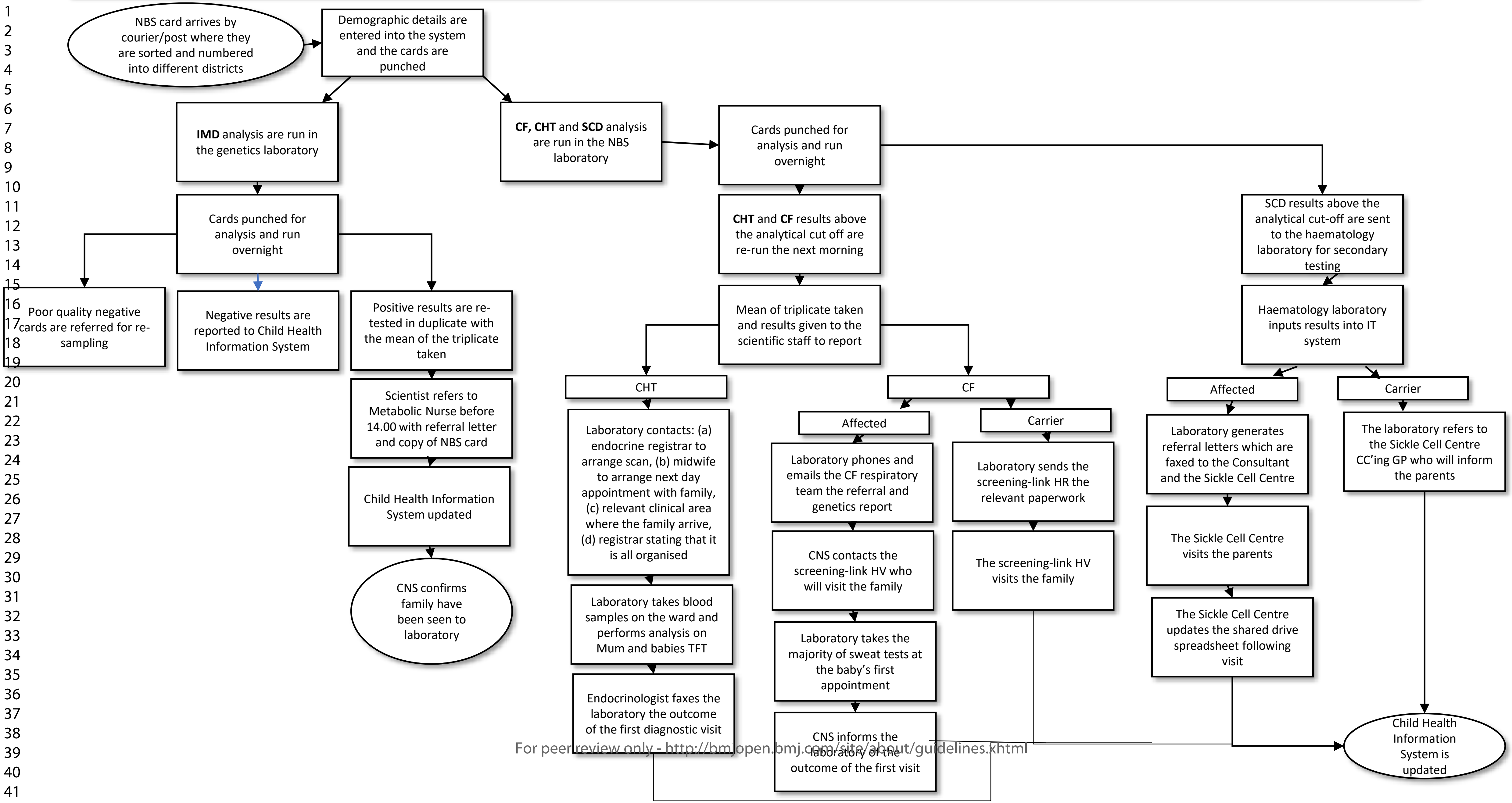

For peer review only - http://bmjopen.bmj.com/site/about/guidelines.xhtml

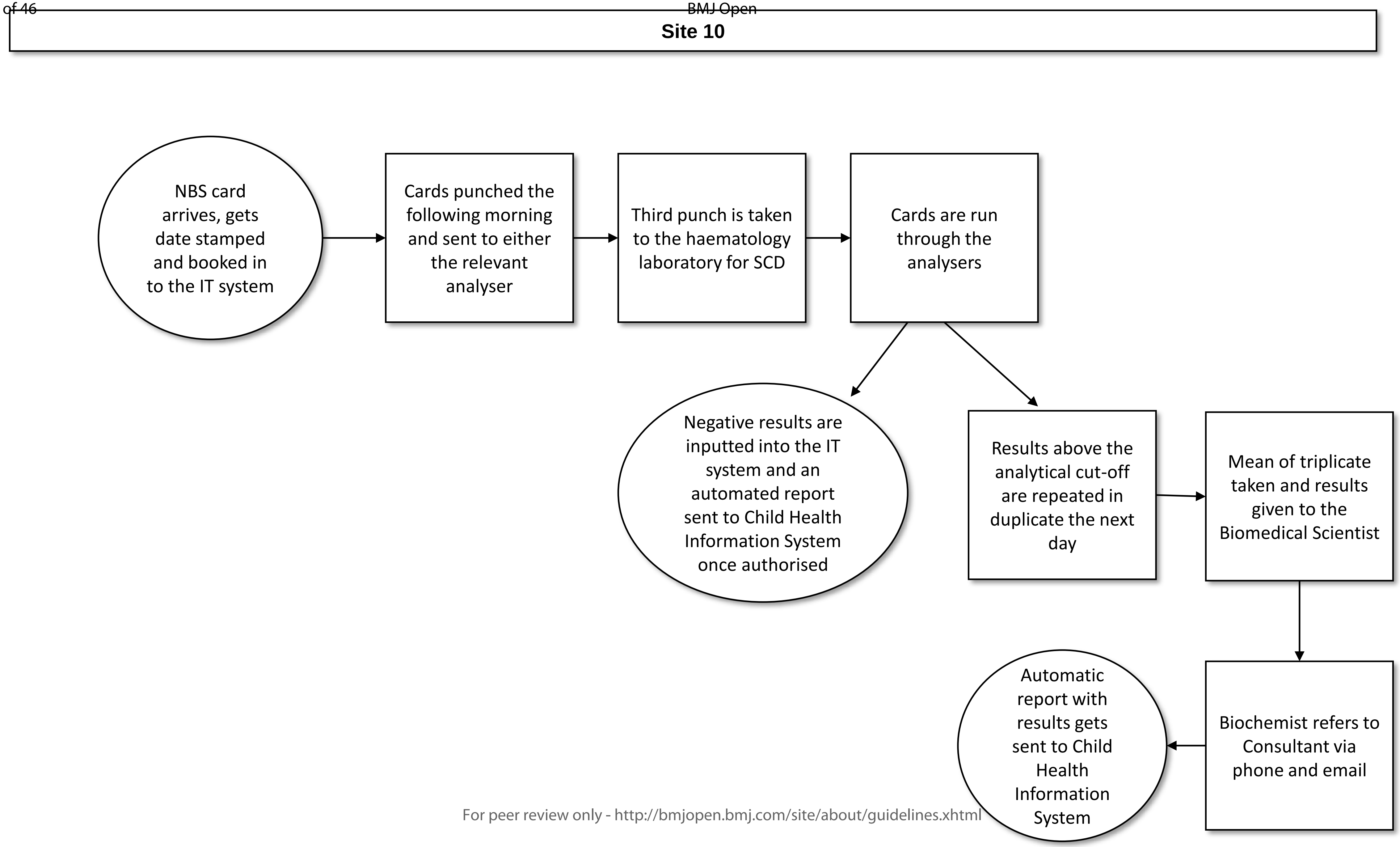
Site 10
NBS card arrives, gets date stamped and booked in to the IT system
Cards punched the following morning and sent to either the relevant analyser
Third punch is taken to the haematology laboratory for SCD
Cards are run through the analysers
Negative results are inputted into the IT system and an automated report sent to Child Health Information System once authorised
Results above the analytical cut-off are repeated in duplicate the next day
Mean of triplicate taken and results given to the Biomedical Scientist
Biochemist refers to Consultant via phone and email
Automatic report with results gets sent to Child Health Information System

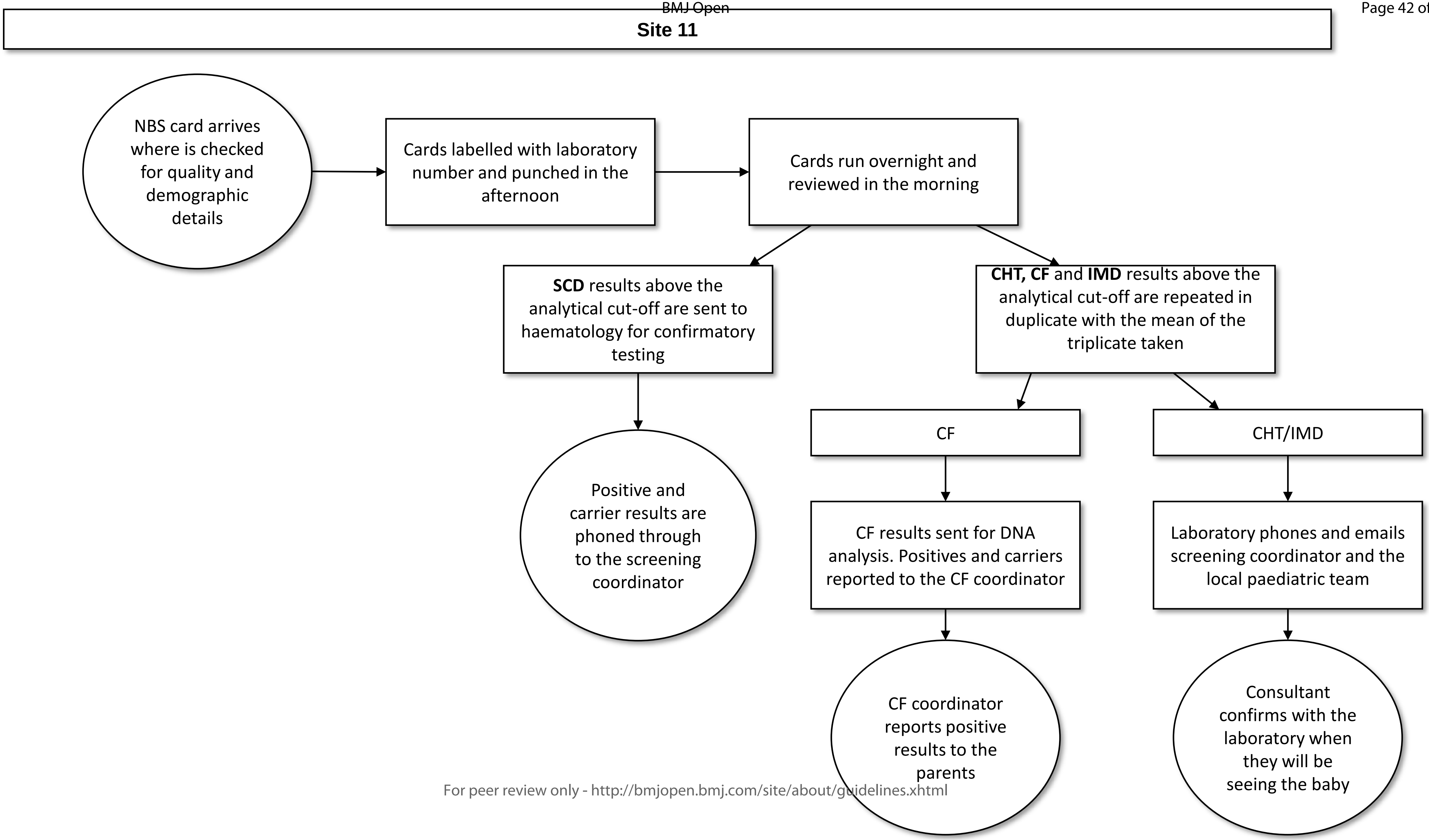
Site 11
NBS card arrives where is checked for quality and demographic details
Cards labelled with laboratory number and punched in the afternoon
Cards run overnight and reviewed in the morning
SCD results above the analytical cut-off are sent to haematology for confirmatory testing
CHT, CF and IMD results above the analytical cut-off are repeated in duplicate with the mean of the triplicate taken
Positive and carrier results are phoned through to the screening coordinator
CF
CHT/IMD
CF results sent for DNA analysis. Positives and carriers reported to the CF coordinator
Laboratory phones and emails screening coordinator and the local paediatric team
CF coordinator reports positive results to the parents
Consultant confirms with the laboratory when they will be seeing the baby

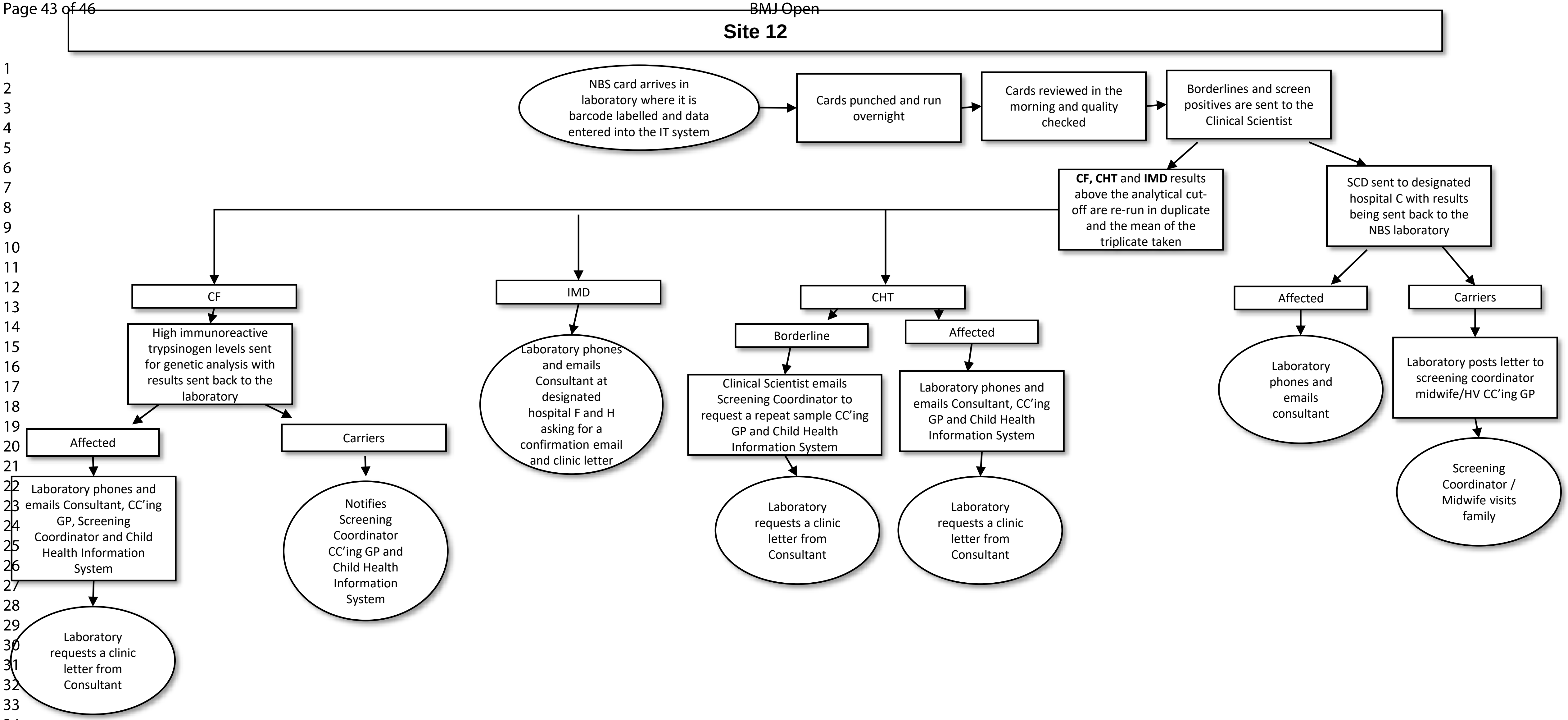
Site 12
NBS card arrives in laboratory where it is barcode labelled and data entered into the IT system
Cards punched and run overnight
Cards reviewed in the morning and quality checked
Borderlines and screen positives are sent to the Clinical Scientist
CF, CHT and IMD results above the analytical cut-off are re-run in duplicate and the mean of the triplicate taken
SCD sent to designated hospital C with results being sent back to the NBS laboratory
CF
IMD
CHT
Affected
Carriers
High immunoreactive trypsinogen levels sent for genetic analysis with results sent back to the laboratory
Laboratory phones and emails Consultant at designated hospital F and H asking for a confirmation email and clinic letter
Borderline
Affected
Laboratory phones and emails consultant
Laboratory posts letter to screening coordinator midwife/HV CC'ing GP
Clinical Scientist emails Screening Coordinator to request a repeat sample CC'ing GP and Child Health Information System
Laboratory phones and emails Consultant, CC'ing GP and Child Health Information System
Affected
Carriers
Screening Coordinator / Midwife visits family
Laboratory phones and emails Consultant, CC'ing GP, Screening Coordinator and Child Health Information System
Notifies Screening Coordinator CC'ing GP and Child Health Information System
Laboratory requests a clinic letter from Consultant
Laboratory requests a clinic letter from Consultant
Laboratory requests a clinic letter from Consultant

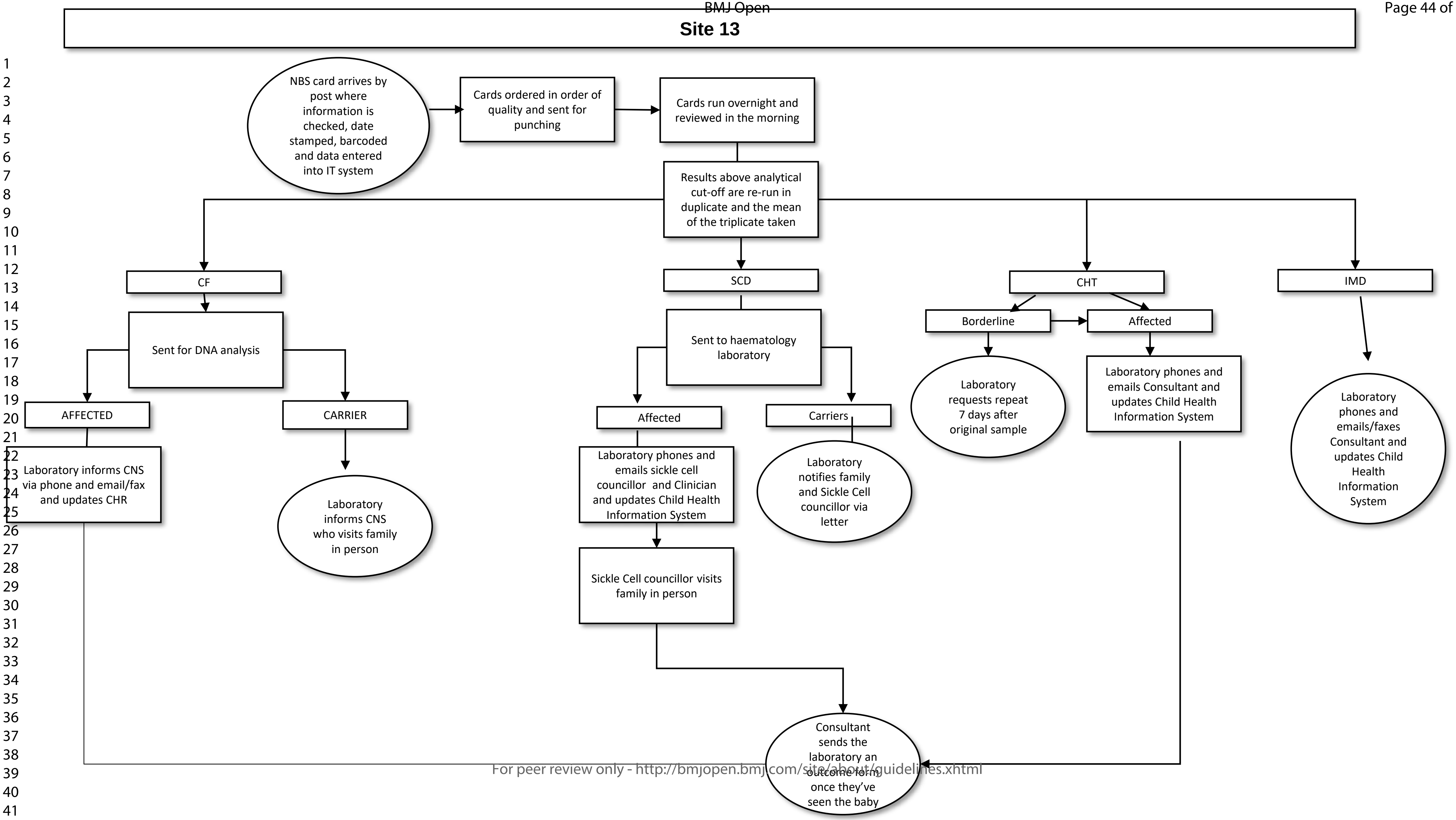
Site 13
NBS card arrives by post where information is checked, date stamped, barcoded and data entered into IT system
Cards ordered in order of quality and sent for punching
Cards run overnight and reviewed in the morning
Results above analytical cut-off are re-run in duplicate and the mean of the triplicate taken
CF
SCD
CHT
IMD
Sent for DNA analysis
AFFECTED
CARRIER
Laboratory informs CNS via phone and email/fax and updates CHR
Laboratory informs CNS who visits family in person
Sent to haematology laboratory
Affected
Carriers
Laboratory phones and emails sickle cell councillor and Clinician and updates Child Health Information System
Laboratory notifies family and Sickle Cell councillor via letter
Sickle Cell councillor visits family in person
Borderline
Affected
Laboratory requests repeat 7 days after original sample
Laboratory phones and emails Consultant and updates Child Health Information System
Laboratory phones and emails/faxes Consultant and updates Child Health Information System
Consultant sends the laboratory an outcome form once they've seen the baby

Consolidated criteria for reporting qualitative studies (COREQ): 32-item checklist

Developed from:
Tong A, Sainsbury P, Craig J. Consolidated criteria for reporting qualitative research (COREQ): a 32-item checklist for interviews and focus groups. *International Journal for Quality in Health Care*. 2007. Volume 19, Number 6: pp. 349 – 357

YOU MUST PROVIDE A RESPONSE FOR ALL ITEMS. ENTER N/A IF NOT APPLICABLE

No. Item	Guide questions/description	Reported on Page #
**Domain 1: Research team and reflexivity**		
Personal Characteristics		
1. Inter viewer/facilitator	Which author/s conducted the inter view or focus group?	Data Collection section
2. Credentials	What were the researcher's credentials? E.g. PhD, MD	PhD
3. Occupation	What was their occupation at the time of the study?	Children's Nurse (JC) and
4. Gender	Was the researcher male or female?	Female
5. Experience and training	What experience or training did the researcher have?	MSc Research Methods; extensive clinical experience; interview techniques.
Relationship with participants		
6. Relationship established	Was a relationship established prior to study commencement?	No
7. Participant knowledge of the interviewer	What did the participants know about the researcher? e.g. personal goals, reasons for doing the research	Professional role – Children's nurse, researcher role in this study
8. Interviewer characteristics	What characteristics were reported about the inter viewer/facilitator? e.g. Bias, assumptions, reasons and interests in the research topic	Positionality and reflexivity section
**Domain 2: study design**		
Theoretical framework		
9. Methodological orientation and Theory	What methodological orientation was stated to underpin the study? e.g. grounded theory, discourse analysis, ethnography, phenomenology, content analysis	Qualitative exploratory design
Participant selection		

For peer review only

10. Sampling	How were participants selected? e.g. purposive, convenience, consecutive, snowball	Purposive followed by snowball
11. Method of approach	How were participants approached? e.g. face-to-face, telephone, mail, email	Email
12. Sample size	How many participants were in the study?	Seventy-one
13. Non-participation	How many people refused to participate or dropped out? Reasons?	Four declined– time constraints / workload demands
Setting		
14. Setting of data collection	Where was the data collected? e.g. home, clinic, workplace	Workplace
15. Presence of non-participants	Was anyone else present besides the participants and researchers?	No
16. Description of sample	What are the important characteristics of the sample? e.g. demographic data, date	Staff involved in communicating the initial positive NBS result in the preceding 6 months.
Data collection		
17. Interview guide	Were questions, prompts, guides provided by the authors? Was it pilot tested?	Yes
18. Repeat interviews	Were repeat inter views carried out? If yes, how many?	N/A
19. Audio/visual recording	Did the research use audio or visual recording to collect the data?	Audio recording
20. Field notes	Were field notes made during and/or after the inter view or focus group?	Yes
21. Duration	What was the duration of the inter views or focus group?	10.40-54.58 mins
22. Data saturation	Was data saturation discussed?	Yes
23. Transcripts returned	Were transcripts returned to participants for comment and/or correction?	No but a staff meeting was held after all interviews had been conducted as per the protocol
**Domain 3: analysis and findings**		
Data analysis		
24. Number of data coders	How many data coders coded the data?	Two
25. Description of the coding tree	Did authors provide a description of the coding tree?	Yes
26. Derivation of themes	Were themes identified in advance or derived from the data?	Derived from the data
27. Software	What software, if applicable, was used to manage the data?	N/A
28. Participant checking	Did participants provide feedback on the findings?	Yes

For peer review only

Reporting		
29. Quotations presented	Were participant quotations presented to illustrate the themes/findings? Was each quotation identified? e.g. participant number	Results section
30. Data and findings consistent	Was there consistency between the data presented and the findings?	Yes
31. Clarity of major themes	Were major themes clearly presented in the findings?	Results
32. Clarity of minor themes	Is there a description of diverse cases or discussion of minor themes?	Discussion

Once you have completed this checklist, please save a copy and upload it as part of your submission. When requested to do so as part of the upload process, please select the file type: *Checklist*. You will NOT be able to proceed with submission unless the checklist has been uploaded. Please DO NOT include this checklist as part of the main manuscript document. It must be uploaded as a separate file.

For peer review only
